# Activated Sludge in the Bioremediation of Water Containing SARS-CoV-2 Antivirals

**DOI:** 10.3390/jox16040134

**Published:** 2026-07-22

**Authors:** Dora Lastovčić, Ivona Zirn, Tijana Jezerčić, Kristina Bule Možar, Luka Večenaj, Matija Cvetnić, Magdalena Ujević Bošnjak, Marinko Markić, Tomislav Bolanča, Šime Ukić, Dajana Kučić Grgić

**Affiliations:** 1 Faculty of Chemical Engineering and Technology, University of Zagreb, Trg Marka Marulića 19, 10000 Zagreb, Croatia; izirn@fkit.unizg.hr (I.Z.); tjezercic@fkit.unizg.hr (T.J.); kbule@fkit.unizg.hr (K.B.M.); lvecenaj@fkit.unizg.hr (L.V.); mcvetnic@fkit.unizg.hr (M.C.); mmarkic@fkit.unizg.hr (M.M.); tbolanca@unizg.hr (T.B.); sukic@fkit.unizg.hr (Š.U.); 2 Croatian Institute of Public Health, Rockefellerova 7, 10000 Zagreb, Croatia; magdalena.ujevic@hzjz.hr

**Keywords:** SARS-CoV-2 antivirals, activated sludge, optimization, bioremediation, response surface methodology, *Aliivibrio fischeri*

## Abstract

The increasing presence of SARS-CoV-2 antivirals (SASs) in wastewater threatens aquatic ecosystems, making it necessary to better understand their removal efficiency during biological treatment. This study evaluates the removal percentages, kinetic mechanisms, and toxicity profiles of six SASs, daclatasvir (DCV), darunavir (DRV), favipiravir (FAV), lopinavir (LOP), remdesivir (REM), and ritonavir (RIT), using activated sludge. A full factorial design combined with response surface methodology (RSM) was used to assess the impact of pH, temperature (*T*), and mixed liquor suspended solids (MLSS) on SAS removal and microbial stability. The results distinguished two dominant removal pathways. DCV and LOP were primarily removed by adsorption, whereas DRV, FAV, REM and RIT were predominantly removed through biodegradation involving enzymatic transformation and bacterial co-metabolism. RSM achieved a desirability score of 0.772 and identified the optimal operating conditions (pH = 6.08, MLSS = 4.41 g/L, and *T* = 44.99 °C), resulting in removal efficiencies above 93% for all compounds except DRV, which remained the limiting compound with a maximum removal efficiency of 66%. Perturbation analysis showed that all tested factors have a distinct influence on process performance. These established kinetic trends should support further investigations into advanced biological wastewater treatment methods and precise ecotoxicity profiling of emerging biotransformation products.

## 1. Introduction

Severe Acute Respiratory Syndrome Corona Virus 2 (SARS-CoV-2), first identified in Wuhan, China, represents a global health threat and has caused millions of deaths worldwide, with many individuals continuing to experience long-term health consequences. In addition to the disease itself, a major threat to humanity is the widespread use of antiviral substances (SASs) [1]. Given the urgent need for effective interventions during the pandemic and the time required for vaccine development, researchers focused on repurposing existing SASs previously used to treat other viruses. This approach enabled the rapid evaluation of SASs with already established safety profiles. Clinical reports have demonstrated promising therapeutic efficacy for several SASs, including daclatasvir (DCV), darunavir (DRV), favipiravir (FAV), lopinavir (LOP), remdesivir (REM), tenofovir disoproxil fumarate, azithromycin, ritonavir (RIT), chloroquine, baricitinib, and cefarantin [1,2,3,4].

The increased use of SASs has led to higher concentrations in aquatic ecosystems, primarily due to incomplete metabolism in patients and improper waste disposal. According to the World Health Organization, between 2020 and 2022, more than 23,000 tons of pharmaceuticals were consumed worldwide for the treatment of COVID-19, with approximately 30–45% entering the aquatic environment through metabolism or improper disposal [5]. Consequently, the concentrations of SASs detected in wastewater and receiving surface waters have increased considerably, with wastewater concentrations estimated to have risen by as much as 70% during the pandemic [2]. The widespread occurrence of these compounds has raised concerns regarding their potential adverse effects on aquatic ecosystems and human health [2,5]. Therefore, further research on the occurrence of SASs in aquatic environments and the development of effective removal strategies is urgently needed [2,3].

An additional environmental concern is that biological and physicochemical degradation of SASs often does not result in complete mineralization but instead produces transformation products that may differ from the parent compounds in terms of persistence and toxicity [6,7]. For example, studies on oseltamivir ethylester and its metabolite oseltamivir acid demonstrated additive toxicity toward *Aliivibrio fischeri*, highlighting the importance of assessing the toxicity of pharmaceutical mixtures rather than individual compounds alone [8]. Similarly, studies on ribavirin using zebrafish embryos indicated that the toxicity of treated effluents should be considered during environmental risk assessment, even after advanced oxidation processes [9,10]. Furthermore, conventional biological treatment may generate persistent metabolites, as demonstrated for acyclovir, whose biotransformation product, carboxy-acyclovir, has been detected in finished drinking water following conventional wastewater treatment [11]. These findings emphasize that evaluating the environmental fate of SASs requires not only determining the removal of parent compounds but also considering the formation and potential ecotoxicity of their transformation products.

The SASs investigated in this study are well-established antiviral agents used for the treatment of various viral infections. DCV is an approved SAS for treating hepatitis C infection and has also been tested against COVID-19, where it showed positive effects. In addition to its antiviral efficacy, DCV exhibits broad-spectrum antimicrobial activity [5]. Similarly, DRV is an antiviral drug that acts as a protease inhibitor and is intended for the treatment of human immunodeficiency virus (HIV). It was approved in February 2007 for use in HIV-infected patients not receiving antiretroviral therapy [6]. FAV is a relatively new, low-molecular-weight SAS that has shown activity against many types of RNA viruses, including SARS-CoV-2 [7]. However, research conducted in Turkey showed that less than 55% of FAV is removed in wastewater treatment plants (WWTPs) [8]. Another well-known SAS, LOP, was widely used to treat HIV infections before the COVID-19 pandemic [9]. REM is a broad-spectrum prodrug and a small-molecule adenosine nucleotide analog that has shown activity against RNA viruses in several families. It was originally developed to treat Ebola virus infection [10]. In clinical guidelines, REM was the first treatment for COVID-19 approved by the US Food and Drug Administration (FDA) and is currently the only recommended SAS for use in hospitalized patients with COVID-19 [10,11]. Finally, RIT is often used as a pharmacokinetic enhancer in antiviral therapies because it increases the bioavailability of concomitant SASs. Decades of experience with RIT-boosted HIV therapies and, more recently, COVID-19 therapies show that RIT doses are well tolerated and have an established safety profile [12]. These SASs represent a broad spectrum of physicochemical properties, including differences in hydrophobicity, molecular structure, and ionization behavior, which are expected to influence their environmental fate and removal mechanisms during biological wastewater treatment. Consequently, they provide a representative model set for evaluating the relative contributions of adsorption and biodegradation in activated sludge systems.

**Figure 1:**
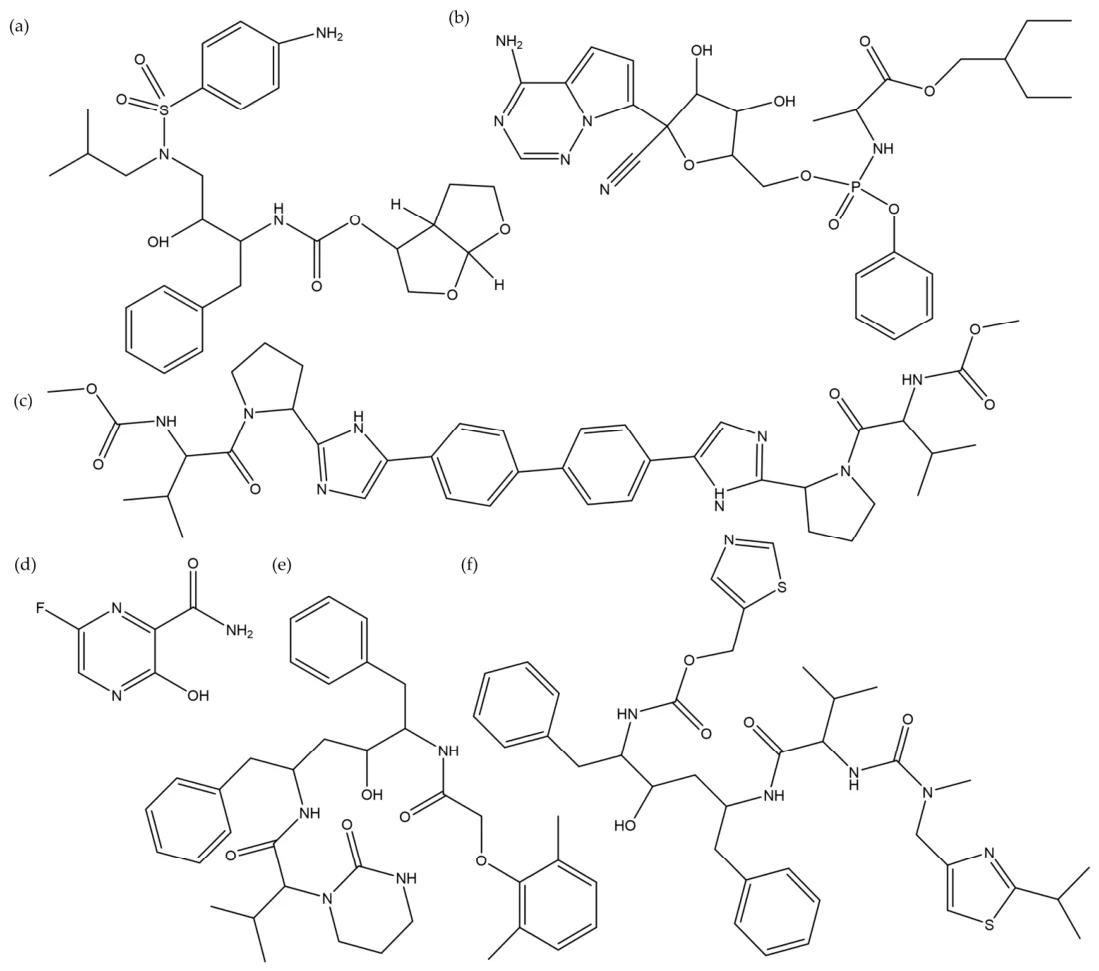
Chemical structures of the investigated SASs: (**a**) DRV, (**b**) REM, (**c**) DCV, (**d**) FAV, (**e**) LOP, and (**f**) RIT.

Despite their widespread use and documented presence in aquatic environments, the antiviral compounds under investigation are not currently included in the EU Watch List established under the Water Framework Directive for surface water monitoring, nor are they included in the Drinking Water Directive Watch List for monitoring drinking water quality [13]. Furthermore, they are not routinely monitored in conventional wastewater treatment systems, although recent studies increasingly classify pharmaceutical antivirals as emerging environmental contaminants due to their persistence and incomplete removal during wastewater treatment [13,14]. In conventional WWTPs, the removal efficiency of various SASs is estimated to be low (<20%), resulting in the presence of drug residues and their metabolites in secondary effluents [15]. The high predicted no-effect concentration (PNEC) values for LOP and RIT (in ng/L) indicate significant chronic toxicity to aquatic organisms. This demonstrates that LOP and RIT are currently priority SASs that must be thoroughly monitored and effectively removed from all water and wastewater samples [15,16]. Given that the environmental burden of various pharmaceuticals has been a long-standing concern, several treatment methods, including oxidation, biodegradation, and adsorption, have been used to remove these contaminants from wastewater [17]. Although several advanced technologies have been developed for further purification of water treated in WWTPs, the costs of installing and maintaining these systems are not acceptable in many countries. Consequently, improving the biodegradation potential of activated sludge through optimization of operational conditions and the enrichment or adaptation of microbial communities has emerged as a cost-effective alternative to implementing additional treatment technologies [18]. Such strategies enhance the metabolic capacity of the existing biomass toward persistent pharmaceutical contaminants while preserving the current treatment infrastructure. However, their effectiveness largely depends on the susceptibility of individual contaminants to microbial transformation and on their potential inhibitory effects on the activated sludge microbiome. SASs represent a particularly challenging group of emerging contaminants because of their complex chemical structures (Figure 1) and specific biological activity. In particular, HIV protease inhibitors such as LOP and RIT are specifically designed to inhibit viral protease enzymatic activity [19]. Consequently, they may also interfere with key bacterial enzymes involved in activated sludge biodegradation, suppress microbial metabolic activity, and reduce treatment efficiency. Therefore, achieving satisfactory removal efficiency requires optimization of the activated sludge process, including operating conditions and microbial adaptation, to enhance the resilience and degradative capacity of the microbial community [20].

This study focuses on optimizing the removal process of six structurally diverse SARS-CoV-2 antivirals (DCV (C_40_H_50_N_8_O_6_), DRV (C_27_H_37_N_3_O_7_S), FAV (C_5_H_4_FN_3_O_2_), LOP (C_37_H_48_N_4_O_5_), RIT (C_37_H_48_N_6_O_5_S_2_), and REM (C_27_H_35_N_6_O_8_P)) using activated sludge under controlled laboratory conditions to better understand the mechanisms governing their removal and to identify optimal process conditions. To achieve reliable determination of removal kinetics, distinguish between adsorption and biodegradation processes, and enable robust statistical evaluation of operational parameters, laboratory-scale studies commonly employ pharmaceutical concentrations higher than those typically detected in municipal wastewater. Nevertheless, identifying the optimal operating conditions for activated sludge treatment remains challenging, as biological wastewater treatment is governed by multiple simultaneously acting physicochemical and biological factors. Changes in pH, temperature and biomass concentration not only influence microbial metabolism and enzymatic activity but also alter the physicochemical behavior of pharmaceuticals, including their ionization state, hydrophobicity, and bioavailability. As a result, the effects of these operational parameters are frequently interactive rather than independent, making conventional one factor at a time optimization insufficient for accurately describing process performance. Response surface methodology (RSM) has therefore become a widely applied multivariate approach for evaluating factor interactions and identifying optimal operating conditions in complex biological treatment systems [21,22,23].

## 2. Materials and Methods

### 2.1. Activated Sludge Conditioning

The activated sludge used in the laboratory tests was collected as a fresh sample from the biological treatment stage of a municipal WWTP in Zagreb, Croatia. The physical and chemical characterization followed the methodology described in Lastovčić et al. [24]. Prior to the optimization experiments, the activated sludge was acclimated for 24 h under aerobic conditions in mineral medium containing the SASs investigated to promote microbial adaptation and minimize potential metabolic shock. Acclimation was performed at room temperature (25 ± 2 °C) with continuous shaking at 160 rpm. During acclimation period, pH and dissolved oxygen (DO) concentrations were monitored at predefined time intervals using the corresponding electrodes. No significant variations were observed, indicating stable acclimation conditions and the absence of measurable inhibition of microbial activity during the adaptation stage. After acclimation, the sludge was washed three times with distilled water, concentrated by decantation and centrifugation, and subsequently used for the experiments. The concentrations of the investigated SASs in the supernatant were determined by UHPLC analysis after the washing procedure, confirming that no residual antivirals remained before the sludge was used in the biodegradation experiments.

### 2.2. Composition of Mineral Medium

To preserve microbial activity, tests were conducted in mineral medium prepared according to Kyaw et al. [25], with the following composition per liter: 12.5 g K_2_HPO_4_, 3.8 g KH_2_PO_4_, 1 g (NH_4_)_2_SO_4_, 0.1 g MgSO_4_ · 7 H_2_O, and 5 mL of trace elements containing H_3_BO_3_, ZnSO_4_ · 6 H_2_O, FeSO_4_(NH_4_)_2_SO_4_ · 6 H_2_O, CoSO_4_ · 7 H_2_O, (NH_4_)_6_Mo_7_O_24_ · 4 H_2_O, CuSO_4_ · 5 H_2_O, and MnSO_4_ · 4 H_2_O. Because microbial growth is generally influenced by pH, biodegradation was tested at pH 5.5, 7.0, and 8.5. The optimal pH for bacterial growth is considered to be between 6.5 and 7.5 [23], so the target values were determined by lowering and raising the pH by one unit. Although larger differences could provide a wider optimization design space, most bacteria do not tolerate pH values higher than 9.5 or lower than 4.0 [23], and these levels are also not expected in wastewater treatment plants (raw wastewater, pH = 6.0–8.0) [26]. Therefore, pH values of 5.5, 7.0, and 8.5 were considered appropriate for this study. For this purpose, the Henderson–Hasselbalch equation [27] was used to calculate the required molar ratios and mass measurements (Equation (1), Table 1) for the phosphate buffer (Equation (2)) at pH 7.0 and 8.5. For the solution at pH 5.5, the equation was used only to determine the theoretical composition, as this value is outside the optimal buffering range of the phosphate system (p*K*_a_ ± 1.00). The final pH was adjusted using 1.000 mol/L HCl or 1.000 mol/L KOH.

(1) $$pH=pK_{a}+log_{10}\left(\frac{\left[A^{-}\right]}{\left[HA\right]}\right)$$

(2) $$H_{2}{PO}_{4}^{-}⇌H{PO}_{4}^{2-}+H^{+}\left(pK_{a}=7.20\right)\;$$

### 2.3. Preparation of SAS Stock Solutions

Stock solutions for this study were prepared in the appropriate mineral medium using high-purity SAS standards: DCV (98%, p.a., BLD Pharmatech Ltd., Shanghai, China), DRV (≥98% p.a., Sigma Aldrich, St. Louis, MO, USA), FAV (98% p.a, Fluorochem Limited, Hadfield, UK), LOP (≥98% p.a., Sigma Aldrich, St. Louis, MO, USA), REM (≥98% p.a., Sigma Aldrich, St. Louis, MO, USA), and RIT (98% p.a., Indagoo Research Chemicals, Madrid, Spain). The concentrations were determined according to the maximum solubility of each SAS in water: DCV at 100.000 μmol/L; DRV and FAV at 200.000 μmol/L; LOP, REM, and RIT at 2.000 μmol/L.

### 2.4. Preliminary MLSS/Mass Test

**Figure A1:**
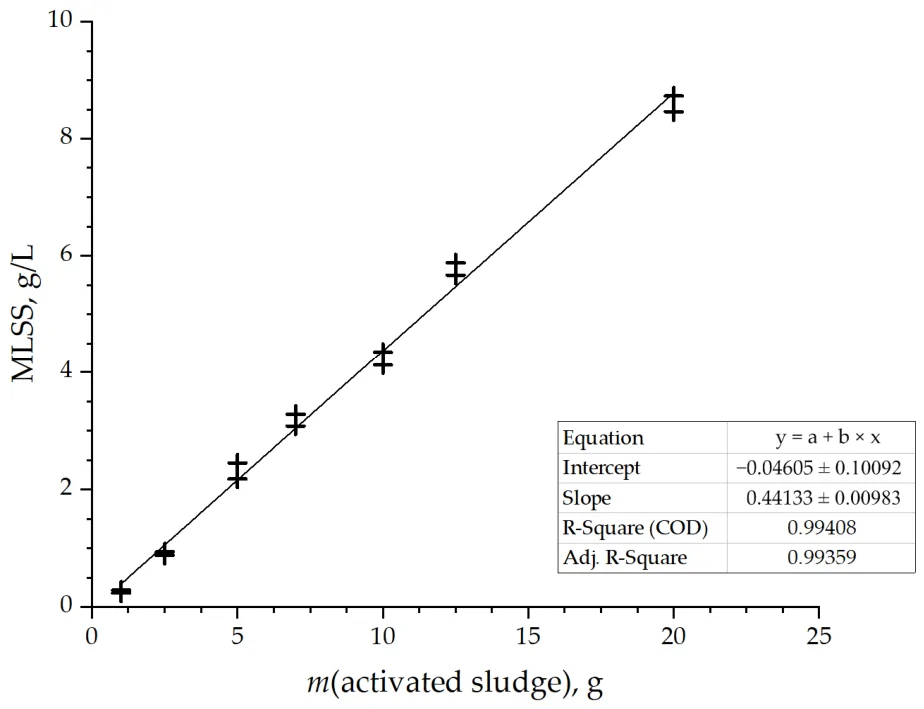
Calibration curve for the gravimetric determination of MLSS concentrations. In the equation, the multiplication symbol (×) denotes variable rates, whereas the addition symbol (+) represents fixed components.

After preparing the fresh activated sludge sample, a higher initial mixed liquor suspended solids (MLSS) was achieved by centrifugation (Universal 320/320R, Hettich^®^, Tuttlingen, Germany) at 3500 rpm and 0 °C for 10 min. Target MLSS concentrations (Table 2) were determined gravimetrically using a calibration curve (Figure A1) established with a series of mass measurements (*R*^2^ > 0.9900, *p* < 0.05), improving both accuracy and precision across the working range of 1 to 20 g of centrifuged activated sludge.

### 2.5. Experimental Setup and Statistical Procedure

**Figure 2:**
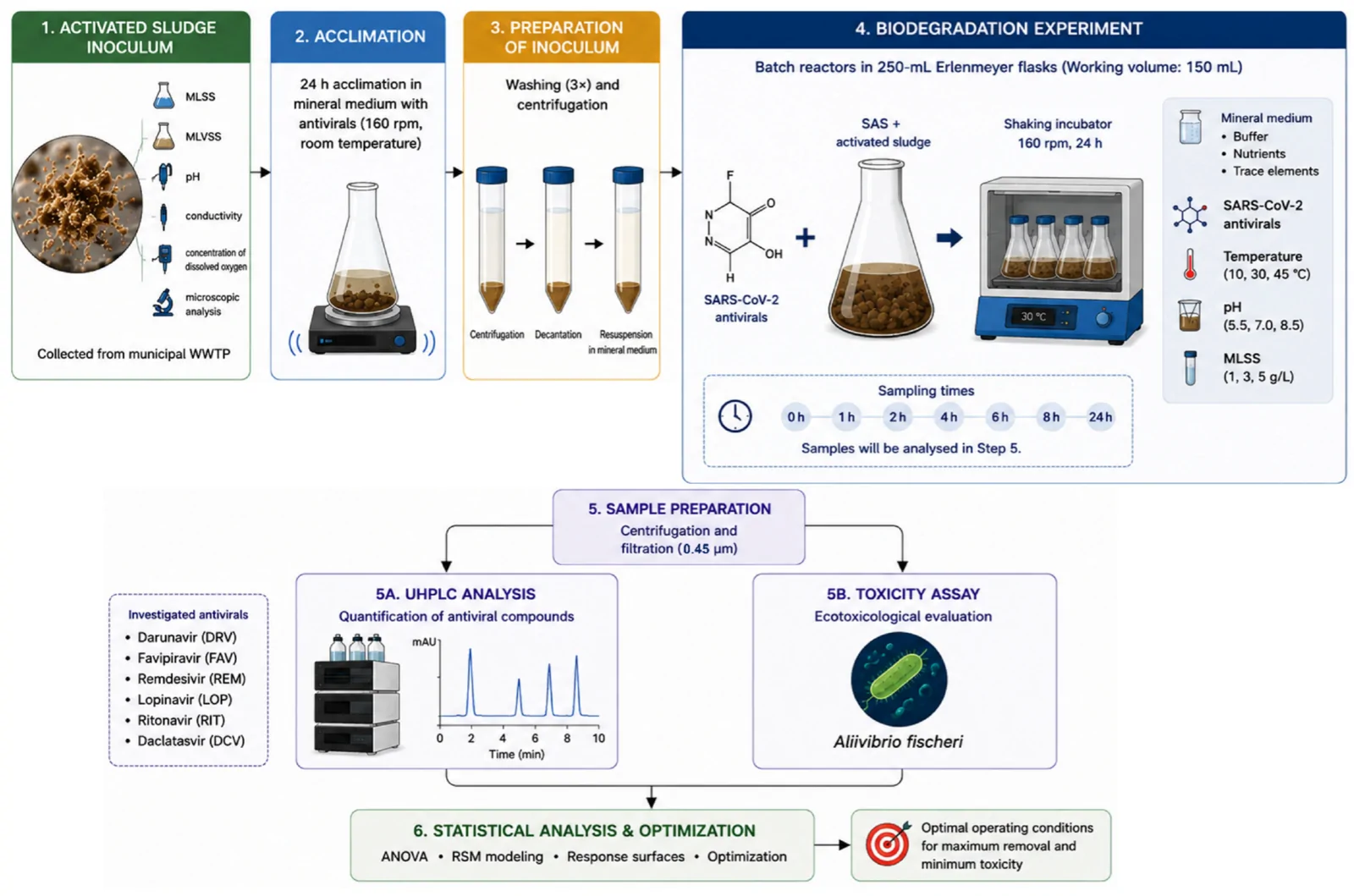
Schematic view of the optimization setup and monitored parameters.

The optimal conditions for bioremediation of the aforementioned SAS were determined using the experimental design shown in Table 3. Three factors were tested for each individual SAS in a full factorial design at three levels: pH (5.5, 7.0, and 8.5), activated sludge concentrations (1, 3, and 5 g/L), and temperatures (10, 30, and 45 °C). Preparation of the reaction flasks (T1–T27, Figure 2) involved weighing the appropriate amount of centrifuged activated sludge (Table 2), adding mineral medium adjusted to the desired pH (Table 1), and adding the required volume of the appropriate SAS stock solution to achieve a concentration of 1.000 μmol/L in a total working volume of 150 mL. Temperature was controlled in a shaking incubator (Biobase BJPX-SDT20, Biobase, Jinan, Shandong, China) with continuous shaking at 160 rpm for 24 h. All experiments were conducted in triplicate to ensure the reproducibility of the obtained results.

All responses (individual SAS removal, %) were modeled using RSM. To assess the validity and adequacy of each model, analysis of variance (ANOVA) was performed and fit statistics were examined. To test model normality and homogeneity, diagnostic tools such as normal probability plots of residuals and residuals-versus-predicted plots provided in Design Expert 12 software (Stat-Ease Inc., Minneapolis, MN, USA) were used. To obtain statistically valid models, each response dataset was evaluated for internally studentized residuals, externally studentized residuals, Cook’s distance, and influence on fitted values (DFFITS). An iterative outlier removal procedure was carried out based on numerical diagnostic criteria: the critical point exceeding the multicriteria statistical threshold was identified and excluded, the model was recalculated, and the process was repeated until the models were significant by ANOVA or until two points were excluded (<10% of the total 27 experiments). Additionally, Box-Cox plots were checked for recommendations on data transformation.

### 2.6. UHPLC Analysis

SAS concentrations were monitored at 1, 2, 3, 4, 6, 8, 12 and 24 h using a Vanquish Flex UHPLC system (Thermo Fisher Scientific, Waltham, MA, USA) equipped with a diode array detector (DAD FG, VF-D11-A-01). Before injection, each biodegradation sample was centrifuged at 2800 rpm for 5 min (FV-2400, Minicentrifuge-Vortex Microspin, SIA Biosan, Riga, Latvia) and filtered directly into the vials through a 0.45 μm sterile CA filter to remove solid sludge particles.

Previously developed isocratic methods described in Lastovčić et al. [1] were transferred to a UHPLC ACQUITY C18 column (1.7 μm, 2.1 ∙ 100 mm). The mobile phase consisted of acetonitrile (mobile phase B, %) and Milli-Q water with 0.1% formic acid (mobile phase A, %). The flow rate was set to 0.100 mL/min, with minor modifications to the mobile phase B for DCV, DRV, and LOP (Table 4). The injection volume was 10 μL, and the oven temperature was maintained at 40 °C.

Calibration standards for each SAS were prepared in methanol to ensure complete solubility, covering a concentration range of 0.002 to 3.000 μmol/L. To confirm valid quantification of SASs, precision, selectivity, matrix effects, and linearity were evaluated for each method. Triplicate measurements of standard solutions used for calibration curves were employed to assess precision by relative standard deviation (RSD). The maximum RSD for each method at the limits of quantification (LOQ) ranged from 5.49% (LOP) to 9.65% (REM), which is acceptable for environmental analysis according to guidelines for standard method performance requirements [28]. Method selectivity was confirmed by blank matrix analysis, which showed no interfering background signals at the targeted SASs retention times under the monitored wavelengths. Linearity was confirmed with a correlation coefficient *R*^2^ greater than 0.9900 (Table 4). All samples were analyzed in triplicate, and final concentrations were expressed as the arithmetic mean ± standard deviation. Data analysis was performed using Chromeleon 7 software (Thermo Fisher Scientific, Waltham, MA, USA).

### 2.7. Determination of MLSS and MLVSS

To monitor the biomass concentration and characteristics within the system, MLSS and mixed liquor volatile suspended solids (MLVSS) were determined gravimetrically at 6 and 24 h. To determine the MLSS of the activated sludge, membrane vacuum filtration was performed in parallel for each sample. The 0.45 μm membrane filters (MF, ReliaDisc™; Ahlstrom-Munksjö, Helsinki, Finland) were dried for 1 h at 105 °C, and the filtered sample (S) volume was 5 mL. After filtration, the wet filters were dried at 105 °C for 2 h. The sample mass (*m*(S, 105 °C)) was calculated as the difference between the mass of the dried membrane filter with substrate (*m*(MF + S)) and the initial filter mass (*m*(MF)). MLSS (g/L) was then calculated according to Equation (3):

(3) $$MLSS=\frac{m\left(S,105\;{}^{\circ}C\right)}{V}$$

For the activated sludge to be suitable for analysis, the organic fraction should be higher than 60% [29]. This is measured as MLVSS and determined using the dried filters from the MLSS procedure. The empty crucibles (C) were ignited to constant mass in a muffle furnace at 550 °C. The filters were then placed inside and ignited for 2 h at 550 °C to remove volatile organic matter from the substrate. The weighed mass of the sample after ignition (*m*(S, 550 °C)) was obtained by subtracting the mass of the ignited empty crucible (*m*(C)) from the mass of the crucible with substrate (*m*(C + S, 550 °C)). MLVSS (%) was then calculated using Equation (4): according to the general guidelines of Standard Methods 2540 D and E [30], respectively.

(4) $$MLVSS=\frac{\;m\left(S,105\;{}^{\circ}C\right)-m\left(S,550\;{}^{\circ}C\right)}{m(S,105{}^{\circ}C)}\cdot 100$$

Both analytical procedures were strictly performed in accordance with the general guidelines of Standard Methods 2540 D and E [30], respectively. All determinations were carried out in duplicate.

### 2.8. Monitoring of pH, Dissolved Oxygen, and EC

To monitor the basic physicochemical properties during the experiment, it was necessary to measure the pH, DO concentration, and electrical conductivity (EC) of the samples using appropriate electrodes. These parameters were measured with a pH electrode, a DO electrode, and an EC electrode connected to the WTW Multi 340i (WTW, Xylem Analytics Germany, Weilheim in Oberbayern, Germany).

### 2.9. Acute Toxicity Assessment

The ecotoxicity assessment was conducted on *Aliivibrio fischeri* to evaluate the potential toxicological impact of the investigated SASs after 24 h biodegradation. Bioluminescence inhibition test followed the procedure described in ISO 11348-1:2007, Water Quality-Determination of the inhibitory effect of water samples on the light emission of *Aliivibrio fischeri* (luminescent bacteria test) [31].Tests were conducted on initial SAS solutions and 24 h samples from completed experiments. All samples containing activated sludge were filtered through 0.45 μm membrane filters. To prevent retention and carryover of SAS residues, filters were used once per sample, and filter conditioning with distilled water was performed before each filtration. The pH was adjusted to 7.00 ± 0.20 with 1.000 mol/L HCl or 1.000 mol/L NaOH to ensure optimal conditions for the bacteria and to eliminate negative pH effects. The tested bacteria were subcultured for 24 h on a selective growth medium for *Aliivibrio fischeri* before the toxicity assay. Bacterial luminescence was recorded using a LUMIStox 300 luminometer (Dr. Lange GmbH, Hannover, Germany) connected to a LUMIStherm thermostat for temperature control at 15 °C. For reliable and comparable results, all initial luminescence (*L*_0_) measurements were approximately 3000 relative light units. A linear dilution series was prepared for each sample with 2% NaCl solution as the diluent and as the control. Each sample was tested by measuring bacterial luminescence after 30 min of exposure (*L*_30_). A correction factor was applied to adjust *INH* by taking the ratio between the *L*_0_ and *L*_30_ values of the control. Luminescence inhibition (*INH*, %) was calculated according to Equation (5):

(5) $$INH=\frac{L_{0}\cdot f-L_{30}}{L_{0}\cdot \;f}\cdot 100$$

The sample concentrations causing 20% (*EC*_20_) and 50% (*EC*_50_) inhibition of bacterial luminescence were estimated according to the linear dependence of ln*γ* and ln(SAS (linear dilution)). Factor *γ* (%) was calculated according to Equation (6):

(6) $$\gamma =\frac{INH}{\left(100-INH\right)}$$

All ecotoxicity bioassays were performed in triplicate. The mineral medium used in the experiments was also tested and showed no inhibition of *Aliivibrio fischeri* bioluminescence, contributing no toxicity to SAS samples.

### 2.10. Testing of Adsorption Properties

To distinguish microbial degradation from physicochemical processes in SAS removal, additional adsorption tests on sludge flocs were conducted in parallel with biodegradation experiments (up to 24 h). Activated sludge was characterized, washed three times with distilled water, concentrated, and sterilized to eliminate any active microbial processes. Double sterilization was performed by autoclaving the activated sludge at 121 °C under 1 atm overpressure. These abiotic controls were performed in triplicate under the central point conditions (0, 0, 0) to clarify the overall contribution or absence of adsorption to SAS removal and to further support the biodegradation optimization experiments. Abiotic controls were prepared and analyzed in the same way as the SAS experimental flasks described in Section 2.5, with the only difference being the addition of sterilized activated sludge, as previously described.

Because the optimal process conditions for the efficiency of each SAS were determined based on removal within 24 h, abiotic controls at 1 h and 24 h were used to differentiate and support the baseline SAS mechanisms at the central point conditions, in relation to the available literature data and log *p* characteristics. Equation (7) was used to calculate abiotic recovery (%) for each SAS, with *t* indicating time.

(7) $$SAS\;abiotic\;recovery=\frac{c_{control}(t)}{c_{0}}\cdot 100$$

### 2.11. Data Analysis

The experimental design and optimization solutions were developed with the Design-Expert 12 software package. Experimental data were processed using OriginPro 8.5 software (OriginLab Corporation, MA, USA). Means and corresponding standard deviations were calculated from biological triplicates.

## 3. Results

### 3.1. Activated Sludge Characterization

To evaluate the suitability of the activated sludge for the bioremediation experiments, the main physicochemical parameters were determined (Table 5), including pH, temperature, DO, EC, water content, dry matter, volatile matter, MLSS, and MLVSS. Slightly higher standard deviations were observed because sludge samples were collected over an extended period, reflecting normal seasonal variability in wastewater treatment plant operation. Nevertheless, all measured parameters remained within the expected ranges for municipal activated sludge. The sludge exhibited neutral pH (7.34 ± 0.51), adequate oxygen availability (3.30 ± 1.17 mg/L), and a high volatile solids content (64.29 ± 6.09%), indicating an active microbial biomass suitable for biological treatment. Furthermore, the initial MLSS concentration (4.34 ± 0.22 g/L) was within the investigated optimization range, while the determined dry matter content enabled accurate preparation of the target biomass concentrations for the adsorption experiments described in Section 2.10 (129 mg for 3 g/L under the central point conditions (0, 0, 0)). Overall, these results confirmed that the activated sludge possessed appropriate physicochemical characteristics for evaluating the removal of SARS-CoV-2 antivirals under controlled experimental conditions.

### 3.2. Microscopic Examination of the Activated Sludge

**Figure 3:**
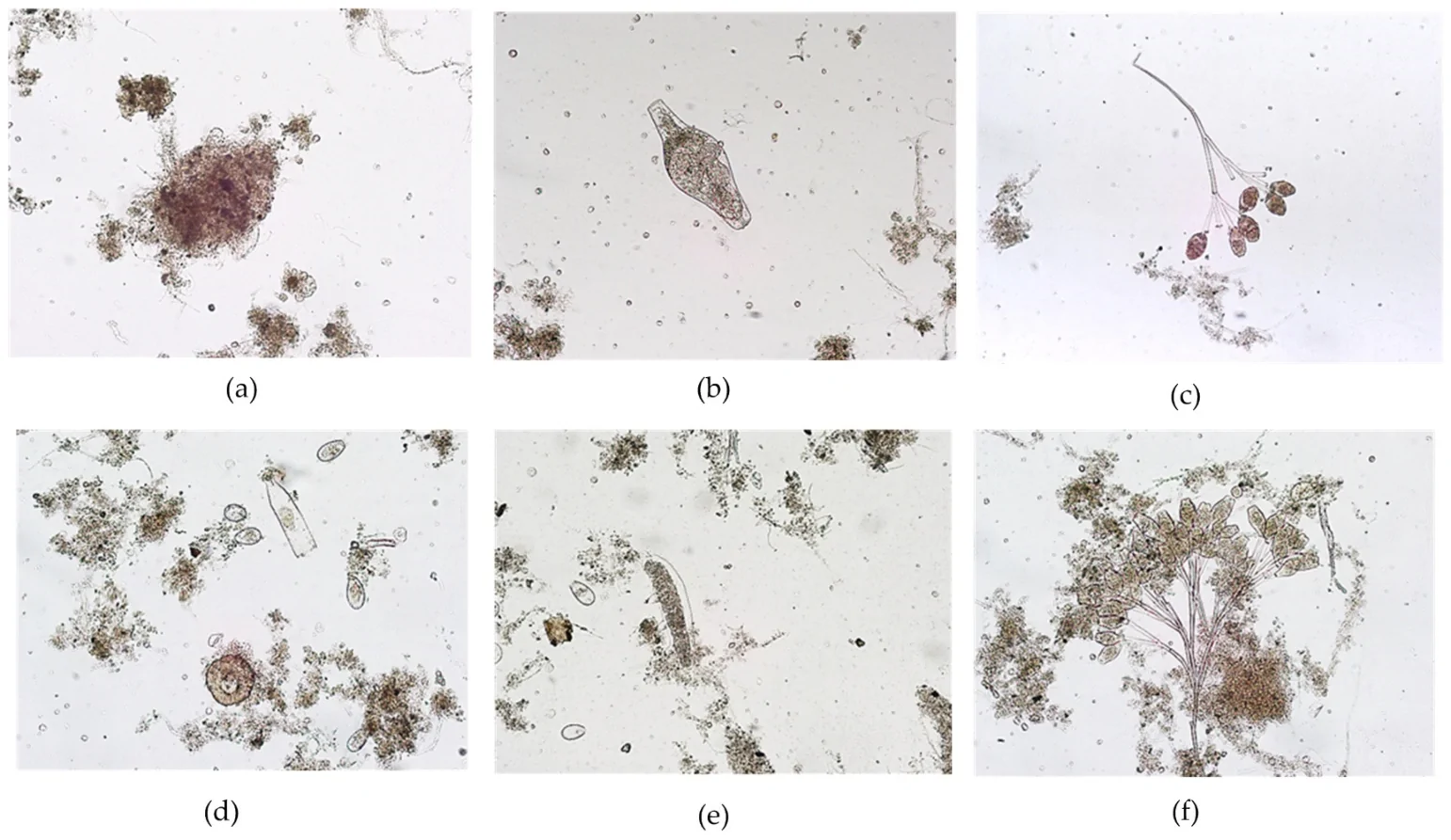
Characteristic floc structures and microorganisms in activated sludge during the experiments, analyzed by light microscopy (100×): (**a**) activated sludge flocs; (**b**) Rotifera; (**c**) ciliates; (**d**) various testate amoebae; (**e**) Tardigrada; (**f**) stalked ciliates.

**Figure 4:**
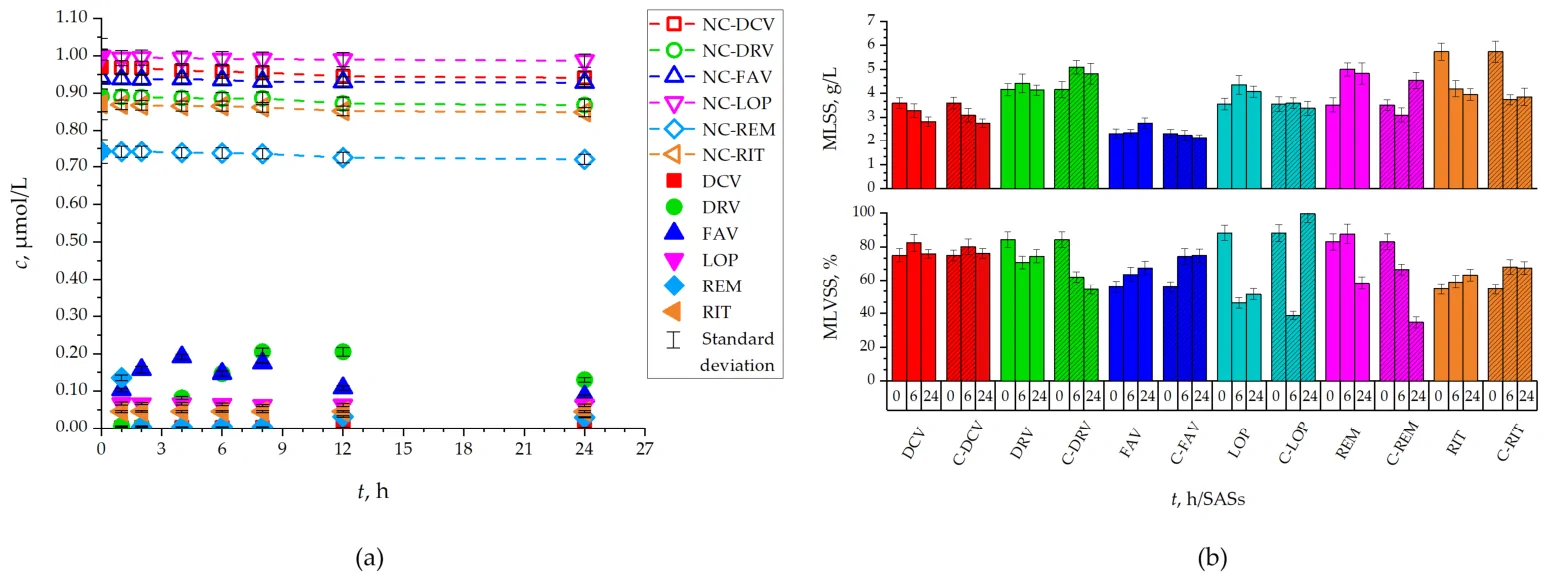
Evaluation of SASs removal kinetics and biomass stability under central point conditions (0, 0, 0): (**a**) high-frequency removal kinetics of the target SASs at 0, 1, 2, 4, 6, 8, 12, and 24 h; (**b**) dynamic changes in the MLSS and MLVSS at 0 h, 6 h, and 24 h. Values represent mean ± SD (*n* = 3). NC: negative control; C: control.

Throughout the experiments, the characteristics of activated sludge flocs and the presence of microorganisms were evaluated by light microscopy (100×, Olympus B201, Tokyo, Japan). Figure 3 presents six micrographs representative of all optimization experiments. Well-defined and compact activated sludge flocs were observed under all experimental conditions (Figure 3a). This was consistent with the relatively stable MLVSS fraction, which ranged between 60 and 80% throughout the experiments (Figure 4b). Microscopic observations revealed the presence of Rotifera (Figure 3b), stalked ciliates (Figure 3c,f), amoebae (Figure 3d), Tardigrada (Figure 3e), and several types of testate amoebae. These microorganisms are typically found in mature activated sludge with longer sludge retention times and are generally associated with stable biological treatment processes. In particular, stalked ciliates contribute to improved effluent quality by grazing on dispersed bacteria and promoting floc clarification, while rotifers are commonly observed under conditions of low organic loading and well-established sludge. Testate amoebae are frequently reported in biologically stable systems and have been proposed as useful bioindicators of sludge maturity and treatment performance. The observed microfauna therefore suggests the presence of a well-developed and diverse activated sludge community suitable for the biodegradation experiments conducted in this study [32].

### 3.3. Evaluation of Central Point (0, 0, 0) Removal Kinetics

For easier interpretation of the results, Figure 4 shows the kinetics of SAS removal and the corresponding biomass stability (MLSS, MLVSS), while Table 6 presents abiotic recoveries of individual SASs for the central point conditions (0, 0, 0) listed in Table 3 in Section 2.5. All trends and percentages for the other conditions are listed in Table A1, Table A2 and Table A3, and can also be interpreted and described based on these representative results. Figure 4a presents a removal kinetics plot comparing the SAS samples to the negative controls, showing the degradation of each molecule (<10%) in mineral medium over time without the influence of activated sludge. The experiments were conducted over 24 h, as the effluent in conventional WWTPs is not retained longer than that. Figure 4b shows a grouped column plot comparing MLSS to its corresponding MLVSS for the 6th and 24th h of the experiment. This graphical representation helps visualize the relationship between sludge characteristics in the process and the detected SAS concentrations. In contrast, Table 6 provides insight into the hydrophobicity (log *p*) and adsorption potentials (abiotic control recoveries) of SASs, which are necessary to assess the primary mechanism of the removal process.

Direct comparison of the samples with the controls shows an overall high removal ratio for all SASs after 24 h (>90%) from the analyzed aqueous phase. The removal kinetics indicate that within the first 3 h of exposure to activated sludge, all SASs were removed from the solution, suggesting successful primary biodegradation or adsorption to the flocs, depending on each SAS’s molecular properties and affinities. After 24 h, all concentrations remained low, and the elevated concentrations of DRV and FAV showed a decreasing trend after the 12th hour. Referring to Table 6, with abiotic recoveries, in the 1 h of the process most SASs (FAV, LOP, REM, RIT) showed high adsorption potential with low recoveries in the range of 4.60–10.68%. While DCV showed no recovery at all, DRV in contrast exhibited moderate initial recovery of 40.95% on average. At the 24 h, final abiotic recoveries were higher, around 40–50% for DRV and REM, moderate for FAV (20.48%), very low for LOP (7.22%), none for DCV, and exceptionally high for RIT (91.13%). This suggests rapid adsorption in the initial hours of the process followed by subsequent desorption, which also depends on each log *p* value indicating each SAS’s hydrophobicity (Table 6). DRV, FAV, and REM have lower log *p* values (0.4–−2.82) than the other studied SASs (3.91–5.22), which suggests a lower binding potential to activated sludge and a higher potential for biodegradation, while adsorption is considered more likely for SASs with higher log *p* (DCV, LOP, RIT). These observations match the data measured within the 1 h according to Table 6, indicating that the first phase (1 h) of the process is primarily controlled by the hydrophobic or hydrophilic properties of the molecules. However, after the first phase, deviations in the second phase (24 h) seem to increase in terms of log *p*, likely influenced by another factor yet to be determined, such as distinct ionization states and molecular structures of the tested SASs. Therefore, SAS kinetics and biomass stability were further analyzed as parts of two groups divided by the dominant removal process based on our results: dominant biodegradation (group A) and dominant adsorption onto the sludge (group B). In simple terms, SASs in group A are broken down by microorganisms, while SASs in group B are removed by physically sticking to solid surfaces of the activated sludge.

#### 3.3.1. Group A—Biodegradation

The observed decreases in DRV, FAV, REM, and RIT concentrations over time (>90%), together with high abiotic recoveries after 24 h (20–90%) under central point conditions, were directly associated with active biodegradation during the removal of these SASs. This is strongly suggested for RIT, given that both abiotic recovery and removal exceeded 90% of the initial concentration. DRV and REM showed average recoveries of 40%, while FAV showed 20%, indicating that only part of the substrate concentrations in the aqueous phase was available for biodegradation by microorganisms. These observations are further supported by MLSS and MLVSS analyses. MLSS values remained constant for REM, while DRV, FAV, and RIT showed lower MLSS than the controls. This may indicate a toxic effect of these SASs on specific microorganisms despite the overall effective biodegradation. The corresponding MLVSS values and standard deviations (SD, *n* = 3) reflect the organic fraction of the sludge, which, for RIT, remained at the same level as in the controls after 24 h. For DRV and REM, the biomass increased by 10–20% compared to the controls. Although biomass yield coefficients were not determined in the present study, the observed MLVSS trends support the hypothesis that biodegradation contributed to the removal of DRV and REM. Given the low concentrations of the investigated SASs, these compounds most likely acted as co-metabolic substrates rather than primary carbon sources, with their transformation occurring simultaneously with the utilization of endogenous organic matter. A negative effect was observed for FAV, with a decrease in MLVSS of about 15% compared to the controls. This may also be related to the toxic effect of this SAS on microorganisms in the activated sludge, which will be discussed further.

#### 3.3.2. Group B—Adsorption

High removal of DCV and LOP (>90%) after 24 h indicated that a dominant mechanism other than biodegradation was present. As their abiotic controls showed low desorption trends (below 10% for LOP) and none at all for DCV during the entire test period, biodegradation appeared to be a less likely process. In these cases, adsorption to activated sludge flocs is potentially the dominant mechanism controlling the overall removal of DCV and LOP, which is also supported by their high log *p* values. MLSS and MLVSS after 24 h showed no changes for DCV, indicating that DCV had no effect on the biomass, while LOP had a negative effect by decreasing MLSS and significantly lowering MLVSS by around 50%. These results show that LOP exerted a substantial inhibitory effect on the tested biomass and may induce a highly toxic effect in *Aliivibrio fischeri* bioluminescence tests.

### 3.4. Measurement of pH, DO, and EC

Levels of pH, DO, and EC measured in T1–T27 for each studied SAS are presented in Table A7, Table A8 and Table A9. Because the mineral medium was prepared as a buffer solution described in Section 2.2, pH levels were controlled during the 24 h of experiments. However, pH 5.5 showed some oscillations due to a lower buffering capacity than solutions at pH 7.0 and 8.5, as previously explained. The greater variability observed at pH 5.5 may be attributed to changes in the ionization state of the investigated SASs, particularly FAV and REM, whose speciation is pH-dependent. These changes, together with alterations in the surface charge of activated sludge flocs, can modify pharmaceutical bioavailability, adsorption to the biomass, and subsequent biodegradation efficiency.

DO levels recorded in controls (5–9 mg/L) showed a trend of higher DO at lower MLSS, while higher MLSS resulted in lower DO levels. This trend is also linked to higher temperatures, at which DO was generally detected at lower levels. At higher temperatures in experiments T10–T27, a decrease in DO concentration after 24 h was observed at lower MLSS values (1–3 g/L), especially for T10, T11, and T19–T21, where a varying group of 5 out of the 6 studied SASs supported this trend. EC measurements for most SAS experiments showed slight changes compared with controls. Some larger oscillations, with increased EC by 8 mS/cm were detected at 45 °C for DCV (T23–T25), and by 2–3 mS/cm for RIT (T23, T25), both at an MLSS of 5 g/L, but also lower values by 1–7 mS/cm for RIT at MLSS of 1–3 g/L (T20–T22, T27), compared with the corresponding controls after 24 h. Considering DO levels for these SASs, there is an indication that biodegradation was absent for DCV (group B, Section 3.3.2), as DO concentration was higher in T23–T25 at an MLSS of 5 g/L, while for RIT (group A, Section 3.3.1) there was a strong indication of biodegradation because of the lower DO levels (T23: 3.35 ± 0.17 mg/L; C: 4.18 ± 0.22 mg/L) and the higher EC supporting mineralization. For RIT at 3 g/L, lower EC and lower DO (T27: 2.30 ± 0.38 mg/L; C: 4.14 ± 0.22 mg/L) at 45 °C were observed, which, to our knowledge, could indicate higher metabolic activity of the microorganisms. At an MLSS of 1 g/L, there was no change in DO levels (T22: 5.47 ± 0.24 mg/L; C: 5.74 ± 0.26 mg/L), but EC decreased, which suggests microbial metabolism was present; however, biomass was too low to affect DO levels at 45 °C for this SAS.

### 3.5. Determination of Full Factorial SAS Removal and Toxicity Percentages

**Figure 5:**
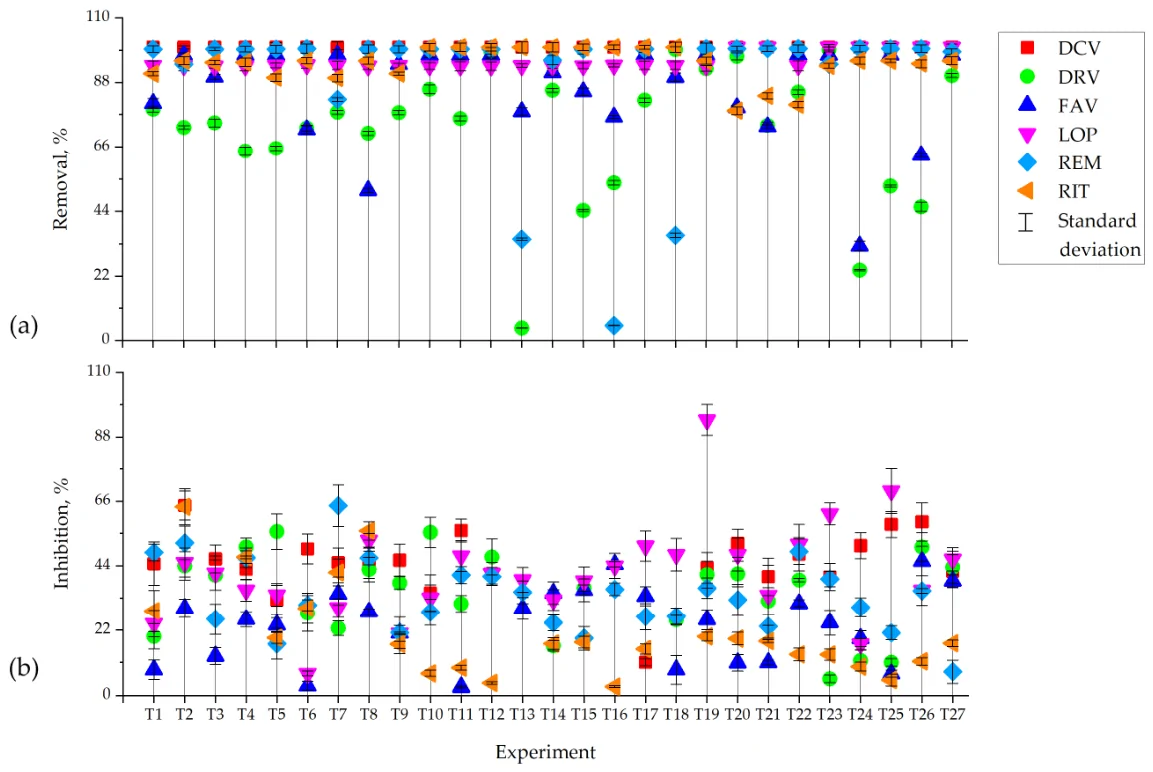
(**a**) SAS removal by bioremediation divided by conducted experiments (T1–T27); (**b**) corresponding inhibition to *Aliivibrio fischeri*. Values represent mean ± SD (*n* = 3).

Using the full factorial optimization plan determined in Design Expert, drop-line scatter plots were created with the coded experiment runs (T1–T27) from Table 3, Section 2.5. The removal results were expressed as percentages (Figure 5a), as was their toxicity to *Aliivibrio fischeri* (Figure 5b). This method of presenting results provides a clear overview of all data from the experiments conducted with three factors at three levels. Detailed values and all raw data are available in Table A3, Table A4, Table A5 and Table A6. The drop-line plots in Figure 5 combine both the removal of each SAS and the corresponding *INH* detected in the samples after 24 h. Overall, removal percentages were consistently high (>90%) under all conditions, except for DRV, FAV, and REM (group A), which showed deviations throughout the experiments. This behavior correlates with the kinetics, biomass stability, and abiotic recoveries described in Section 3.3, suggesting removal efficiency is strongly affected by changes in pH, MLSS, and temperature. DRV and REM showed elevated MLVSS levels, while FAV showed an inhibitory effect, which should also be considered. Correlated removal kinetics and abiotic controls indicated partial adsorption properties at central conditions (0, 0, 0) for DRV (48.25 ± 0.99%), FAV (20.48 ± 0.43%), and REM (41.82 ± 6.19%) at the 24 h, which is allegedly one reason for lower removal percentages under particular conditions compared with other SASs with dominant adsorption and lower abiotic recoveries (group B, <10%).

Toxicity results were calculated according to Equation (6), Section 2.9, and expressed as *INH* for the drop-line plot, as not all samples reached the *EC*_20_ and *EC*_50_ thresholds, allowing comparison across all experiments (Table A4, Table A5 and Table A6). Initial observations indicate little correlation with the removal percentages in Figure 5a, suggesting that, even when SAS removal is high, the residual toxicity may be associated with the possible formation of biotransformation products or other toxic intermediates. Specifically, the lowest toxicity levels were observed in T14 and the highest in T19, highlighting consistently high toxicity associated with LOP. Notably, under these conditions, removal percentages ranged from 85–99% which strongly suggests that bioremediation may result in the formation of toxic SAS biotransformation or biodegradation products. However, as transformation products were not identified or quantified in the present study, this interpretation remains indirect and requires confirmation by future targeted chemical analyses.

Overall, the obtained results demonstrate that high removal efficiencies of the investigated SASs do not necessarily correspond to reduced ecotoxicity of the treated samples. Although removal efficiencies exceeded 90% for most antivirals under most experimental conditions, substantial residual toxicity towards *Aliivibrio fischeri* was still observed. For example, LOP removal consistently exceeded 90%, while luminescence inhibition reached 93.85 ± 5.26% (T19). Similarly, REM and RIT were removed at efficiencies above 90%, whereas the corresponding inhibition remained as high as 64.50 ± 7.10% and 64.09 ± 6.15%, respectively. Comparable discrepancies were also observed for DRV and FAV, where efficient removal was not consistently accompanied by a reduction in toxicity. These findings indicate that the disappearance of the parent compounds from the aqueous phase cannot be regarded as evidence of complete detoxification. Instead, the observed toxicity profiles are consistent with the formation of biologically active transformation products during microbial biotransformation. Previous studies [33,34,35] have shown that pharmaceutical biotransformation frequently produces transformation products that retain biological activity or even exhibit greater persistence and toxicity than the parent compounds, emphasizing that chemical removal alone cannot be considered a reliable indicator of environmental safety. Escher and Fenner [36] further highlighted that when toxicity does not decrease in parallel with the disappearance of the parent compound, transformation products should be considered environmentally relevant and included in environmental risk assessment. Therefore, the present results underline the importance of combining chemical analyses with ecotoxicological assays when evaluating biological wastewater treatment processes, as removal efficiency alone may substantially overestimate the environmental safety of treated effluents.

#### 3.5.1. Daclatasvir (DCV)

Results for DCV showed consistently high removal efficiency, with removal exceeding 99% under all experimental conditions. Considering the variable toxicity levels for each factor and level, DCV removal is clearly more complex than it appears. Although initial indications suggest a strong correlation with adsorption of DCV to activated sludge, toxicity results indicate that active biodegradation or biotransformation also occurs in this process. Since the initial toxicity of DCV is 25.87 ± 0.36%, most results showed elevated *INH* levels (30–65%), except for T17 (A: 0, B: −1, C: 0), where toxicity was 11.09 ± 1.16%, and T12–T16, and T18 which had no negative effect and were all conducted at 30 °C. Because DCV removal was at the same level under all conditions, this SAS was not included in the determination of optimal conditions, as RSM could not be applied.

#### 3.5.2. Darunavir (DRV)

DRV showed very low removal (<50%) in T13 (A: 1, B: 0, C: 0), T15 (A: 0, B: 1, C: 0), T24 (A: 1, B: 1, C: 1), and T26 (A: 1, B: 0, C: 1). These conditions all shared pH values of 7 or 8.5, temperatures of 30 or 45 °C, and MLSS values of 3 or 5 g/L. The lowest removal, 4.19 ± 0.03%, was detected in T13, and 24.09 ± 0.23% in T24. This suggests that higher MLSS values and higher pH values (7–8.5) negatively affect DRV degradation. Most results for this SAS are in the range of 50–90%, but consistently high removal (>95%) was observed in T12 (A: −1, B: 1, C: 0), T18 (A: 1, B: −1, C: 0), T20 (A: 0, B: −1, C: 1), and T23 (A: 0, B: 1, C: 1). This suggests that DRV removal from the solution is influenced by pH, MLSS and temperature control in the experiments conducted. Removal efficiency of DRV is partially correlated with detected lower toxicity, especially for T18 (*INH* = 25.48 ± 1.45%) and T23 (*INH* = 5.52 ± 1.28%), where DRV was removed in high concentrations, and also for T24 (*INH* = 11.67 ± 3.52%), which had lower DRV removal rate as previously discussed. An interesting observation was made for T13, T16, and T17, which showed no toxicity to the bacteria even though the removal percentages were very different (4.19 ± 0.03%, 53.64 ± 0.73%, and 81.98 ± 0.79%, respectively); otherwise, most samples showed elevated toxicity levels of 30–60% relative to the initial toxicity of DRV (*INH* = 35.36 ± 1.44%), suggesting the formation of degradation products. Overall, lower toxicity (10–20%) for DRV was achieved in experiments T1 (A: 1, B: 0, C: −1), T14 (A: 0, B: 0, C: 0), T24 (A: 1, B: 1, C: 1), and T25 (A: −1, B: 1, C: 1), with the only correlation of the tested factors being higher MLSS values of 3–5 g/L.

#### 3.5.3. Favipiravir (FAV)

In the experiments with FAV, a total of nine deviations (30–85%) were observed in the drop-line plot in Figure 5a. These include experiments T1, T3, T13, T15–T16, T20–T21, T24, and T26, which showed a negative trend with increasing MLSS in the range of 3 to 5 g/L across all levels of pH and temperature. Measured toxicity showed fewer deviations at lower temperatures, 2–35%, which is lower than or equal to the initial FAV toxicity of 31.48 ± 4.55%. At higher temperatures, there is a wider range of deviations, from 2 to 45%. This suggests the presence of potential degradation products with higher toxicity to *Aliivibrio fischeri* than the parent FAV molecule. It is important to note that in experiments T10 (A: −1, B: 0, C: 0) and T12 (A: −1, B: 1, C: 0), there was no negative effect on the bioluminescence of the bacteria, which can be attributed to a removal efficiency of over 97% in these experiments.

#### 3.5.4. Lopinavir (LOP)

LOP removal was highly effective, with all values exceeding 90% for every tested combination in the full factorial design (T1–T27). When compared with toxicity data, most *INH* values were recorded between 15% and 55%. The initial toxicity of LOP to the bioluminescent bacteria was 38.02 ± 0.99%, and LOP was effectively removed from the solution in most cases (Figure 5a). This supports the adsorption-dominant mechanism, but also suggests the appearance of LOP biodegradation products at 45 °C. The highest *INH* was observed in T19 (A: −1, B: 0, C: 1) at 93.85 ± 5.26%, while the lowest toxicity for LOP was found in T6 (A: 1, B: 1, C: −1) at 7.31 ± 0.80%. These results indicate a correlation between toxicity and temperature, as the highest temperature showed the highest level of *INH*, while the lowest temperature showed decreased *INH*. This consistently high toxicity can also be related to the toxic effect of adsorbed LOP on the MLVSS change of biomass shown in Figure 4b, Section 3.2.

#### 3.5.5. Remdesivir (REM)

Another highly effective removal was observed for REM, with percentages above 90% in most experiments. However, there were deviations with lower removal of this SAS at T7 (82.19 ± 0.73%), T13 (34.55 ± 0.41%), T16 (5.04 ± 0.08%), and T18 (35.81 ± 0.79%). This suggests that temperature of 10 and 30 °C were not sufficiently effective for REM biodegradation, especially at pH 8.5. Toxicity showed an increasing trend at lower temperature; at 10 °C in T7, *INH* reached 64.50 ± 7.10%. This is also confirmed by T1–T2, T4, and T8, which had elevated toxicities above 45% compared to other temperatures. These results also suggest the formation of biotransformation and biodegradation products, as the initial toxicity of REM was measured at 53.36 ± 2.01%. Other experiments had lower toxicities in the range of 15–45%, which indicates another source of toxicity besides the effectively removed REM.

#### 3.5.6. Ritonavir (RIT)

RIT removal exceeded 85%, except for T20 (A: 0, B: −1, C: 1), T21 (A: 1, B: −1, C: 1), and T22 (A: −1, B: −1, C: 1), which were in the 75–85% range. All experiments were conducted at 45 °C, with different pH values and the same MLSS of 5 g/L, suggesting that RIT removal depends mainly on the interaction of temperature and MLSS during bioremediation. The initial *INH* of RIT at 25.14 ± 2.89%, while the toxicity of the samples ranged from 2% to 20% at higher temperatures, but from 20% to 65% at 10 °C. This is supported by the observation that the lowest measured *INH* was 2.88 ± 0.32% in T16 (30 °C), and the highest was 64.09 ± 6.15% in T2 (10 °C). There is also a visible trend for toxicity in Figure 5b, indicating higher *INH* levels at lower temperatures. This suggests that higher temperatures are more suitable and effective for partially removing the degradation products formed after bioremediation, although some toxicity remains.

### 3.6. Modeling of Experimental Data and Statistical Analysis

The responses selected for RSM modeling were the removal percentages for each SAS (five responses). DCV was not included in the RSM because removal was equal across all experiments, as described in Section 3.5.1. Experimental data points were treated as mean values, while center points were treated as triplicates. This approach was chosen to minimize noise from biological fluctuations and clarify trends in the removal results. The center points were kept as three separate measurements to stabilize model prediction; however, because these points were very precise (Table A3), ANOVA yielded a low pure error, which directly influenced the Lack of Fit test to be significant (*p* < 0.05), regardless of the model type for all SASs.

To successfully apply RSM to identify the optimal conditions for the removal process, we determined the model order based on the best fit to our results, using the lowest *p*-value, highest *R*^2^, and minimal difference between adjusted and predicted *R*^2^. The observed *R*^2^ values (0.5262 to 0.8624) are considered typical for complex biological and bioremediation processes involving activated sludge, where inherent biomass variability influences the response. The Adequate Precision ratio was also taken into account, and values higher than 4 were considered acceptable, indicating an adequate signal to navigate the design space. These criteria were satisfied for DRV, FAV, LOP, and RIT, as shown in Table 7. In contrast, REM yielded consistently negative predicted *R*^2^ because of the extremely narrow dataset (94–100%). Therefore, the overall mean was chosen as the best predictor for this SAS (REM removal: 98.07 ± 3.47%).

#### 3.6.1. Adequacy Check and Model Refinement

In more detail, initial model assumptions were quadratic for these SAS responses. However, when screened for outliers using the iterative removal procedure described in Section 2.5, two outlying data points were excluded for DRV (T17, T24), and two for FAV (T2, T27). These observations were classified as outliers because they consistently exceeded the predefined diagnostic criteria and were associated with replicate variability exceeding 15%, which is likely attributable to the intrinsic heterogeneity of the activated sludge inoculum and transient fluctuations in microbial activity during sampling. Their exclusion followed the predefined statistical protocol and was performed to ensure model validity rather than to artificially improve model fit. T17 exceeded the externally studentized residuals limit for DRV, while T21 and T24 were identified as influential points beyond the DFFITS limits of the model predictions. T24 was removed together with T17 to obtain a significant DRV model, and T21 was retained to keep the model predictions as close as possible to the genuine biological response. For FAV, T2 was also detected beyond the externally studentized residuals limit, while T27 was the most outlying data point in the DFFITS test and therefore was also excluded to obtain a significant model.

Although these models proved significant, further changes were made to a reduced 2FI model for DRV and reduced quadratic model for FAV by additionally removing of insignificant factors with the highest *p*-values (DRV: AB, A^2^, B^2^, C^2^; FAV: B^2^) after outlier removal, to provide adequate model fit and an acceptable difference between adjusted and predicted *R*^2^ (<0.2). Final model equations are presented in Table 7. All models had *p*-values below 0.05 and *F*-values ranging from 4.66 to 13.23 (Table A10), indicating that the models are significant and that there is only a 0.01–0.51% chance that an F-value this large could occur due to noise.

**Figure A2:**
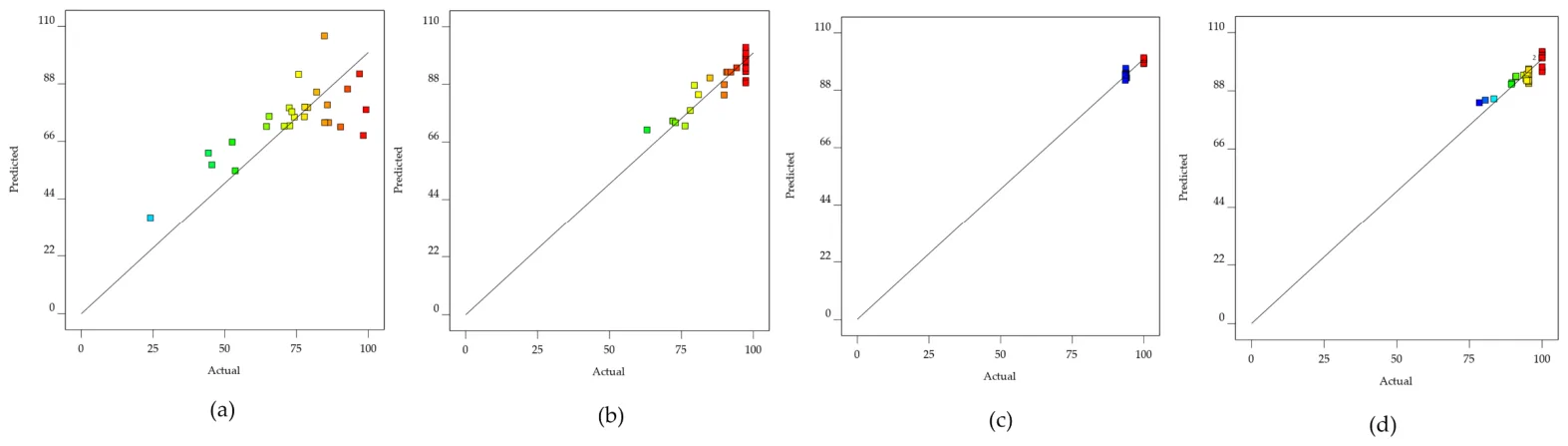
Comparison between actual experimental data and model-predicted values for (**a**) DRV (blue: 24.09%–red: 99.21%), (**b**) FAV (blue: 63.06%–red: 97.35%), (**c**) LOP (blue: 93.55%–red: 100%), and (**d**) RIT (blue: 78.41%–red: 100%). The number “2” on the plot indicates two coincident data points (overlapping values) at that coordinate.

Further diagnostic tests on the models are also described in Section 2.5. Approximate normality and random dispersion patterns were verified for DRV, FAV, and RIT by normality plots and residuals-versus-predicted plots. The assumption of homogeneity was not met only for the LOP model. Although the model proved significant and in good agreement with all criteria, the residuals-versus-predicted plot showed an interesting trend, which can also be seen in the predicted-versus-actual plot in Figure A2. As the dataset was too narrow for modeling of REM response, a similar situation is present for LOP (Figure A2c), but with two distinct values, around 94% and 100%. If this is connected to the low abiotic recovery of LOP, reaching 6–7% on average (Section 3.3, Table 6), there is a direct correlation to conditions in which even 6% of available LOP in the solution was biodegraded by microorganisms, while in the other case LOP was specifically only adsorbed onto the surface. Another observation is that 100% removal was achieved specifically at a higher temperature of 45 °C.

#### 3.6.2. Analysis of Factor Interactions

**Figure 6:**
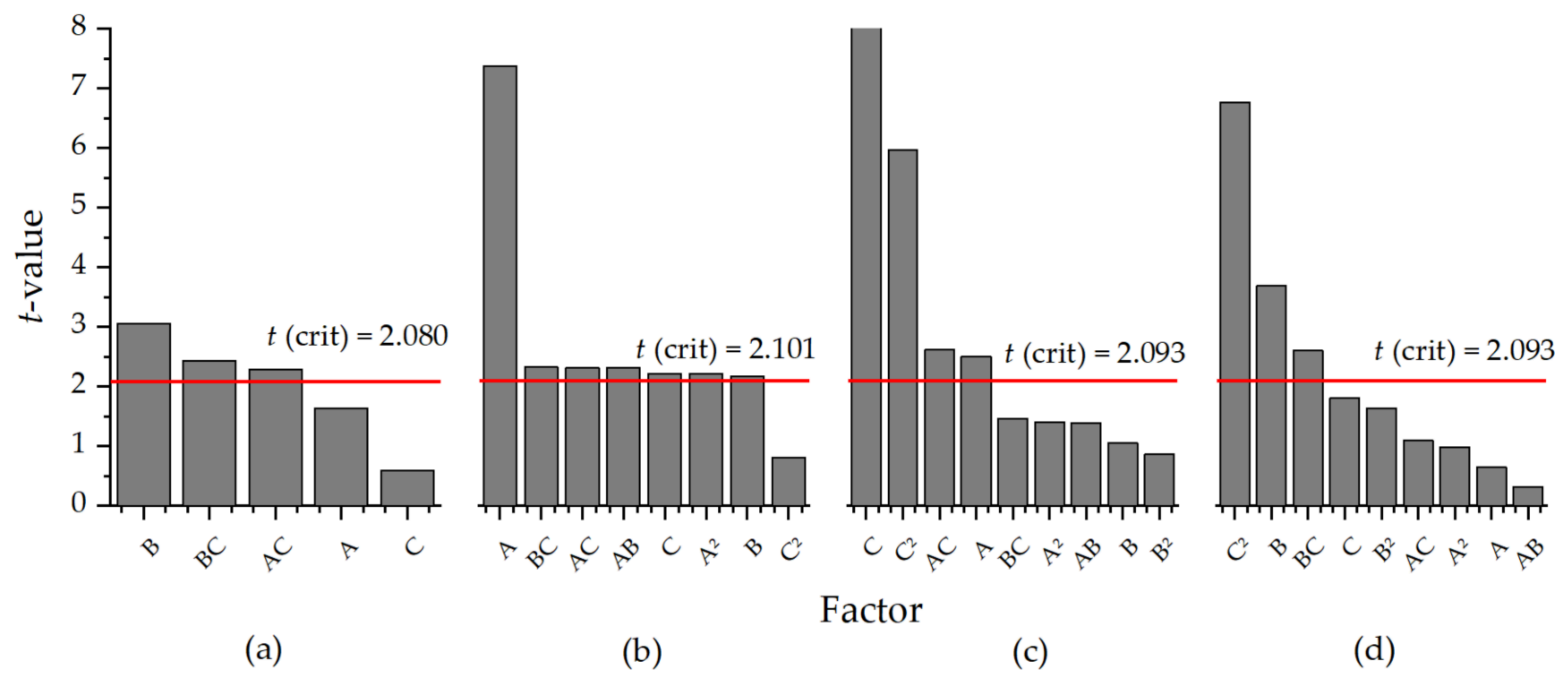
Pareto charts of standardized effects (*t*-test, *α* = 0.05) for: (**a**) DRV (*df* = 21), (**b**) FAV (*df* = 18), (**c**) LOP (*df* = 19), and (**d**) RIT (*df* = 19).

Significant model terms (*p* < 0.05) and insignificant terms (*p* > 0.05) were identified for successfully modeled SAS responses. To provide a clearer visualization of factor importance, the Pareto chart in Figure 6 shows the standardized effects, calculated as the absolute *t*-values (√F, *df* = 1) from the ANOVA data (Table A10). The vertical red line represents the threshold of critical *t*-values (*α* = 0.05) with the corresponding degrees of freedom for each model, indicating that any factor or interaction with a bar above the line is statistically significant.

The most frequently detected significant model terms were B and BC for DRV, FAV, and RIT, representing MLSS and the MLSS-*T* interaction. These SASs correspond to group A, which is dominated by biodegradation over adsorption (Section 3.3.1). Higher MLSS and an increased MLSS-*T* interaction are expected to support biodegradation by microorganisms, as their abundance and metabolic activity are higher, which agrees with our previous observations. Furthermore, with higher temperature, adsorption strength is also expected to decline because of higher molecular kinetic energy. DRV also included the interaction term AC (pH-*T*) as significant, while for RIT, C^2^ (*T-T*) proved to be by far the most significant interaction in the *t*-test. These observations further support our speculations about the inevitable influence of temperature on overall removal performance.

For FAV, additional significant model terms were A, C, AB, AC, and A^2^, representing pH and temperature, their interaction, the pH-MLSS interaction, and the pH-pH interaction. The highest *t*-value was calculated for factor A, suggesting that pH had a strong influence on the model prediction values and, most importantly, on the ionisation state of FAV. To our knowledge, the FAV molecule is in a neutral state at lower pH, possibly a more favourable state for microbial digestion and diffusion through cell membranes, whereas elevating pH levels changes the molecule to the ionized state and contributes to higher surface charge and repulsion. Additionally, we observed that FAV had the highest number of significant terms.

The four significant model terms for LOP were A, C, AC, and C^2^, indicating a strong influence of pH and temperature changes on LOP removal. The highest significance was detected for C and C^2^, while interactions with factor A appeared less significant for the model predictions of removal. When connected with previous speculations on an adsorption dominant process in LOP removal (group B), these interactions can be interpreted from another angle. An increase in temperature had a strong influence on the desorption of LOP, as previously described, particularly at 45 °C. It is assumed that at this temperature, adsorption bonds weakened enough to release a small amount of LOP, making it available for biodegradation. LOP is generally known to be a neutral molecule with changing pH; however, only at 45 °C was there a significant influence of pH, demonstrating that these interactions were more relevant for changes in the physicochemical properties of the adsorbent, activated sludge, than for the targeted molecule LOP.

### 3.7. Determination of Optimal Conditions with Response Surface Methodology

**Figure A3:**
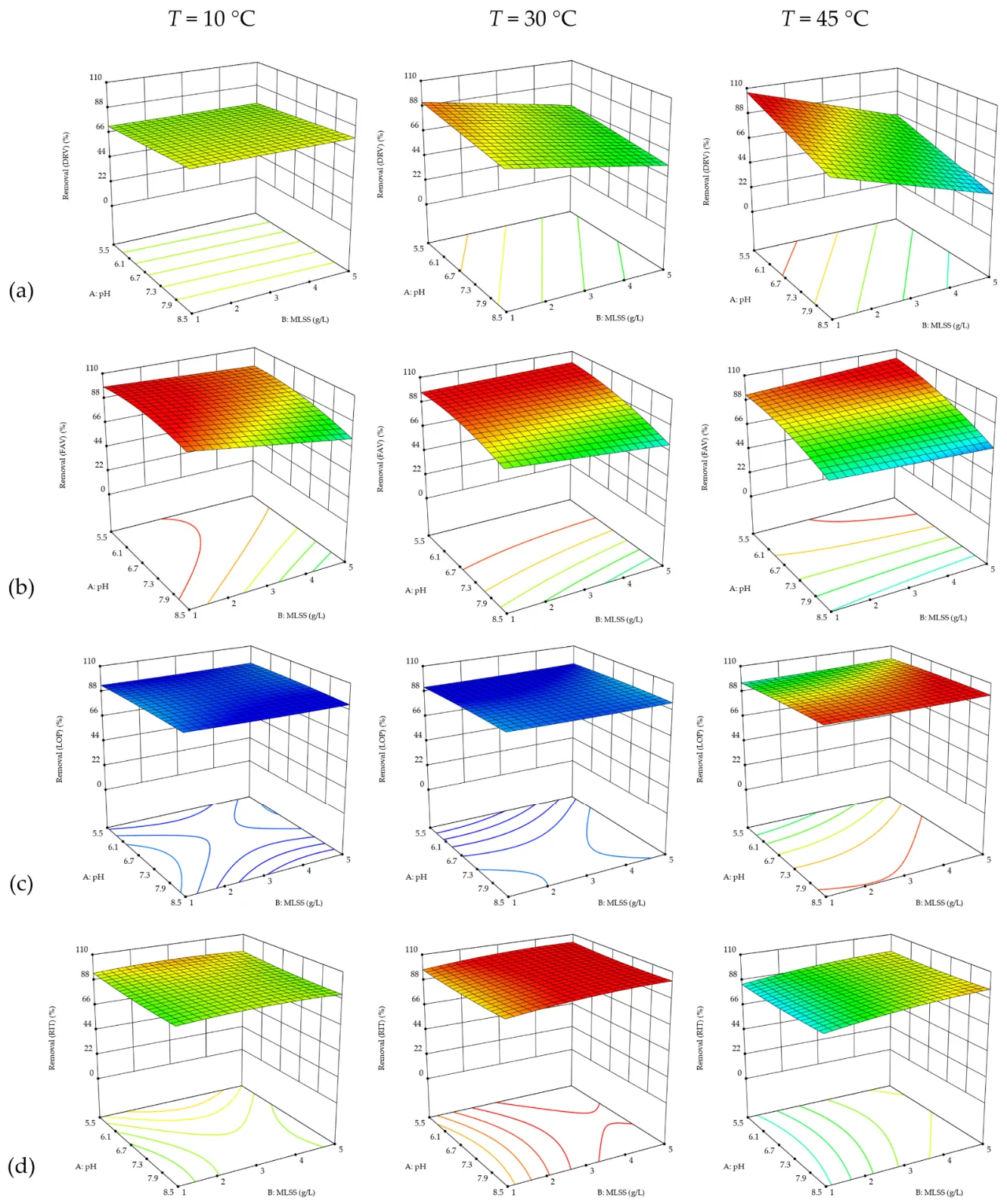
Response surface methodology (RSM) for the degradation of SASs with full factorial design (3^3^). 3D surfaces for each level of factor C (*T* = 10, 30, and 45 °C) for: (**a**) DRV (blue: 24.09%–red: 99.21%), (**b**) FAV (blue: 63.06%–red: 97.35%), (**c**) LOP (blue: 93.55%–red: 100%), and (**d**) RIT (blue: 78.41%–red: 100%).

To estimate the optimal conditions, 3D surfaces were generated using RSM and the models described in Section 3.6, incorporating each combination from the full factorial (3^3^) design (T1–T27). Individual 3D surfaces for DRV, FAV, LOP, and RIT removal are shown in Figure A3. A crucial part of the optimization was defining the goal for each parameter and response. Factors A, B, and C were set to ranges within the experimental window (–1 to 1), while responses were targeted at their maximum removal percentages. Design Expert 12 software calculated 35 solutions, of which the first 11 shared a desirability of 0.772, with values of pH = 6.08 ± 0.02, MLSS = 4.41 ± 0.08 g/L, and *T* = 44.99 ± 0.03 °C. Under these conditions, the removal percentages of SASs were: DRV (66.08 ± 1.05%), FAV (97.35 ± 0.01%), LOP (98.50 ± 0.10%), REM (98.08 ± 0.00%) and RIT (93.45 ± 0.08%). The mean values were ultimately chosen as the optimal conditions for the SAS removal process. These optimal conditions were defined by interpolation of our experimental data at 45 °C, between pH 5.5 and 7.0 and MLSS of 3 and 5 g/L (T19, T23, T25 and T27). Figure 5 shows that these design points had significantly high removal; however, in T19 there was an extreme rise in toxicity detected for LOP, and no noticeable decline in the *INH* effect on *Aliivibrio fischeri*. Therefore, the temperature of 44.99 °C, which was determined to be optimal, did not coincide with the lowest observed toxicity (T14, Section 3.3), which occurred at 30 °C. Nonetheless, 45 °C is still considered the most effective temperature to remove SASs from the aqueous phase and, even though a trade-off exists with the observed toxicity, we do not claim that biodegradation is sufficient to completely solve this particular problem. Further research is needed, with advanced biological or oxidation processes as a second phase after biodegradation, to determine whether complete ecotoxicological safety can be achieved.

**Figure 7:**
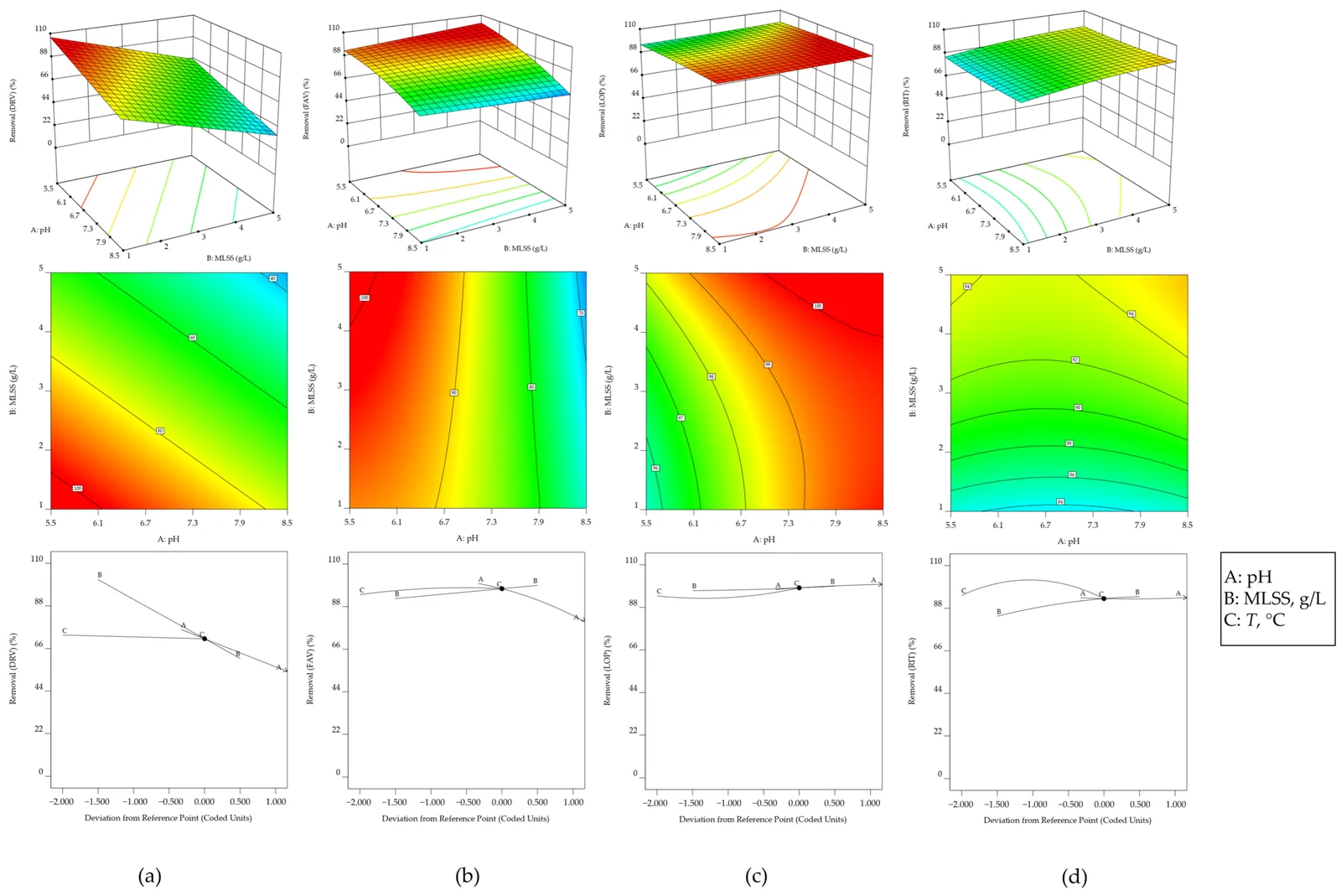
Response surface methodology (RSM) and optimization plots using full factorial design (3^3^) for the degradation of SASs. The 3D surfaces, contour, and perturbation plots at determined optimal temperature (*T* = 44.99 °C) are shown for: (**a**) DRV (blue: 24.09%–red: 99.21%), (**b**) FAV (blue: 63.06%–red: 97.35%), (**c**) LOP (blue: 93.55%–red: 100%), and (**d**) RIT (blue: 78.41%–red: 100%). The continuation of the perturbation factor interactions is indicated visually by arrows.

Figure 7 presents the RSM models as 3D surface, contour, and perturbation plots at the optimal fixed temperature of 44.99 °C for easier comparison between SASs. The 3D surfaces show the overall contribution of pH and MLSS to the removal process, while the contour plots display the gradient from the lowest removal (blue) to the highest (red), with an individual scale for each SAS. The prediction flags indicate the conditions at which the highest removal and desirability (0.772) were achieved. The perturbation plots in Figure 7 illustrate the sensitivity and relationship between the individual factors A: pH, B: MLSS, and C: *T* as steep curves or horizontal profiles in relation to the removal percentages. The reference point (0) was taken as the previously mentioned optimal temperature (*T* = 44.99 °C). While all factors exert some level of influence on the individual response removals, their varying slopes visually confirm the ANOVA findings; the steepest curves identify the dominant parameters, whereas the gentler slopes indicate factors with a minor effect on the process. Notably, Figure 7c shows the LOP perturbation plot, where factor C shows a a minimum at around room temperature (22 °C). This is an unusual observation compared with other SAS removal trends.

**Figure 8:**
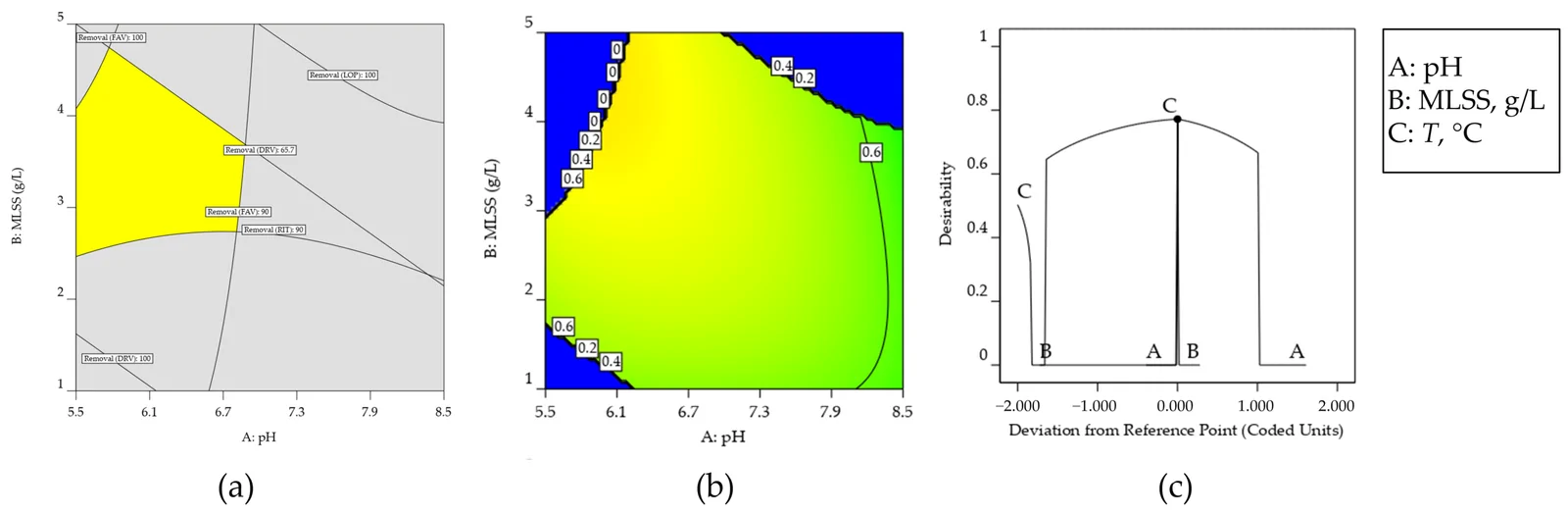
Evaluation of factor interactions, their significance, and desirability: (**a**) overlay plot with limiting factors (DRV 66%, FAV 90%, and RIT 90%); (**b**) desirability contour plot; (**c**) desirability perturbation plot with factor A, B, and C interaction profiles.

For further analysis of the selected optimal conditions, Figure 8 presents the overlay, desirability contour, and perturbation plots. Figure 8a shows the SAS removal overlay plot, fixed at the optimal temperature of 44.99 °C. The yellow area represents the desired range of optimal conditions, while the gray area indicates limitations. The limiting factor was identified as DRV, with removal at 66%, due to drastically lower process removal capabilities and 2FI model interactive effects, while other limitations were set at removals higher than 90%, as per the observed effectiveness and the determined quadratic models. The plot shows that FAV and RIT also had an additional limiting influence on the selection of optimal values. Comparing this plot to Figure 8b, which illustrates the relationship between pH, MLSS, and desirability, we observed a connection to the optimal yellow area. The blue area of the plot indicates a non-desirable combination of factors A and B (desirability = 0). The highest point was detected in the upper yellow area at 0.772, with a pH of 6.08 ± 0.02 and MLSS of 4.41 ± 0.08 g/L. Figure 8c shows the perturbation plot demonstrating the sensitivity of factors A, B, and C to overall desirability. All factors exhibited specific thresholds and abrupt changes in desirability. Factors A and C showed similar influence, rising from 0 to their maximum desirability of 0.772 at 45 °C, followed by a downward slope for factor A and factor C reaching the design endpoint. Factor B reached the same maximal desirability but with the opposite trend, dropping to 0 immediately after the optimal conditions point. These pronounced changes indicate that the process is highly sensitive to variations in the operational parameters investigated, particularly at pH and temperature values below the identified optimum, whereas MLSS values exceeding the optimum substantially reduced the overall desirability of the process. The pronounced effect of temperature observed in the perturbation plots further suggests that thermal conditions governed the overall biological performance of the activated sludge system to a greater extent than biomass concentration alone. Within the investigated range, increasing temperature likely enhanced microbial metabolic activity and the catalytic efficiency of enzymes involved in the biotransformation of SAS, while simultaneously improving mass transfer of the target compounds through the extracellular polymeric substances matrix toward metabolically active microorganisms. Consequently, temperature influenced both the bioavailability of the investigated antivirals and the kinetics of their enzymatic degradation, explaining its dominant contribution to the developed RSM models. Similar observations have been reported for activated sludge systems treating pharmaceutical contaminants, where temperature was identified as one of the principal factors governing microbial metabolism, enzymatic transformation, and substrate transport within microbial flocs [37]. Furthermore, this specific optimal configuration was established as the reference point (coded 0) for this plot, as well as for the individual response perturbation plots previously presented in Figure 7.

## 4. Discussion

### 4.1. Common Microorganisms in the Activated Sludge Monitoring

In wastewater treatment for emerging micro-pollutants from the pharmaceutical industry, one biological process is deeply integrated into communal WWTPs: the activated sludge process [38]. This process involves aeration of mixed microbial communities and oxidation of organic contaminants, producing new cells, carbon dioxide, and water. It increases biomass concentration and enhances the removal of sludge flocs from the effluent by gravity settling [39]. Activated sludge primarily consists 90–95% bacteria, which form the physical structure of the flocs and carry out biodegradation process [40]. According to Microscope World, microfauna make up about 5% of the total biomass, including 4% protozoa and 1% metazoa. Common protozoa in wastewater are ciliates, flagellates, and amoebae, which clarify the effluent by consuming free-swimming bacteria [41]. Czapluk et al. found that the dominant groups in activated sludge were attached ciliates (42%), naked amoebae (10%), and testate amoebae (7%). The most frequently detected species were *Litonotus lamella*, *Trachelophyllum pusillum*, *Vorticella alba*, *Vorticella convallaria*, *Vorticella microstoma*, *Vorticella striata*, *Arcella vulgaris*, *Cochlipodium granulatum*, *Euglypha tuberculata*, *Pyxidicula* sp., *Aspidisca cicada*, and *Epistylis coronata* [42]. Metazoa such as rotifers, nematodes, bristle worms, and tardigrades are commonly found in mature systems with higher DO concentrations [41]. Czapluk et al. also recorded flagellates, rotifers, nematodes, and tardigrades in the investigated WWTP [42]. In our study, activated sludge contained large numbers of protozoa, including stalked ciliates similar to *Vorticella* species and *Epistylis coronata*, as well as testate amoebae such as *Euglypha tuberculata,* among others. Metazoa were present in lower numbers and were mostly detected as rotifers and tardigrades. Czapluk et al. also noted the well-known variability of microfauna in activated sludge throughout the year [42], which explains our higher SD values in activated sludge characterization (Table 5). Microorganisms account for about 60–80% (volatile solids) of the dry matter [23] of activated sludge, which is around 0.6–6.0% in conventional WWTPs [43]. The corresponding water content is usually between 95 and 99% [44], which was confirmed by our results: water content of 99.57 ± 0.13% and a dry matter content of 0.43 ± 0.13%. Volatile solids were 64.29 ± 6.09%, which was within the desirable range.

The observed seasonal variability of the activated sludge microbial community has important implications for the robustness and long-term stability of biological wastewater treatment processes. Seasonal fluctuations in temperature, influent composition, hydraulic loading, and nutrient availability continuously influence the abundance and activity of key microbial populations responsible for pharmaceutical biodegradation. Consequently, the efficiency of contaminant removal may vary throughout the year, particularly for emerging contaminants whose degradation depends on specialized microbial consortia. These observations highlight the importance of maintaining a diverse and resilient microbial community capable of adapting to changing environmental conditions, thereby ensuring stable treatment performance under full-scale wastewater treatment plant operation.

### 4.2. Operational Parameters in the Activated Sludge Process

#### 4.2.1. Control of DO

The efficiency of biodegradation in the activated sludge process is determined by operational parameters such as DO, temperature, and pH [45]. DO concentration is critical, as levels below 1.1 mg/L significantly reduce the settling properties of activated sludge and may cause filamentous bulking or low treatment efficiency [46]. In our research, the initial DO of activated sludge (Table 5) was 3.30 ± 1.17 mg/L, slightly above the optimal range of 1.0–3.0 mg/L [47]. Concentrations in the experiments were measured in smaller volumes of 150 mL, with higher DO levels between 5 and 10 mg/L. This is beneficial for biodegradation, as values above 4.0 mg/L help oxygen penetrate the flocs [48], promoting enhanced metabolism in aerobic bacteria in the activated sludge [49].

#### 4.2.2. Control of Temperature

Temeprature is also a crucial factor, as it is directly linked to the solubility of oxygen in water [50]. Meynet et al. found that at 20 °C, the microbial community is highly biologically diverse, while below 15 °C and above 25 °C, less common and more specific bacteria prevail. Based on this, temperature is considered an important parameter for microbial composition and for the metabolic pathways responsible for the degradation of pharmaceuticals [37]. The measured initial temperature of the activated sludge sample was room temperature, 22.77 ± 1.17 °C, and the experiments were conducted at 10, 30, and 45 °C, which was an excellent range for testing the efficiency of metabolic pathways in the removal of SASs.

#### 4.2.3. Control of pH

The optimal pH for treatment with activated sludge is between 6.5 and 8.5, as researched in detail by Kokina et al., who investigated rapid changes at the borderline pH values of 6.5 and 8.5 on the nitrification process. They found that acidic pH had a greater impact than alkaline pH on the microbial population, specifically by inhibiting the first step of nitrification at pH 6.5, while at pH 8.5 there was a lower potential for inhibition of the second step of nitrification [51]. The measured pH of the initial activated sludge sample was 7.34 ± 0.51, which was within the optimal range. The optimization experiments were conducted at pH 5.5, 7.0, and 8.5, in accordance with the described research, to test the influence of pH on the SASs removal process.

#### 4.2.4. Control of EC

Another important parameter to consider is the EC of the treatment process, as it determines the amount of dissolved substances, chemicals, and minerals in the water [52]. This is essential for assessing microbial activity and health, as stable supernatant EC levels indicate a balanced ecosystem, while fluctuations suggest external toxicity or operational issues [53]. According to Agnieszka et al., the optimal range for EC is 0.60–0.80 mS/cm [54], which was confirmed by the initial EC of 0.87 ± 0.16 mS/cm. In T1–T27 measurements, EC values were notably higher, ranging from 8 to 22 mS/cm, as the experiments were prepared in the mineral medium described in Section 2.2. According to Guo et al., the EC of phosphate buffer at 0.1 M concentration is 18.3 mS/cm at pH 6.8–7.0 [55], a higher value than our measured EC in the controls, which was around 12 mS/cm (pH 7.0) when mixed with activated sludge. This observation was confirmed by Kamiyama et al., who studied the recovery of K^+^, Mg^2+^, NH_4_^+^, and PO_4_^3−^ from activated sludge. Their study demonstrated that a large quantity of these ions rapidly binds through biosorption and ion exchange, reducing their concentrations in the solution [56] (in our case, the mineral medium). These findings align with the measured EC values, especially as the biomass concentration increased from 1 g/L to 5 g/L. This can be attributed to the higher density and number of active sites on the floc surfaces, leading to a reduction of free ions in the solution [57] and decreasing the EC of controls from 18 mS/cm (1 g/L) to 9 mS/cm (5 g/L).

### 4.3. Main Factors Influencing SASs Removal Efficiency

#### 4.3.1. Effects of Elevated SAS Concetrations

Pharmaceutical removal has been shown to depend on three main factors: (1) pollutant type and concentration, (2) hydraulic and solids retention time (HRT, SRT), and (3) temperature [38]. Slater et al. investigated the effects of pandemic-level dosing of SASs, specifically oseltamivir, on wastewater treatment and demonstrated that elevated concentrations can destabilize WWTP microbial consortia [58]. Accordingly, in our experiments, SASs removal efficiency was in strongly correlation with toxicity assays on *Aliivibrio fischeri* [59] and microbial destabilization was attributed to the negative effects of either parent SASs or their transformation products [60].

#### 4.3.2. Effects of HRT and SRT (MLSS)

The second factor, HRT, refers to the time wastewater remains in the reactor and typically ranges from 4 to 24 h in the activated sludge process, depending on the desired level of treatment [61]. Therefore, the bioremediation study was conducted over 24 h to test the maximum realistic HRT for this process. Closely related to this factor is SRT, which defines microbial growth rate and composition and is directly proportional to biomass concentration, resulting in increased MLSS [23]. Hatoum et al. investigated the relationship between HRT, SRT, and MLSS in the removal of various pharmaceuticals and reported enhanced removal with an increase in MLSS from 3 to 5 g/L, while an MLSS of 8 g/L showed a lower removal rate than expected [62]. Our results confirmed this trend, although the optimal MLSS was determined to be 3.89 ± 0.03 g/L, indicating a shift in the maximum to 4 g/L instead of 5 g/L, which is attributable to the evaluation of different compounds (SASs). A reduction of MLSS during the biodegradation process can indicate pharmaceutical-induced alterations in biofilm structure, metabolic function, composition, and bacterial proliferation [63]. These alterations can be associated with the presence of DRV, FAV, and RIT relative to the corresponding central point (0, 0, 0) controls (Figure 4b).

#### 4.3.3. Effects of MLVSS

The organic microbial fraction of activated sludge was evaluated using MLVSS, expressed as a percentage, which typically equals 60–80% of overall MLSS in conventional systems [29]. A decline in MLVSS, observed for FAV and LOP at the central point (0, 0, 0) (Figure 4b), can indicate a negative effect on microbial community structure, as described by Stavrakakis et al. in research on m-chlorophenol [64]. Conversely, an increase in MLVSS directly indicates suitable conditions for bacterial proliferation with primary nutrients in the medium, facilitating cometabolic biodegradation. This trend is described by Lee et al., who highlight the difference between bacterial strains that biodegrade micropollutants directly and those that use different enzymes and only mineralize the by-products into CO_2_ [65]. DRV and REM demonstrated this effect by showing a 10–20% increase in MLVSS compared to controls at central point conditions (0, 0, 0) (Figure 4b), providing clear evidence of high bacterial activity and active replication in the removal of these SASs. Alobaidi et al. found that maintaining a constant MLVSS over 24 h further supports good process performance and balanced maintenance metabolism with slowly growing bacteria [66], as observed with DCV and RIT. However, this can also indicate adsorption instead of biodegradation and requires further investigation for emerging pharmaceuticals in wastewater, as was also suggested.

#### 4.3.4. Effects of Temperature

The last, but very important key factor is temperature. Zembrzuska et al. [67] investigated the effect of temperature on ibuprofen biodegradation in activated sludge and demonstrated that pharmaceutical removal increased with temperature, with significantly faster degradation observed at 8, 18, and 28 °C. Similarly, LaPara et al. [68] investigated pharmaceutical wastewater treatment and reported that process performance declined at 55 °C, whereas more efficient pharmaceutical removal was achieved at 30 °C. In our experiments, optimal conditions for SASs removal were determined at 44.99 ± 0.03 °C, confirming literature data that lower temperatures (10 °C) were less effective and that higher temperatures (>45 °C) caused thermal stress and created an inhibitory environment for activated sludge bacteria, resulting in lower SASs removal efficiency [37].

#### 4.3.5. Effects of pH

An additional parameter influencing removal kinetics is pH, which was determined to have an optimal value of 5.90 ± 0.02. Gulde et al. found that biotransformation is pH-dependent and correlates qualitatively with the neutral fraction of ionizable pharmaceuticals [60]. The studied SASs were mostly deprotonated at pH 6, especially DRV (p*K*_a_ = 2.39 (basic)/13.59 (acidic) [69]) and REM (p*K*_a_ = 0.65 (basic)/10.23 (acidic), which are amphoteric, followed by LOP (p*K*_a_ = 13.39 (acidic)/−1.5 (basic)), which is non-ionizable, and RIT (p*K*_a_ = 2.84 (basic) [70]) which is a very weak base. DCV (p*K*_a_ = 6.09 [71]) is also a weak base and is expected to be 50:50 protonated/deprotonated at pH 6. Interestingly, FAV (p*K*_a_ = 5.10 (acidic) [72]) is a weak acid and exists as a negatively charged anion at pH 6, which was studied by Timur et al. [73] and Eryildiz-Yesir et al. [74] as the most bioaccessible and most soluble form of FAV.

### 4.4. Mechanisms Describing SASs Removal Kinetics

**Figure 9:**
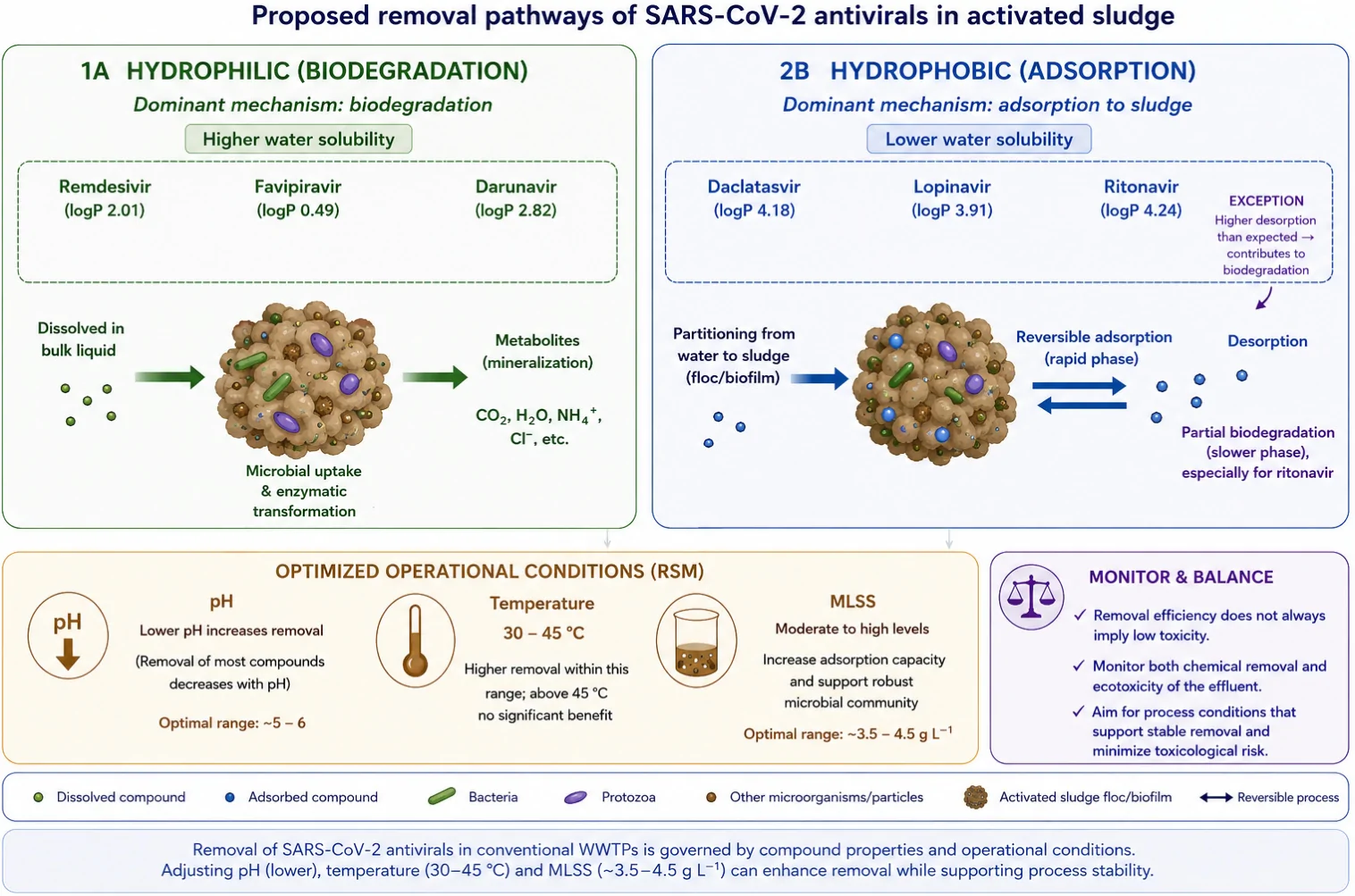
Removal pathways for hydrophilic (group 1) versus hydrophobic (group 2) antivirals.

More than 90% of the total bacteria in activated sludge are heterotrophic [75], primarily using readily available organic carbon such as glucose or organic acids [76]. However, Accinelli et al. isolated two bacterial strains from activated sludge (*Falavobacterium* sp. and *Nocardiodes* sp.) that grew on the SAS oseltamivir as the sole carbon source, achieving higher mineralization after inoculation of the wastewater [77]. This was also confirmed by Lastovčić et al., who isolated three bacterial strains (*Comamonas testosteroni*, *Bacillus amyloliquefaciens*, *Bacillus mycoides*) from activated sludge that grew on DCV, DRV, FAV, LOP, REM, RIT, and umifenovir as sole carbon sources [24]. This aligns with previously mentioned research by Lee et al. [65], which described specific bacterial strains responsible for direct biodegradation and others that further mineralize pharmaceuticals as part of a co-metabolic mechanism. Biotransformation of SASs in activated sludge is also possible, as researched by Prasse et al. on acyclovir and penciclovir. Since these SASs have low log *p* values (acyclovir, log *p* = 0.023 [78]), they have no affinity to adsorb onto sludge flocs, suggesting rapid biotransformation, with the main enzymatic reactions being oxidation of the primary hydroxyl group for both SASs [79]. Another study by the same authors, conducted on SAS oseltamivir (log *p* = 1.1 [80]), found no adsorption onto the flocs due to the low log *p*, but in this case, low removal was detected in WWTP effluents, indicating slow biotransformation [81]. Berthod et al. investigated adsorption coefficients for pharmaceuticals in sewage sludge, highlighting the sorption potential of hydrophobic contaminants. Low sorption potential is defined by log *p* < 2.5, medium sorption potential by 2.5 < log *p* < 4.0, and high sorption potential by log *p* > 4.0 [82]. This correlates well with log *p*, first stage SAS abiotic recoveries (Table 6), and MLVSS observations (Figure 4b). Low sorption potential SASs (DRV, FAV, REM) are further referred to as group 1—hydrophilic, while medium to high sorption potential SASs (DCV, LOP, RIT) are discussed as group 2—hydrophobic (Figure 9).

#### 4.4.1. Group 1—Hydrophilic SASs

DRV, FAV, and REM have low sorption potential (log *p* < 2.5) and were previously presented in group A (biodegradation) in Section 3.3.1. DRV and REM showed a positive increase in MLVSS and abiotic recoveries from the inactivated sludge (24 h, 40–50%), supporting rapid removal by biodegradation, also supported by the research of Prasse et al. [79]. FAV was the only exception to this trend, as MLVSS decreased after 24 h and abiotic controls showed lower recovery than expected, suggesting an unexamined factor influenced a stronger binding of FAV to the sludge. The inhibitory effect on *Aliivibrio fischeri* showed a similar trend as LOP, which will be explained in Section 4.5, Toxicity of SASs and Their Biodegradation Products.

#### 4.4.2. Group 2—Hydrophobic SASs

DCV, LOP, and RIT have a high to medium sorption potential (log *p* > 2.5), supported by our abiotic control recoveries of 0–6% in the first hour of the process (Table 6). Even though LOP exhibited a negative effect on the microbial community, DCV and RIT removal showed no changes in MLVSS, further supporting rapid physical adsorption within the first hour of the process [83]. Additionally, Krasucka et al. studied adsorption and desorption trends for LOP and RIT, confirming relatively low desorption efficiencies (<15%) [84]. This correlates with our findings for LOP; however, RIT followed this trend only at the beginning of the process. After 24 h we determined that RIT abiotic recovery exceeded 90%, which could result from high differentiation in sludge flocs structure. Therefore, RIT was determined to belong to group A (biodegradation), rather than group B (adsorption), as DCV and LOP (Figure 9, Table 8).

In contrast, a study by Betsholtz et al. explored new perspectives on the interactions between adsorption and biodegradation, suggesting that adsorption could extend the insufficient HRT required for pharmaceutical biodegradation to occur [88]. This could be further associated with DCV and LOP removal, with elevated toxicity results of possible byproducts, especially as abiotic recoveries were extremely low at 24 h (<log10%). Furthermore, another possible outcome could be combined removal of pharmaceuticals by rapid adsorption in 2 h, achieving adsorption equilibrium (partial desorption), and potential biodegradation occurring after 12 h [89].

#### 4.4.3. Potential SASs Removal Mechanisms

When these observations are compared to Figure 4a and Figure 5a, which present SAS removal kinetics trends and overall removal from T1 to T27, there is a strong correlation (Table 8). All SASs showed immediate removal after 1 h, except for REM, which was previously defined as group 1A (biodegradation dominant, low sorption potential). At 2 h into the experiment, REM was completely removed, partially by adsorption (Table 6), but primarily through biodegradation or biotransformation as the main process [90,91]. Similar trends were observed for FAV and DRV (group 1A), which showed abiotic recoveries of 10% and 45%, respectively, and potentially active biodegradation. FAV showed lower abiotic recovery than expected from its log *p* value; however, desorption of 20% was observed after 24 h, indicating an adequate amount of available substrate for biodegradation. Xu et al. identified FAV transformation pathways and confirmed that FAV removal occurs through cometabolic biodegradation [86]. Özel Duygan et al. found that primary biodegradation is a successful way to remove DRV from aqueous solution, but with a wide varity of 30–90% at 12 h of experiments. [92]. Nevertheless, group 1A showed lower detected concentrations in the solution than the recovered abiotic controls, therefore, biodegradation was noted for these SASs. Overall, the literature suggests that SAS removal mechanisms follow similar trends at first glance, but it is difficult to discuss common reactions and transformation kinetics for a larger group of SASs without more detailed research on individual trends [93]. Groups 2B and 2A were adequately discussed in previous Section 3.3.1 and Section 3.3.2, and their removal was consistent throughout the experiments, as expected for adsorbed compounds.

### 4.5. Toxicity of SASs and Their Biodegradation Products

Acute toxicity tests of the six selected SASs on the bioluminescence of *Aliivibrio fischeri* did not yield *EC*_20_ or *EC*_50_ values for all samples in the T1–T27 experiments; however, the observed values were higher than the measured toxicity of the initial SAS. Prasse et al. also reported low toxicity for the SAS acyclovir and its main biodegradation product, carboxy-acyclovir, classifying them as non-toxic to *Aliivibrio fischeri* [94]. In contrast, Escher et al. demonstrated an additive toxic effect of oseltamivir ethylester and its metabolite oseltamivir acid toward *Aliivibrio fischeri* [60]. Although studies investigating the ecotoxicity of SASs transformation products remain limited, Xu et al. reported that the main favipiravir metabolite (T-795M1) exhibited approximately 75-fold higher toxicity than the parent compound [86], whereas Bartels et al. found that the principal remdesivir metabolite (GS-441524 [95]) was more persistent but exhibited lower ecotoxicological risk than REM itself [87]. In the present study, the persistence of toxicity despite the high removal efficiencies observed for several investigated SASs may indicate the formation of transformation products during biological treatment. However, this interpretation should be considered a plausible hypothesis rather than direct evidence, since transformation products were not identified or quantified in the present study. Alternative explanations, including the combined effects of intermediate metabolites or residual parent compounds, cannot be excluded. Nevertheless, the observed trends, particularly for Group 1A compounds and potentially for RIT (Group 2A) and LOP (Group 2B), are consistent with previous reports indicating that biological transformation of pharmaceuticals can generate products with ecotoxicological properties different from those of the parent compounds. These findings emphasize that monitoring the removal of parent SASs alone is insufficient for evaluating treatment performance. According to previous studies, operating conditions such as temperature, pH, and MLSS may influence not only the removal of the parent compounds but also the formation and subsequent degradation of transformation products [96]. Therefore, future studies should therefore combine targeted identification of transformation products with ecotoxicological assessment to establish a direct relationship between pharmaceutical biotransformation and the observed toxicity response [97].

### 4.6. Statistical Modeling and Optimization of the Bioremediation Process

The optimization process, conducted using Design Expert software, aimed to maximize SAS removal efficiency through an RSM approach based on a full factorial design (Table 3). Quadratic models provided the best fit for FAV, LOP, and RIT, with *R*^2^ values ranging from 0.8004 to 0.8624, except for DRV, whose model was 2FI (*R*^2^ = 0.5262). This range indicates a highly adequate evaluation of biological experimental data, consistent with the findings of Kandari et al. [98], who demonstrated that comparable *R*^2^ values around 0.64 are statistically sufficient and reliable for modeling complex bacterial biodegradation pathways. Additionally, DCV and REM were evaluated as mean values because of their highly successful removal and the dataset being too narrow to adequately model the responses. RSM optimization achieved a high desirability of 0.772, indicating a reliable and stable optimization output within the acceptable range of 0.6 to 0.8 [99]. The optimal conditions (pH 6.08, 4.41 g/L MLSS, and 44.99 °C) resulted in removal efficiencies exceeding 93% for most tested SASs. Although the RSM model identified 44.99 °C as the optimal temperature for maximizing the removal of the investigated SASs, it should be emphasized that statistically optimal conditions do not necessarily represent the optimal biological operating conditions for activated sludge systems. The predicted optimum reflects the combination of factors that maximized the investigated responses within the experimental design space, whereas long-term process performance also depends on maintaining a stable and metabolically active microbial community. Elevated temperatures generally accelerate biochemical reaction rates, enhance enzyme-mediated biotransformation, and improve mass transfer of substrates to microbial cells. However, prolonged operation at higher temperatures may alter microbial community composition, reduce the abundance of temperature-sensitive microorganisms, and ultimately affect process stability. Therefore, practical implementation of the optimized conditions should balance thermodynamic enhancement of biodegradation with the preservation of microbial viability and functional resilience, particularly in full-scale wastewater treatment plants [37]. The overlay and desirability contour plots (Figure 8) confirm that these operational limits were primarily constrained by the distinct removal characteristics of individual SASs. Specifically, DRV acted as the limiting factor due to its lower maximum removal (66%). Unlike the highly hydrophobic group 2B and 2A compounds, which are predominantly removed via physical adsorption, DRV (group 1A) degradation relies on enzymatic pathways and bacterial co-metabolism, requiring specific thermodynamic and physiological conditions [85]. Consequently, DRV serves as the biological baseline for the entire process, while FAV and RIT further constrain the optimal operating window. Furthermore, the desirability perturbation profile revealed that all three factors influence the optimal point in a specific way as discussed in Section 3.7. This multi-criteria optimization demonstrates that achieving uniform treatment for structurally diverse pharmaceuticals requires navigating strict trade-offs, where a single recalcitrant compound can dictate the operational limits of the entire biological system [92]. Therefore, the optimal conditions obtained in this study provide necessary insight into SASs removal efficiency and general mechanisms for better control of these recalcitrant contaminants and activated sludge disposal in conventional WWTPs. By understanding the nature of their removal, RSM factor interactions, and critical points of operational parameters (pH, MLSS, *T*), adequate compensations can be made if these contaminants are detected in wastewater at higher concentrations. Ultimately, this complex interplay confirms that biological wastewater treatment cannot be evaluated by removal efficiency alone; comprehensive toxicity assessments are essential, as operational conditions directly steer metabolic pathways toward either complete detoxification or the potential generation of more hazardous transformation products [14].

## 5. Conclusions

This study demonstrated that the removal of SASs in activated sludge systems is governed by the combined influence of operational conditions and the physicochemical properties of individual compounds. RSM identified pH, MLSS, and temperature as the key factors controlling removal efficiency, emphasizing the importance of their interactions in optimizing biological treatment. The optimized conditions improved the removal of the investigated SASs; however, the persistence of residual toxicity indicates that high removal efficiency does not necessarily correspond to reduced environmental risk. From a practical perspective, the results suggest that optimization of conventional activated sludge operating conditions may improve wastewater treatment performance without the immediate need for additional advanced treatment technologies. Nevertheless, these findings should be regarded as encouraging laboratory-scale evidence rather than direct guidance for full-scale wastewater treatment plants. A limitation of the present study is that the proposed removal mechanisms and the potential formation of transformation products were inferred from removal efficiency and ecotoxicological responses but were not directly confirmed. Therefore, future research should focus on identifying transformation products, elucidating degradation pathways and validating the proposed mechanisms under continuous and full-scale operating conditions. Overall, this study provides new insight into the removal of structurally diverse SARS-CoV-2 antivirals and highlights the importance of integrating chemical and ecotoxicological assessments when evaluating biological wastewater treatment processes.

## Figures and Tables

**Table 1:** Mass measurements for adjusting phosphate buffer (0.1 M) in mineral medium (1 L).

pH, -	*m* (K_2_HPO_4_), g	*m* (KH_2_PO_4_), g
5.5 *	0.40	13.3
7.0	6.71	8.33
8.5	16.6	0.70

* Theoretical composition (outside the optimal buffering range).

**Table 2:** Target MLSS concentrations and experimental mass determinations.

MLSS, g/L	*m* (Activated Sludge), g
5	11.43
3	6.90
1	2.37

**Table 3:** Coded and actual levels of experimental parameters for the full factorial design.

Experiment	Run	Factor A: pH, -		Factor B: MLSS, g/L		Factor C: *T*, °C	
		Level	Value	Level	Value	Level	Value
T1	2	1	8.5	0	3	−1	10
T2	4	−1	5.5	−1	1	−1	10
T3	6	0	7.0	1	5	−1	10
T4	7	−1	5.5	1	5	−1	10
T5	12	0	7.0	−1	1	−1	10
T6	16	1	8.5	1	5	−1	10
T7	17	1	8.5	−1	1	−1	10
T8	24	−1	5.5	0	3	−1	10
T9	25	0	7.0	0	3	−1	10
T10	5	−1	5.5	0	3	0	30
T11	8	−1	5.5	−1	1	0	30
T12	9	−1	5.5	1	5	0	30
T13	13	1	8.5	0	3	0	30
T14	14	0	7.0	0	3	0	30
T15	20	0	7.0	1	5	0	30
T16	21	1	8.5	1	5	0	30
T17	26	0	7.0	−1	1	0	30
T18	27	1	8.5	−1	1	0	30
T19	1	−1	5.5	0	3	1	45
T20	3	0	7.0	−1	1	1	45
T21	10	1	8.5	−1	1	1	45
T22	11	−1	5.5	−1	1	1	45
T23	15	0	7.0	1	5	1	45
T24	18	1	8.5	1	5	1	45
T25	19	−1	5.5	1	5	1	45
T26	22	1	8.5	0	3	1	45
T27	23	0	7.0	0	3	1	45

**Table 4:** Chromatographic and analytical performance parameters for the determination of SASs.

Parameter	DCV	DRV	FAV	LOP	REM	RIT
Wavelength, nm	304	265	324	215	241	241
Mobile phase B, %	20	55	15	60	40	55
*t*_R_, min	5.86	5.41	4.72	2.84	2.95	8.58
*R* ^2^	0.9958	0.9994	0.9989	0.9962	0.9995	0.9988
LOD, μmol/L	0.246	0.057	0.090	0.007	0.011	0.094
LOQ, μmol/L	0.747	0.173	0.272	0.021	0.034	0.284

Mobile phase B: percentage of organic mobile phase; *t*_R_: retention time; *R*^2^: coefficient of determination; LOD: limit of detection; LOQ: limit of quantification.

**Table 5:** Physicochemical characteristics of activated sludge.

Parameter	Value
pH	7.34 ± 0.51
*T*, °C	22.77 ± 0.60
DO, mg/L	3.30 ± 1.17
EC, mS/cm	0.87 ± 0.16
*w* (H_2_O), %	99.57 ± 0.13
*w* (dry matter), %	0.43 ± 0.13
*w* (volatile matter), %	64.29 ± 6.09
MLSS, g/L	4.34 ± 0.22
MLVSS, %	71.12 ± 3.81

Values represent mean ± SD (*n* = 12).

**Table 6:** Log *p* and abiotic recovery percentages of the studied SASs after 1 and 24 h of incubation.

SAS	Log *p*	Abiotic Recovery at 1 h, %	Abiotic Recovery at 24 h, %
DCV	4.18 ^a^, 4.67 ^b^	0.00 ± 0.00	0.00 ± 0.00
DRV	2.82 ^a^, 1.76 ^b^	40.95 ± 6.08	48.25 ± 0.99
FAV	0.49 ^a^	10.68 ± 3.91	20.48 ± 0.43
LOP	3.91 ^a^	5.97 ± 0.12	7.22 ± 0.13
REM	2.01 ^a^	6.66 ± 3.00	41.82 ± 6.19
RIT	4.24 ^a^, 5.22 ^b^	4.60 ± 0.00	91.13 ± 4.74

^a^ Log *p* data estimated by ChemAxon; ^b^ Log *p* data estimated by ALOGPS. All Log *p* values were sourced from DrugBank (go.drugbank.com (accessed on 31 May 2026)).

**Table 7:** Mathematical models in terms of actual factors and *R*^2^ values for the SAS removal process.

Response	Final Equations in Terms of Actual Factors	R^2^	Adjusted *R*^2^	Predicted *R*^2^	Adeq Precision
DRV removal	= 27.356 + 5.819 ∙ pH + 2.810 ∙ MLSS + 3.162 ∙ *T* − 0.344 ∙ pH ∙ *T* − 0.288 ∙ MLSS ∙ *T*	0.5262	0.4134	0.2250	10.75
FAV removal	= −0.377 + 29.137 ∙ pH + 4.327 ∙ MLSS + 0.854 ∙ *T* − 1.224 ∙ pH ∙ MLSS − 0.147 ∙ pH ∙ *T* + 0.104 ∙ MLSS ∙ *T* − 1.988 pH^2^ − 0.006 *T*^2^	0.8209	0.7413	0.5417	10.69
LOP removal	= 83.834 + 4.185 ∙ pH + 0.289 ∙ MLSS − 0.678 ∙ *T* − 0.165 ∙ pH ∙ MLSS + 0.035 ∙ pH ∙ *T* + 0.015 ∙ MLSS ∙ *T* − 0.298 pH^2^ + 0.102 MLSS^2^ + 0.102 *T*^2^	0.8624	0.7972	0.6314	11.96
RIT removal	= 116.979 − 9.328 ∙ pH + 1.903 ∙ MLSS + 0.999 ∙ *T* + 0.098 ∙ pH ∙ MLSS + 0.039 ∙ pH ∙ *T* + 0.069 ∙ MLSS ∙ *T* + 0.546 pH^2^ − 0.513 MLSS^2^ − 0.028 *T*^2^	0.8004	0.7058	0.5175	10.21

Design-Expert 12 software package was used for data modeling.

**Table 8:** Overview of removal efficiencies and potential degradation mechanisms of the tested SASs.

SAS	Group	Removal, %	Dominant Process	Observations
DRV	1A	4.19–99.21	Biodegradation	Slow biodegradation [85]
FAV	1A	32.22–97.35		Fast biodegradation, toxic products [86]
REM	1A	5.04–95.45		Fast degradation, recalcitrant metabolites [87]
RIT	2A	78.41–100 *		Initial adsorption, biotransformation [84]
LOP	2B	93.55–100 *	Adsorption	Adsorption in two stages, biotransformation [84]
DCV	2B	99–100 *		Adsorption guiding biodegradation [88]

100 *—values below the LOQ.

## Data Availability

The original contributions presented in this study are included in the article. Further inquiries can be directed to the corresponding authors.

## Appendix A

**Table A1:** (**a**) Dynamic variations of measured MLSS at 0 h, 6 h, and 24 h for tested SASs. (**b**) Dynamic variations of MLSS controls at 0 h, 6 h, and 24 h for tested SASs.

(**a**)											
**Measured MLSS, g/L**											
**Exp**			***t*** **, h**			**DCV**	**DRV**	**FAV**	**LOP**	**REM**	**RIT**
T1			0			2.03 ± 0.14	4.14 ± 0.13	2.65 ± 0.16	1.47 ± 0.14	3.54 ± 0.15	2.95 ± 0.18
			6			3.70 ± 0.27	4.56 ± 0.21	2.76 ± 0.22	4.32 ± 0.31	4.90 ± 0.27	3.30 ± 0.25
			24			2.72 ± 0.20	4.00 ± 0.26	3.02 ± 0.22	5.02 ± 0.20	4.92 ± 0.24	1.86 ± 0.13
T2			0			1.09 ± 0.07	1.57 ± 0.22	1.09 ± 0.08	0.90 ± 0.08	1.65 ± 0.19	1.09 ± 0.06
			6			1.34 ± 0.11	2.36 ± 0.10	1.27 ± 0.10	1.64 ± 0.10	1.44 ± 0.11	1.28 ± 0.11
			24			1.26 ± 0.11	2.14 ± 0.19	2.18 ± 0.15	0.86 ± 0.09	1.32 ± 0.11	0.98 ± 0.07
T3			0			4.04 ± 0.29	5.12 ± 0.27	5.13 ± 0.36	3.06 ± 0.22	8.83 ± 0.26	5.72 ± 0.37
			6			4.96 ± 0.38	5.74 ± 0.38	4.18 ± 0.31	5.82 ± 0.40	6.52 ± 0.31	4.20 ± 0.33
			24			4.30 ± 0.26	6.16 ± 0.29	4.30 ± 0.37	6.96 ± 0.38	6.00 ± 0.39	3.96 ± 0.31
T4			0			4.08 ± 0.28	5.11 ± 0.30	5.17 ± 0.35	3.05 ± 0.23	8.80 ± 0.27	5.76 ± 0.35
			6			4.44 ± 0.35	6.98 ± 0.18	2.80 ± 0.24	4.50 ± 0.19	8.08 ± 0.15	3.86 ± 0.26
			24			4.44 ± 0.28	6.52 ± 0.11	4.16 ± 0.30	6.42 ± 0.16	8.26 ± 0.19	3.78 ± 0.29
T5			0			1.05 ± 0.09	1.54 ± 0.22	1.05 ± 0.10	0.86 ± 0.10	1.66 ± 0.18	1.05 ± 0.08
			6			1.08 ± 0.10	2.00 ± 0.15	1.14 ± 0.08	1.24 ± 0.13	2.88 ± 0.22	3.12 ± 0.24
			24			5.40 ± 0.37	2.58 ± 0.21	1.20 ± 0.08	1.76 ± 0.22	2.36 ± 0.14	0.84 ± 0.06
T6			0			4.07 ± 0.30	4.18 ± 0.14	5.18 ± 0.34	3.02 ± 0.24	8.84 ± 0.25	5.75 ± 0.36
			6			5.46 ± 0.33	4.24 ± 0.35	4.50 ± 0.35	4.86 ± 0.30	7.82 ± 0.38	5.28 ± 0.37
			24			5.58 ± 0.32	6.52 ± 0.34	4.48 ± 0.33	6.84 ± 0.33	8.08 ± 0.35	4.22 ± 0.34
T7			0			1.08 ± 0.08	1.58 ± 0.22	1.08 ± 0.09	0.89 ± 0.09	1.62 ± 0.20	1.08 ± 0.07
			6			1.54 ± 0.14	2.36 ± 0.26	1.22 ± 0.07	0.86 ± 0.08	2.64 ± 0.13	1.40 ± 0.09
			24			1.10 ± 0.10	3.10 ± 0.16	1.54 ± 0.10	2.30 ± 0.11	2.48 ± 0.27	0.66 ± 0.05
T8			0			2.02 ± 0.13	5.08 ± 0.29	2.67 ± 0.17	1.46 ± 0.13	3.50 ± 0.13	2.94 ± 0.17
			6			2.10 ± 0.20	6.99 ± 0.29	2.68 ± 0.18	3.60 ± 0.28	5.16 ± 0.29	2.58 ± 0.22
			24			2.10 ± 0.15	3.54 ± 0.24	3.46 ± 0.28	4.22 ± 0.27	4.26 ± 0.28	2.30 ± 0.15
T9			0			2.06 ± 0.15	4.15 ± 0.15	2.62 ± 0.15	1.50 ± 0.15	3.51 ± 0.14	2.98 ± 0.19
			6			3.28 ± 0.22	3.76 ± 0.33	2.70 ± 0.19	4.94 ± 0.26	5.32 ± 0.34	3.12 ± 0.21
			24			2.72 ± 0.21	4.88 ± 0.30	3.74 ± 0.25	5.32 ± 0.29	5.60 ± 0.31	2.82 ± 0.22
T10			0			3.58 ± 0.24	4.14 ± 0.34	2.30 ± 0.15	3.54 ± 0.24	3.50 ± 0.21	2.28 ± 0.15
			6			2.10 ± 0.19	5.46 ± 0.27	2.52 ± 0.18	3.82 ± 0.37	3.00 ± 0.14	3.26 ± 0.25
			24			3.44 ± 0.24	4.76 ± 0.33	2.68 ± 0.18	3.08 ± 0.22	3.64 ± 0.27	2.90 ± 0.21
T11			0			1.08 ± 0.08	1.58 ± 0.10	1.15 ± 0.07	1.40 ± 0.24	1.62 ± 0.12	1.22 ± 0.02
			6			1.34 ± 0.10	2.46 ± 0.14	0.80 ± 0.06	1.98 ± 0.14	1.60 ± 0.09	1.16 ± 0.09
			24			0.10 ± 0.05	3.14 ± 0.24	1.00 ± 0.07	1.76 ± 0.11	2.50 ± 0.13	1.10 ± 0.09
T12			0			4.79 ± 0.35	5.12 ± 0.20	3.38 ± 0.23	5.36 ± 0.30	8.84 ± 0.31	3.78 ± 0.23
			6			4.44 ± 0.38	6.64 ± 0.39	3.78 ± 0.33	4.98 ± 0.29	5.86 ± 0.29	4.50 ± 0.37
			24			4.16 ± 0.35	5.62 ± 0.21	3.86 ± 0.26	6.00 ± 0.38	7.40 ± 0.38	4.42 ± 0.31
T13			0			3.61 ± 0.25	4.18 ± 0.36	2.31 ± 0.16	3.55 ± 0.25	3.51 ± 0.22	2.29 ± 0.16
			6			3.70 ± 0.28	4.68 ± 0.35	3.46 ± 0.26	3.56 ± 0.15	4.74 ± 0.22	2.70 ± 0.19
			24			4.32 ± 0.33	4.88 ± 0.19	3.02 ± 0.24	4.08 ± 0.27	4.78 ± 0.19	2.80 ± 0.19
T14			0			3.62 ± 0.26	4.15 ± 0.35	2.34 ± 0.17	3.58 ± 0.26	3.54 ± 0.23	2.32 ± 0.17
			6			3.28 ± 0.29	4.42 ± 0.39	2.36 ± 0.14	4.36 ± 0.38	5.00 ± 0.27	2.50 ± 0.17
			24			2.82 ± 0.21	4.14 ± 0.22	2.76 ± 0.22	4.08 ± 0.23	4.84 ± 0.44	2.60 ± 0.18
T15			0			4.76 ± 0.36	5.11 ± 0.19	3.34 ± 0.25	5.35 ± 0.31	8.83 ± 0.32	3.74 ± 0.25
			6			4.96 ± 0.31	6.94 ± 0.31	4.62 ± 0.35	5.54 ± 0.19	7.30 ± 0.36	4.50 ± 0.34
			24			2.96 ± 0.27	6.62 ± 0.15	5.06 ± 0.39	6.38 ± 0.20	7.50 ± 0.24	4.08 ± 0.29
T16			0			4.80 ± 0.34	5.08 ± 0.20	3.37 ± 0.24	5.32 ± 0.32	8.80 ± 0.33	3.77 ± 0.24
			6			5.46 ± 0.35	7.02 ± 0.24	4.56 ± 0.33	5.50 ± 0.31	6.30 ± 0.31	3.98 ± 0.28
			24			4.54 ± 0.38	5.92 ± 0.38	5.86 ± 0.35	5.20 ± 0.31	8.24 ± 0.16	3.96 ± 0.27
T17			0			1.05 ± 0.09	1.54 ± 0.12	1.08 ± 0.09	1.43 ± 0.23	1.65 ± 0.11	1.18 ± 0.04
			6			1.08 ± 0.07	1.88 ± 0.29	1.04 ± 0.08	1.32 ± 0.23	1.86 ± 0.13	1.26 ± 0.11
			24			1.80 ± 0.14	2.24 ± 0.31	1.20 ± 0.09	1.62 ± 0.13	1.92 ± 0.29	1.20 ± 0.08
T18			0			1.09 ± 0.07	1.57 ± 0.11	1.03 ± 0.08	1.44 ± 0.22	1.66 ± 0.10	1.21 ± 0.03
			6			1.54 ± 0.15	2.42 ± 0.11	1.50 ± 0.12	1.80 ± 0.22	1.74 ± 0.10	1.28 ± 0.10
			24			1.06 ± 0.09	2.66 ± 0.11	1.48 ± 0.13	1.66 ± 0.14	2.12 ± 0.11	1.26 ± 0.11
T19			0			2.52 ± 0.13	4.18 ± 0.23	3.50 ± 0.22	3.27 ± 0.14	3.06 ± 0.15	1.94 ± 0.13
			6			2.60 ± 0.17	4.92 ± 0.24	3.08 ± 0.25	4.24 ± 0.31	4.78 ± 0.28	2.54 ± 0.19
			24			3.24 ± 0.22	4.32 ± 0.35	1.94 ± 0.15	4.86 ± 0.31	6.36 ± 0.28	2.74 ± 0.19
T20			0			1.00 ± 0.10	1.57 ± 0.14	1.05 ± 0.11	1.28 ± 0.18	0.96 ± 0.07	0.97 ± 0.11
			6			1.44 ± 0.12	2.22 ± 0.12	1.26 ± 0.10	2.06 ± 0.11	1.62 ± 0.14	1.14 ± 0.09
			24			1.54 ± 0.14	2.44 ± 0.30	0.96 ± 0.09	2.44 ± 0.28	3.10 ± 0.19	0.96 ± 0.08
T21			0			1.08 ± 0.10	1.58 ± 0.13	1.08 ± 0.03	1.27 ± 0.19	0.95 ± 0.08	1.00 ± 0.10
			6			1.54 ± 0.14	2.50 ± 0.15	1.34 ± 0.13	2.20 ± 0.24	2.48 ± 0.15	1.16 ± 0.11
			24			1.02 ± 0.07	2.48 ± 0.26	0.84 ± 0.06	1.22 ± 0.09	3.50 ± 0.15	1.02 ± 0.10
T22			0			1.03 ± 0.08	1.54 ± 0.15	1.09 ± 0.04	1.24 ± 0.20	0.92 ± 0.09	1.01 ± 0.09
			6			1.42 ± 0.09	2.64 ± 0.31	1.12 ± 0.08	2.02 ± 0.27	1.60 ± 0.19	1.06 ± 0.07
			24			1.58 ± 0.09	2.22 ± 0.11	1.04 ± 0.07	1.97 ± 0.11	2.72 ± 0.11	0.94 ± 0.07
T23			0			4.18 ± 0.32	5.11 ± 0.36	3.98 ± 0.30	4.36 ± 0.27	4.98 ± 0.31	4.22 ± 0.33
			6			4.40 ± 0.34	7.38 ± 0.39	4.08 ± 0.34	6.98 ± 0.19	7.10 ± 0.33	3.86 ± 0.26
			24			4.64 ± 0.31	7.02 ± 0.19	4.08 ± 0.31	11.00 ± 0.39	9.68 ± 0.38	3.92 ± 0.31
T24			0			4.21 ± 0.31	5.08 ± 0.40	4.01 ± 0.29	4.32 ± 0.29	4.97 ± 0.32	4.25 ± 0.32
			6			5.16 ± 0.31	7.04 ± 0.22	4.00 ± 0.32	7.04 ± 0.35	5.80 ± 0.26	4.24 ± 0.34
			24			5.42 ± 0.36	7.94 ± 0.31	3.94 ± 0.35	7.02 ± 0.26	7.42 ± 0.31	4.08 ± 0.34
T25			0			4.22 ± 0.30	5.12 ± 0.35	4.02 ± 0.28	4.35 ± 0.28	4.94 ± 0.33	4.26 ± 0.31
			6			3.78 ± 0.29	7.66 ± 0.34	4.06 ± 0.29	6.60 ± 0.16	6.38 ± 0.31	4.08 ± 0.28
			24			4.36 ± 0.29	7.32 ± 0.16	4.06 ± 0.29	8.24 ± 0.18	6.92 ± 0.24	3.98 ± 0.30
T26			0			2.53 ± 0.14	4.15 ± 0.22	3.51 ± 0.23	3.26 ± 0.13	3.09 ± 0.16	1.95 ± 0.14
			6			3.18 ± 0.25	5.82 ± 0.28	2.88 ± 0.23	5.82 ± 0.33	5.08 ± 0.24	2.76 ± 0.23
			24			4.06 ± 0.27	5.70 ± 0.33	2.30 ± 0.18	7.90 ± 0.20	6.32 ± 0.29	2.90 ± 0.20
T27			0			2.56 ± 0.15	4.14 ± 0.21	3.54 ± 0.30	3.30 ± 0.15	3.10 ± 0.17	1.98 ± 0.15
			6			2.84 ± 0.22	4.44 ± 0.19	2.90 ± 0.21	4.78 ± 0.29	4.38 ± 0.18	2.58 ± 0.18
			24			3.76 ± 0.25	4.50 ± 0.24	2.72 ± 0.23	7.02 ± 0.33	6.12 ± 0.17	2.80 ± 0.22
(**b**)											
**MLSS Control, g/L**											
***T***		**Exp**		***t, h***		**DCV**	**DRV**	**FAV**	**LOP**	**REM**	**RIT**
10 °C		C(−1)		0		1.07 ± 0.08	1.56 ± 0.22	1.09 ± 0.09	0.88 ± 0.09	1.64 ± 0.19	1.01 ± 0.05
				6		1.54 ± 0.12	3.06 ± 0.22	1.24 ± 0.09	1.82 ± 0.30	2.80 ± 0.24	0.58 ± 0.05
				24		0.16 ± 0.05	1.82 ± 0.12	1.46 ± 0.12	2.16 ± 0.14	2.40 ± 0.22	1.00 ± 0.08
		C0		0		1.10 ± 0.08	1.56 ± 0.11	1.02 ± 0.08	1.42 ± 0.23	1.64 ± 0.11	1.20 ± 0.03
				6		1.54 ± 0.11	2.18 ± 0.22	1.22 ± 0.11	1.66 ± 0.12	1.66 ± 0.12	1.06 ± 0.08
				24		0.96 ± 0.08	2.08 ± 0.36	1.28 ± 0.10	1.08 ± 0.09	1.96 ± 0.14	0.82 ± 0.06
		C1		0		1.05 ± 0.09	1.56 ± 0.14	1.07 ± 0.11	1.26 ± 0.19	0.94 ± 0.08	0.99 ± 0.10
				6		1.40 ± 0.11	3.26 ± 0.27	1.32 ± 0.09	2.90 ± 0.14	1.28 ± 0.11	1.04 ± 0.08
				24		1.46 ± 0.12	2.36 ± 0.11	1.10 ± 0.08	2.48 ± 0.14	2.52 ± 0.14	1.10 ± 0.10
30 °C		C(−1)		0		2.04 ± 0.14	4.16 ± 0.14	2.64 ± 0.16	1.48 ± 0.14	3.52 ± 0.14	2.96 ± 0.18
				6		3.10 ± 0.24	3.78 ± 0.14	2.92 ± 0.21	4.10 ± 0.15	5.10 ± 0.18	2.86 ± 0.19
				24		2.50 ± 0.20	3.76 ± 0.15	3.24 ± 0.26	4.08 ± 0.20	5.06 ± 0.16	2.66 ± 0.19
		C0		0		3.60 ± 0.25	4.16 ± 0.35	2.32 ± 0.16	3.56 ± 0.25	3.52 ± 0.22	2.30 ± 0.16
				6		3.10 ± 0.30	5.10 ± 0.30	2.64 ± 0.21	3.60 ± 0.22	3.10 ± 0.29	3.00 ± 0.22
				24		2.76 ± 0.17	4.82 ± 0.43	4.18 ± 0.12	3.38 ± 0.29	4.54 ± 0.34	3.38 ± 0.24
		C1		0		2.54 ± 0.14	4.16 ± 0.22	3.52 ± 0.23	3.28 ± 0.14	3.08 ± 0.16	1.96 ± 0.14
				6		3.50 ± 0.26	6.04 ± 0.18	3.00 ± 0.22	5.04 ± 0.22	4.20 ± 0.22	3.14 ± 0.22
				24		6.08 ± 0.38	5.78 ± 0.22	2.78 ± 0.20	4.90 ± 0.22	4.84 ± 0.22	2.82 ± 0.21
45 °C		C(−1)		0		4.06 ± 0.30	5.10 ± 0.28	5.16 ± 0.35	3.04 ± 0.23	8.82 ± 0.26	5.74 ± 0.36
				6		4.34 ± 0.31	5.82 ± 0.31	4.58 ± 0.33	7.00 ± 0.32	7.74 ± 0.36	3.74 ± 0.28
				24		5.78 ± 0.34	8.64 ± 0.38	4.82 ± 0.34	5.60 ± 0.35	6.86 ± 0.34	3.86 ± 0.27
		C0		0		4.78 ± 0.35	5.10 ± 0.19	3.36 ± 0.24	5.34 ± 0.31	8.82 ± 0.32	3.76 ± 0.24
				6		4.34 ± 0.34	7.46 ± 0.32	3.90 ± 0.28	5.72 ± 0.34	6.76 ± 0.33	4.38 ± 0.33
				24		4.16 ± 0.31	7.16 ± 0.28	4.18 ± 0.32	5.14 ± 0.35	7.00 ± 0.35	4.52 ± 0.33
		C1		0		4.20 ± 0.31	5.10 ± 0.36	4.00 ± 0.29	4.34 ± 0.28	4.96 ± 0.32	4.24 ± 0.32
				6		4.90 ± 0.38	7.24 ± 0.35	4.06 ± 0.31	8.44 ± 0.38	7.10 ± 0.35	4.26 ± 0.35
				24		5.02 ± 0.34	7.62 ± 0.40	4.40 ± 0.34	8.30 ± 0.35	7.20 ± 0.35	3.94 ± 0.33

Values are presented as mean ± SD (*n* = 3). DCV: daclatasvir; DRV: darunavir; FAV: favipiravir; LOP: lopinavir; REM: remdesivir; RIT: ritonavir; Exp: experimental run; TX: test X = number; CX: control X = MLSS level.

**Table A2:** (**a**) Dynamic variations of measured MLVSS at 0 h, 6 h, and 24 h for tested SASs. (**b**) Dynamic variations of MLVSS control at 0 h, 6 h, and 24 h for tested SASs.

(**a**)											
**Measured MLVSS, %**											
**Exp**			***t*** **, h**			**DCV**	**DRV**	**FAV**	**LOP**	**REM**	**RIT**
T1			0			61.35 ± 4.15	83.95 ± 2.30	84.15 ± 2.64	86.50 ± 2.45	73.35 ± 3.35	83.50 ± 2.65
			6			60.78 ± 4.21	60.07 ± 4.32	68.84 ± 3.72	46.30 ± 4.72	92.64 ± 1.14	83.27 ± 2.61
			24			74.84 ± 3.23	77.00 ± 3.18	72.19 ± 3.51	63.35 ± 4.17	56.33 ± 4.55	73.98 ± 3.35
T2			0			78.42 ± 2.96	73.60 ± 3.32	75.20 ± 3.26	94.50 ± 0.84	61.65 ± 4.22	61.65 ± 4.24
			6			76.17 ± 3.15	63.56 ± 4.13	74.60 ± 3.24	39.02 ± 4.90	55.56 ± 4.53	55.56 ± 4.51
			24			79.22 ± 2.92	60.75 ± 4.29	67.89 ± 3.82	41.86 ± 4.83	57.58 ± 4.44	57.58 ± 4.43
T3			0			64.55 ± 4.05	83.25 ± 2.47	90.24 ± 2.15	93.80 ± 0.92	81.20 ± 2.84	73.35 ± 3.34
			6			67.00 ± 3.88	76.31 ± 3.14	67.94 ± 3.83	48.11 ± 4.77	93.36 ± 1.08	92.64 ± 2.10
			24			79.26 ± 2.90	50.32 ± 4.81	66.51 ± 3.98	27.87 ± 5.02	96.71 ± 0.87	56.33 ± 4.52
T4			0			69.45 ± 3.65	86.95 ± 2.09	86.50 ± 2.45	92.55 ± 2.03	69.85 ± 3.67	72.10 ± 3.47
			6			67.00 ± 3.87	63.90 ± 4.18	65.71 ± 4.00	41.33 ± 2.89	83.51 ± 2.69	90.10 ± 2.22
			24			79.26 ± 2.94	70.55 ± 3.62	78.37 ± 3.05	28.66 ± 5.01	96.53 ± 0.88	98.55 ± 1.67
T5			0			73.92 ± 3.31	69.98 ± 3.67	71.96 ± 3.50	98.15 ± 0.56	72.10 ± 3.48	65.15 ± 3.96
			6			76.17 ± 3.11	70.00 ± 3.68	68.42 ± 3.71	48.39 ± 4.72	90.10 ± 2.29	81.25 ± 2.85
			24			79.22 ± 2.98	56.59 ± 4.54	18.33 ± 5.06	42.05 ± 4.88	98.55 ± 0.34	86.44 ± 2.49
T6			0			68.20 ± 3.77	84.80 ± 2.21	88.95 ± 2.29	90.30 ± 2.27	65.15 ± 3.98	69.85 ± 1.62
			6			67.00 ± 3.85	66.98 ± 3.80	64.00 ± 4.17	36.21 ± 5.03	81.25 ± 2.88	83.51 ± 2.68
			24			79.26 ± 2.97	63.19 ± 4.14	73.66 ± 3.46	30.99 ± 5.05	86.44 ± 2.43	96.53 ± 0.81
T7			0			75.05 ± 3.20	70.85 ± 3.63	74.45 ± 3.38	96.85 ± 0.66	63.95 ± 4.03	63.95 ± 4.04
			6			76.17 ± 3.12	64.41 ± 4.04	95.08 ± 1.88	46.51 ± 4.74	79.55 ± 2.92	79.55 ± 2.99
			24			79.22 ± 2.99	78.06 ± 3.00	92.21 ± 2.15	71.30 ± 3.59	71.77 ± 1.55	71.77 ± 3.52
T8			0			59.01 ± 4.35	82.50 ± 2.48	83.10 ± 2.72	84.20 ± 2.69	84.70 ± 2.55	81.20 ± 2.83
			6			60.78 ± 4.26	73.69 ± 3.38	75.37 ± 3.21	41.67 ± 4.84	93.98 ± 1.03	93.36 ± 2.03
			24			74.84 ± 3.27	71.19 ± 3.50	78.61 ± 3.07	44.08 ± 4.86	90.71 ± 2.24	96.71 ± 0.86
T9			0			62.55 ± 4.11	85.76 ± 2.14	86.60 ± 2.47	87.75 ± 2.31	83.50 ± 2.64	84.70 ± 2.55
			6			60.78 ± 4.20	60.11 ± 4.36	69.63 ± 3.69	58.70 ± 4.35	83.27 ± 2.65	93.98 ± 2.01
			24			74.84 ± 3.22	61.48 ± 4.26	79.68 ± 2.98	75.94 ± 3.27	73.98 ± 3.31	90.71 ± 2.21
T10			0			73.25 ± 3.34	82.40 ± 2.42	54.20 ± 4.71	86.45 ± 2.58	73.40 ± 3.33	61.15 ± 2.88
			6			79.05 ± 2.92	65.20 ± 4.00	70.63 ± 3.62	52.36 ± 4.78	97.95 ± 0.60	48.67 ± 4.79
			24			76.16 ± 3.15	60.92 ± 4.21	64.93 ± 4.07	99.35 ± 0.21	48.38 ± 4.77	43.41 ± 4.85
T11			0			76.45 ± 3.10	73.55 ± 3.35	79.50 ± 2.81	25.70 ± 4.82	61.60 ± 4.29	65.20 ± 3.97
			6			77.61 ± 3.05	89.43 ± 2.32	42.50 ± 5.03	28.28 ± 5.06	79.31 ± 2.91	50.00 ± 4.82
			24			80.00 ± 2.81	53.50 ± 4.75	72.00 ± 3.55	62.50 ± 4.18	41.51 ± 4.82	47.20 ± 4.80
T12			0			70.20 ± 3.66	83.35 ± 2.36	51.80 ± 4.95	91.85 ± 0.15	81.15 ± 2.84	63.40 ± 3.34
			6			80.18 ± 2.84	71.08 ± 3.52	69.31 ± 3.72	33.33 ± 5.07	48.67 ± 4.78	97.95 ± 1.64
			24			72.12 ± 3.57	70.46 ± 3.61	59.07 ± 4.30	67.67 ± 3.95	43.41 ± 4.88	48.38 ± 4.78
T13			0			74.50 ± 3.22	85.85 ± 2.17	55.45 ± 4.67	89.95 ± 2.25	84.75 ± 2.51	64.75 ± 2.51
			6			80.00 ± 2.85	54.27 ± 4.61	62.43 ± 4.15	19.66 ± 5.09	76.37 ± 3.19	76.37 ± 1.17
			24			75.93 ± 3.15	81.15 ± 2.82	21.19 ± 5.01	61.27 ± 4.24	36.40 ± 5.04	36.40 ± 5.09
T14			0			76.75 ± 3.07	84.70 ± 2.29	57.86 ± 4.33	88.75 ± 2.36	83.50 ± 2.62	63.50 ± 2.62
			6			82.32 ± 2.74	70.59 ± 3.68	63.56 ± 4.10	46.33 ± 4.81	87.60 ± 2.44	87.60 ± 2.40
			24			75.89 ± 3.16	74.40 ± 3.33	45.65 ± 4.94	51.47 ± 4.86	58.26 ± 4.40	58.26 ± 4.41
T15			0			73.74 ± 3.35	85.65 ± 2.19	55.34 ± 4.53	94.20 ± 0.94	69.80 ± 3.63	62.15 ± 3.45
			6			79.84 ± 2.82	73.20 ± 3.37	67.53 ± 3.87	92.78 ± 2.04	79.37 ± 2.94	85.21 ± 2.34
			24			72.30 ± 3.41	20.54 ± 5.05	73.96 ± 3.33	58.93 ± 4.39	52.67 ± 4.71	53.87 ± 4.71
T16			0			71.15 ± 3.63	86.85 ± 2.08	52.95 ± 4.85	95.40 ± 0.83	72.15 ± 3.44	69.80 ± 3.66
			6			79.85 ± 2.83	59.83 ± 4.37	62.28 ± 4.18	91.27 ± 2.14	85.21 ± 2.33	79.37 ± 2.98
			24			71.81 ± 3.53	68.58 ± 3.70	55.29 ± 4.66	48.46 ± 4.80	53.87 ± 4.72	52.67 ± 4.72
T17			0			79.81 ± 2.82	72.35 ± 3.45	76.10 ± 3.15	24.50 ± 4.98	65.20 ± 3.96	64.00 ± 4.05
			6			79.63 ± 2.90	65.96 ± 3.91	59.62 ± 4.39	69.70 ± 1.73	50.00 ± 4.85	54.84 ± 4.69
			24			80.00 ± 2.84	91.96 ± 2.18	61.67 ± 4.26	50.62 ± 4.85	47.20 ± 4.88	65.63 ± 4.07
T18			0			77.50 ± 3.00	70.05 ± 3.60	78.25 ± 2.90	22.20 ± 5.17	64.00 ± 4.07	61.60 ± 4.24
			6			79.22 ± 2.99	96.69 ± 0.89	70.67 ± 3.64	17.78 ± 5.08	54.84 ± 4.68	79.31 ± 2.99
			24			79.25 ± 2.90	53.38 ± 4.74	83.78 ± 2.68	69.88 ± 3.79	65.63 ± 4.07	41.51 ± 4.87
T19			0			73.10 ± 3.41	82.40 ± 2.46	68.70 ± 3.78	44.55 ± 4.89	74.85 ± 3.03	78.75 ± 3.18
			6			75.38 ± 3.21	69.92 ± 3.72	75.32 ± 3.28	65.09 ± 4.02	62.96 ± 4.11	80.75 ± 2.88
			24			74.07 ± 3.39	72.69 ± 3.47	55.67 ± 4.63	60.08 ± 4.22	71.61 ± 3.54	78.30 ± 1.00
T20			0			79.70 ± 2.88	70.05 ± 3.65	72.35 ± 3.49	45.85 ± 4.84	78.35 ± 2.74	74.85 ± 3.05
			6			79.17 ± 2.94	75.68 ± 3.26	74.60 ± 3.27	74.76 ± 3.35	65.00 ± 4.01	62.96 ± 4.10
			24			77.92 ± 3.04	68.03 ± 3.72	66.67 ± 3.90	73.77 ± 3.41	83.82 ± 2.75	71.61 ± 3.50
T21			0			78.50 ± 2.95	72.35 ± 3.44	74.65 ± 3.28	48.20 ± 4.68	78.75 ± 1.12	77.15 ± 2.82
			6			80.52 ± 2.80	91.20 ± 1.90	64.18 ± 4.03	82.73 ± 2.74	80.75 ± 2.82	67.74 ± 3.88
			24			80.39 ± 2.89	58.87 ± 4.33	76.19 ± 3.19	59.02 ± 4.36	78.30 ± 3.06	56.57 ± 4.62
T22			0			76.25 ± 3.15	73.55 ± 3.34	75.85 ± 3.17	49.40 ± 4.57	72.85 ± 3.12	78.35 ± 2.79
			6			80.28 ± 2.83	84.85 ± 2.33	73.21 ± 3.38	61.39 ± 4.29	64.51 ± 4.03	65.00 ± 4.09
			24			78.48 ± 3.00	54.05 ± 4.74	78.85 ± 2.99	68.50 ± 3.87	58.47 ± 4.46	83.82 ± 2.74
T23			0			71.75 ± 3.57	83.35 ± 2.34	60.25 ± 4.24	38.30 ± 5.04	77.15 ± 2.80	72.85 ± 3.17
			6			69.09 ± 3.70	66.12 ± 3.90	63.24 ± 4.16	59.31 ± 4.33	67.74 ± 3.85	64.51 ± 4.04
			24			66.81 ± 3.90	77.49 ± 3.00	53.92 ± 4.72	79.64 ± 3.08	56.57 ± 4.60	58.47 ± 4.40
T24			0			70.60 ± 3.60	86.85 ± 2.05	59.10 ± 4.32	41.85 ± 4.73	76.35 ± 2.87	76.35 ± 2.86
			6			67.83 ± 3.88	64.49 ± 4.03	60.50 ± 4.31	56.25 ± 4.58	77.59 ± 3.04	77.59 ± 3.01
			24			66.05 ± 4.06	38.79 ± 4.93	53.30 ± 4.73	89.74 ± 2.39	64.96 ± 4.04	64.96 ± 4.03
T25			0			68.25 ± 3.83	85.65 ± 2.17	56.75 ± 4.62	40.65 ± 4.83	75.15 ± 2.95	75.15 ± 2.93
			6			69.84 ± 3.65	66.84 ± 3.97	66.50 ± 3.97	63.33 ± 4.19	73.98 ± 3.30	73.98 ± 3.37
			24			68.81 ± 3.76	64.75 ± 4.04	54.19 ± 4.79	86.89 ± 2.54	57.80 ± 4.53	57.80 ± 4.54
T26			0			75.35 ± 3.22	84.70 ± 2.21	71.05 ± 3.57	46.90 ± 4.62	82.25 ± 2.81	81.10 ± 2.98
			6			76.10 ± 3.19	65.98 ± 3.95	60.42 ± 4.33	89.35 ± 2.30	92.69 ± 0.23	83.86 ± 2.76
			24			74.88 ± 3.25	57.19 ± 4.58	52.17 ± 4.87	64.30 ± 4.03	60.13 ± 4.38	56.96 ± 4.66
T27			0			76.50 ± 3.10	85.85 ± 2.15	72.20 ± 3.49	48.10 ± 4.58	81.10 ± 2.93	82.25 ± 2.84
			6			73.94 ± 3.30	62.61 ± 4.19	62.07 ± 4.12	74.48 ± 3.31	83.86 ± 2.78	92.69 ± 0.29
			24			72.87 ± 3.44	68.00 ± 3.79	71.32 ± 3.58	67.52 ± 3.83	56.96 ± 4.67	60.13 ± 4.39
(**b**)											
**MLVSS Control, %**											
***T***		**Exp**		***t*** **, h**		**DCV**	**DRV**	**FAV**	**LOP**	**REM**	**RIT**
10 °C		C(−1)		0		76.17 ± 3.12	71.79 ± 3.51	73.58 ± 3.46	96.32 ± 0.70	63.41 ± 4.18	93.18 ± 2.07
				6		79.22 ± 2.91	85.62 ± 2.27	75.81 ± 3.21	45.05 ± 4.80	57.14 ± 4.46	27.59 ± 5.02
				24		62.50 ± 4.06	72.53 ± 3.48	94.52 ± 1.98	42.59 ± 4.84	90.83 ± 2.20	74.00 ± 3.30
		C0		0		78.13 ± 2.95	71.79 ± 3.51	77.78 ± 3.03	23.94 ± 5.01	63.40 ± 4.18	60.78 ± 4.21
				6		80.52 ± 2.88	31.19 ± 5.05	70.49 ± 3.60	34.94 ± 5.07	98.34 ± 0.56	67.92 ± 3.81
				24		79.17 ± 2.91	75.00 ± 3.26	70.31 ± 3.64	50.00 ± 4.84	98.13 ± 0.54	51.22 ± 4.88
		C1		0		77.97 ± 3.00	71.79 ± 3.50	74.11 ± 3.25	47.62 ± 4.79	76.60 ± 2.89	67.39 ± 3.87
				6		80.00 ± 2.85	66.26 ± 3.90	77.27 ± 3.08	65.52 ± 3.98	82.81 ± 2.77	63.46 ± 4.06
				24		79.45 ± 2.90	57.63 ± 4.43	74.55 ± 3.29	69.35 ± 3.70	58.73 ± 4.49	63.64 ± 4.02
30 °C		C(−1)		0		60.78 ± 4.28	84.13 ± 2.30	84.85 ± 2.60	85.98 ± 2.53	82.95 ± 2.78	57.43 ± 4.51
				6		74.84 ± 3.28	68.78 ± 3.72	73.97 ± 3.37	46.83 ± 4.73	79.22 ± 2.98	64.34 ± 4.05
				24		75.20 ± 3.21	63.30 ± 4.11	85.80 ± 2.52	65.69 ± 1.93	81.42 ± 2.82	63.91 ± 4.01
		C0		0		75.00 ± 3.27	84.13 ± 2.35	56.03 ± 4.57	88.20 ± 2.41	82.95 ± 2.77	59.13 ± 4.30
				6		80.00 ± 2.89	61.96 ± 4.14	66.67 ± 3.91	38.89 ± 4.98	66.45 ± 3.97	64.67 ± 4.02
				24		76.09 ± 3.11	54.36 ± 4.67	60.77 ± 4.22	98.41 ± 0.27	34.80 ± 5.03	54.44 ± 4.71
		C1		0		74.80 ± 3.29	84.13 ± 2.32	70.45 ± 3.60	46.34 ± 4.70	80.52 ± 2.97	57.14 ± 4.51
				6		74.86 ± 3.25	65.23 ± 4.05	66.67 ± 3.91	61.51 ± 4.25	69.05 ± 3.76	54.78 ± 4.63
				24		73.03 ± 3.41	70.93 ± 3.67	53.96 ± 4.79	48.16 ± 4.81	70.25 ± 3.69	53.19 ± 4.71
45 °C		C(−1)		0		67.00 ± 3.89	85.10 ± 2.22	88.37 ± 2.37	92.04 ± 0.19	71.61 ± 3.55	54.70 ± 4.77
				6		79.26 ± 2.99	98.07 ± 0.50	87.34 ± 2.48	39.71 ± 4.96	89.15 ± 2.31	67.91 ± 3.87
				24		69.90 ± 3.65	72.45 ± 3.49	49.79 ± 4.81	30.36 ± 5.02	94.17 ± 0.99	67.36 ± 3.82
		C0		0		71.97 ± 3.50	85.10 ± 2.27	53.57 ± 4.76	93.63 ± 2.00	71.61 ± 3.58	60.64 ± 4.24
				6		80.18 ± 2.87	63.27 ± 4.14	63.08 ± 4.11	38.42 ± 4.91	90.83 ± 0.27	54.34 ± 4.77
				24		72.12 ± 3.58	82.12 ± 2.73	57.42 ± 4.46	84.44 ± 2.53	39.14 ± 4.98	58.85 ± 4.38
		C1		0		70.00 ± 3.65	85.10 ± 2.20	58.50 ± 4.49	40.09 ± 4.92	74.60 ± 3.01	61.79 ± 4.17
				6		69.80 ± 3.65	77.35 ± 3.09	63.05 ± 4.18	75.36 ± 3.26	77.46 ± 3.00	60.09 ± 4.23
				24		66.93 ± 3.98	76.38 ± 3.10	54.55 ± 4.61	49.64 ± 4.82	76.94 ± 3.05	53.81 ± 4.73

Values are presented as mean ± SD (*n* = 3). DCV: daclatasvir; DRV: darunavir; FAV: favipiravir; LOP: lopinavir; REM: remdesivir; RIT: ritonavir; Exp: experimental run; TX: test X = number; CX: control X = MLSS level.

**Table A3:** Removal efficiency of tested SASs after 24 h of the removal process.

Removal, %						
Exp	DCV	DRV	FAV	LOP	REM	RIT
T1	100 *	78.89 ± 1.24	80.91 ± 1.12	93.81 ± 0.15	99.37 ± 0.15	91.00 ± 0.63
T2	100 *	72.66 ± 1.54	97.35 ± 0.34	93.69 ± 0.12	94.28 ± 0.65	95.40 ± 0.31
T3	100 *	74.21 ± 0.93	89.88 ± 0.88	93.55 ± 0.18	99.37 ± 0.12	94.83 ± 0.45
T4	100 *	64.52 ± 2.19	97.35 ± 0.39	93.60 ± 0.14	99.37 ± 0.18	94.85 ± 0.42
T5	100 *	65.46 ± 1.87	97.35 ± 0.24	93.85 ± 0.16	99.37 ± 0.17	89.63 ± 0.87
T6	100 *	72.46 ± 1.32	71.96 ± 1.41	94.01 ± 0.11	99.54 ± 0.15	95.40 ± 0.38
T7	100 *	77.88 ± 0.84	97.35 ± 0.41	93.87 ± 0.13	82.19 ± 1.13	89.52 ± 0.90
T8	100 *	70.69 ± 1.48	51.05 ± 1.95	93.61 ± 0.17	99.37 ± 0.14	95.40 ± 0.32
T9	100 *	77.74 ± 1.16	94.20 ± 0.68	93.77 ± 0.12	99.37 ± 0.10	91.03 ± 0.70
T10	100 *	85.73 ± 0.77	97.35 ± 0.33	93.62 ± 0.15	99.37 ± 0.16	100 *
T11	100 *	75.71 ± 1.24	97.35 ± 0.22	93.56 ± 0.19	99.37 ± 0.19	100 *
T12	100 *	98.26 ± 0.21	97.35 ± 0.33	93.56 ± 0.14	99.37 ± 0.13	100 *
T13	100 *	4.19 ± 0.38	78.09 ± 1.25	93.71 ± 0.13	34.55 ± 1.85	100 *
T14	100 *	85.38 ± 0.69	91.36 ± 0.74	93.68 ± 0.16	95.63 ± 0.55	100 *
T15	100 *	44.29 ± 1.94	84.88 ± 1.10	93.67 ± 0.12	99.37 ± 0.11	100 *
T16	100 *	53.64 ± 1.72	76.25 ± 1.44	93.84 ± 0.14	5.04 ± 0.45	100 *
T17	100 *	81.98 ± 0.91	97.35 ± 0.24	93.84 ± 0.11	99.37 ± 0.17	100 *
T18	100 *	99.21 ± 0.13	89.83 ± 0.98	93.74 ± 0.15	35.81 ± 1.95	100 *
T19	100 *	92.75 ± 0.55	97.35 ± 0.36	93.68 ± 0.13	99.54 ± 0.12	95.40 ± 0.43
T20	100 *	96.92 ± 0.40	79.48 ± 1.29	100 *	99.54 ± 0.14	78.41 ± 1.21
T21	100 *	73.38 ± 1.37	72.94 ± 1.33	100 *	99.54 ± 0.12	83.45 ± 0.95
T22	100 *	84.70 ± 0.85	97.35 ± 0.28	93.73 ± 0.14	99.54 ± 0.13	80.44 ± 1.10
T23	100 *	99.01 ± 0.18	97.35 ± 0.31	100 *	94.20 ± 0.70	93.67 ± 0.57
T24	100 *	24.09 ± 2.29	32.22 ± 2.15	100 *	99.54 ± 0.11	95.40 ± 0.36
T25	100 *	52.58 ± 1.63	97.35 ± 0.34	100 *	99.54 ± 0.12	95.40 ± 0.34
T26	100 *	45.51 ± 1.82	63.06 ± 1.74	100 *	99.54 ± 0.15	94.41 ± 0.48
T27	100 *	90.36 ± 0.60	97.35 ± 0.27	100 *	98.50 ± 0.40	95.40 ± 0.39

Values are presented as mean ± SD (*n* = 3). DCV: daclatasvir; DRV: darunavir; FAV: favipiravir; LOP: lopinavir; REM: remdesivir; RIT: ritonavir; Exp: experimental run; TX: test X = number. n.d.—not detected; 100 *—values below the LOQ.

**Table A4:** Acute toxicity assessment of tested SASs at 0 and 24 h of the removal process.

*INH*, %						
Exp	DCV	DRV	FAV	LOP	REM	RIT
T1	44.70 ± 1.83	19.84 ± 2.45	8.59 ± 2.87	93.81 ± 0.15	99.37 ± 0.15	91.00 ± 0.63
T2	64.56 ± 1.42	44.01 ± 1.86	29.47 ± 2.24	93.69 ± 0.12	94.28 ± 0.65	95.40 ± 0.31
T3	46.38 ± 1.70	40.79 ± 1.92	13.18 ± 2.65	93.55 ± 0.18	99.37 ± 0.12	94.83 ± 0.45
T4	42.88 ± 1.94	50.42 ± 1.68	25.67 ± 2.35	93.60 ± 0.14	99.37 ± 0.18	94.85 ± 0.42
T5	32.31 ± 2.10	55.67 ± 1.45	23.76 ± 2.40	93.85 ± 0.16	99.37 ± 0.17	89.63 ± 0.87
T6	49.80 ± 1.61	27.78 ± 2.28	3.18 ± 2.98	94.01 ± 0.11	99.54 ± 0.15	95.40 ± 0.38
T7	45.06 ± 1.78	22.62 ± 2.35	34.37 ± 2.07	93.87 ± 0.13	82.19 ± 1.13	89.52 ± 0.90
T8	44.89 ± 1.89	42.72 ± 1.99	28.22 ± 2.32	93.61 ± 0.17	99.37 ± 0.14	95.40 ± 0.32
T9	45.95 ± 1.61	38.17 ± 2.04	21.00 ± 2.51	93.77 ± 0.12	99.37 ± 0.10	91.03 ± 0.70
T10	34.73 ± 2.05	55.41 ± 1.52	n.d.	93.62 ± 0.15	99.37 ± 0.16	100 *
T11	56.02 ± 1.38	31.06 ± 2.13	2.82 ± 2.99	93.56 ± 0.19	99.37 ± 0.19	100 *
T12	n.d.	47.02 ± 1.71	n.d.	93.56 ± 0.14	99.37 ± 0.13	100 *
T13	n.d.	n.d.	29.40 ± 2.27	93.71 ± 0.13	34.55 ± 1.85	100 *
T14	n.d.	16.65 ± 2.68	34.67 ± 2.02	93.68 ± 0.16	95.63 ± 0.55	100 *
T15	n.d.	36.39 ± 2.10	35.63 ± 1.98	93.67 ± 0.12	99.37 ± 0.11	100 *
T16	n.d.	n.d.	44.51 ± 1.75	93.84 ± 0.14	5.04 ± 0.45	100 *
T17	11.09 ± 2.56	n.d.	33.60 ± 2.10	93.84 ± 0.11	99.37 ± 0.17	100 *
T18	n.d.	25.48 ± 2.37	8.57 ± 2.89	93.74 ± 0.15	35.81 ± 1.95	100 *
T19	43.33 ± 1.97	40.99 ± 1.99	25.56 ± 2.36	93.68 ± 0.13	99.54 ± 0.12	95.40 ± 0.43
T20	51.66 ± 1.55	41.26 ± 1.91	10.89 ± 2.75	100 *	99.54 ± 0.14	78.41 ± 1.21
T21	40.26 ± 2.00	31.99 ± 2.16	11.00 ± 2.71	100 *	99.54 ± 0.12	83.45 ± 0.95
T22	47.97 ± 1.69	39.19 ± 2.04	31.13 ± 2.18	93.73 ± 0.14	99.54 ± 0.13	80.44 ± 1.10
T23	40.17 ± 2.04	5.52 ± 2.98	24.52 ± 2.42	100 *	94.20 ± 0.70	93.67 ± 0.57
T24	50.85 ± 1.52	11.67 ± 2.73	19.13 ± 2.63	100 *	99.54 ± 0.11	95.40 ± 0.36
T25	58.10 ± 1.30	11.04 ± 2.75	7.22 ± 2.94	100 *	99.54 ± 0.12	95.40 ± 0.34
T26	58.99 ± 1.25	50.41 ± 1.65	45.59 ± 1.73	100 *	99.54 ± 0.15	94.41 ± 0.48
T27	42.19 ± 1.92	43.51 ± 1.88	38.84 ± 1.95	100 *	98.50 ± 0.40	95.40 ± 0.39

Values are presented as mean ± SD (*n* = 3). DCV: daclatasvir; DRV: darunavir; FAV: favipiravir; LOP: lopinavir; REM: remdesivir; RIT: ritonavir; Exp: experimental run; TX: test X = number. n.d.—not detected; 100 *—values below the LOQ.

**Table A5:** Effective concentrations with 20% inhibition (***EC*_20_)** of tested SASs after 24 h of the removal process.

*EC*_20_, %						
Exp	DCV	DRV	FAV	LOP	REM	RIT
T1	15.66 ± 1.26	98.90 ± 1.10	n.d.	89.13 ± 0.87	26.72 ± 2.27	n.d.
T2	n.d.	19.11 ± 2.67	n.d.	21.86 ± 2.46	61.05 ± 1.49	3.91 ± 2.95
T3	31.11 ± 0.94	32.32 ± 1.88	n.d.	28.50 ± 2.28	75.28 ± 1.17	n.d.
T4	17.14 ± 1.12	51.59 ± 1.45	n.d.	74.06 ± 1.17	51.33 ± 1.53	n.d.
T5	32.77 ± 0.85	49.07 ± 1.56	n.d.	30.60 ± 2.11	n.d	n.d.
T6	20.33 ± 1.05	73.39 ± 1.13	n.d.	n.d.	57.79 ± 1.47	n.d.
T7	23.20 ± 0.98	97.48 ± 1.27	n.d.	58.73 ± 1.40	3.71 ± 3.07	n.d.
T8	7.24 ± 1.40	71.93 ± 1.11	n.d.	12.56 ± 2.71	27.81 ± 2.20	11.02 ± 2.75
T9	24.16 ± 0.92	53.72 ± 1.48	n.d.	91.17 ± 0.75	41.08 ± 1.83	n.d.
T10	25.06 ± 0.88	35.58 ± 1.73	n.d.	2.49 ± 3.11	86.00 ± 0.90	n.d.
T11	6.31 ± 1.51	35.24 ± 1.75	n.d.	16.17 ± 2.69	12.86 ± 2.77	n.d.
T12	n.d.	68.57 ± 1.28	n.d.	6.17 ± 2.97	44.54 ± 1.72	1.53 ± 3.00
T13	n.d.	n.d.	n.d.	36.78 ± 1.95	68.51 ± 1.25	n.d.
T14	n.d.	n.d.	n.d.	58.07 ± 1.48	95.64 ± 0.74	n.d.
T15	n.d.	26.60 ± 2.27	n.d.	52.53 ± 1.66	n.d.	n.d.
T16	n.d.	n.d.	n.d.	38.35 ± 1.94	71.85 ± 1.19	n.d.
T17	n.d.	n.d.	n.d.	35.35 ± 2.09	93.90 ± 0.78	n.d.
T18	n.d.	97.34 ± 0.41	n.d.	28.76 ± 2.22	70.37 ± 1.20	n.d.
T19	n.d.	48.19 ± 1.58	n.d.	5.22 ± 3.05	35.91 ± 1.98	n.d.
T20	15.81 ± 1.23	42.37 ± 1.69	n.d.	21.40 ± 2.50	39.29 ± 1.84	n.d.
T21	21.78 ± 1.02	40.63 ± 1.65	n.d.	78.66 ± 1.07	98.48 ± 0.71	n.d.
T22	n.d.	78.83 ± 1.03	n.d.	16.89 ± 2.61	29.93 ± 2.12	n.d.
T23	25.80 ± 0.85	n.d.	n.d.	10.60 ± 2.88	54.77 ± 1.58	n.d.
T24	11.98 ± 1.39	n.d.	n.d.	68.33 ± 1.27	81.99 ± 1.07	n.d.
T25	n.d.	n.d.	n.d.	34.44 ± 2.03	82.88 ± 1.03	n.d.
T26	9.85 ± 1.34	24.65 ± 2.34	27.94 ± 2.20	63.81 ± 1.38	49.54 ± 1.68	n.d.
T27	n.d.	25.61 ± 2.38	n.d.	62.43 ± 1.40	n.d.	n.d.

Values are presented as mean ± SD (*n* = 3). DCV: daclatasvir; DRV: darunavir; FAV: favipiravir; LOP: lopinavir; REM: remdesivir; RIT: ritonavir; Exp: experimental run; TX: test X = number. n.d.—not detected.

**Table A6:** Effective concentrations with 50% inhibition (***EC*_50_**) of tested SASs after 24 h of the removal process.

*EC_5_*_0_, %						
Exp	DCV	DRV	FAV	LOP	REM	RIT
T1	n.d.	n.d.	n.d.	n.d.	85.87 ± 0.80	n.d.
T2	23.63 ± 2.35	n.d.	n.d.	n.d.	93.89 ± 0.65	26.87 ± 2.10
T3	n.d.	n.d.	n.d.	n.d.	n.d.	n.d.
T4	n.d.	93.63 ± 0.70	n.d.	n.d.	n.d.	n.d.
T5	n.d.	91.90 ± 0.79	n.d.	n.d.	n.d.	n.d.
T6	n.d.	n.d.	n.d.	n.d.	n.d.	n.d.
T7	n.d.	n.d.	n.d.	n.d.	32.00 ± 2.05	n.d.
T8	n.d.	n.d.	n.d.	n.d.	n.d.	38.11 ± 1.95
T9	n.d.	n.d.	n.d.	n.d.	n.d.	n.d.
T10	n.d.	n.d.	n.d.	n.d.	n.d.	n.d.
T11	41.14 ± 1.83	n.d.	n.d.	n.d.	n.d.	n.d.
T12	n.d.	n.d.	n.d.	n.d.	n.d.	n.d.
T13	n.d.	n.d.	n.d.	n.d.	n.d.	n.d.
T14	n.d.	n.d.	n.d.	n.d.	n.d.	n.d.
T15	n.d.	n.d.	n.d.	n.d.	n.d.	n.d.
T16	n.d.	n.d.	n.d.	n.d.	n.d.	n.d.
T17	n.d.	n.d.	n.d.	96.94 ± 0.65	n.d.	n.d.
T18	n.d.	n.d.	n.d.	n.d.	n.d.	n.d.
T19	n.d.	n.d.	n.d.	22.41 ± 2.30	n.d.	n.d.
T20	40.10 ± 1.90	n.d.	n.d.	n.d.	n.d.	n.d.
T21	n.d.	n.d.	n.d.	n.d.	n.d.	n.d.
T22	n.d.	n.d.	n.d.	95.73 ± 0.70	45.98 ± 1.65	n.d.
T23	n.d.	n.d.	n.d.	24.96 ± 2.20	n.d.	n.d.
T24	42.19 ± 1.82	n.d.	n.d.	n.d.	n.d.	n.d.
T25	33.87 ± 2.05	n.d.	n.d.	72.57 ± 1.15	n.d.	n.d.
T26	33.98 ± 2.02	n.d.	n.d.	n.d.	n.d.	n.d.
T27	n.d.	22.53 ± 2.31	n.d.	87.73 ± 0.85	n.d.	n.d.

Values are presented as mean ± SD (*n* = 3). DCV: daclatasvir; DRV: darunavir; FAV: favipiravir; LOP: lopinavir; REM: remdesivir; RIT: ritonavir; Exp: experimental run; TX: test X = number. n.d.—not detected.

**Table A7:** (**a**) Dynamic variations of measured pH at 0 h, 6 h, and 24 h for tested SASs. (**b**) Dynamic variations of pH control at 0 h, 6 h, and 24 h for tested SASs.

(**a**)											
**Measured pH, -**											
**Exp**			***t*** **, h**			**DCV**	**DRV**	**FAV**	**LOP**	**REM**	**RIT**
T1			0			8.56 ± 0.04	8.59 ± 0.04	8.40 ± 0.02	8.75 ± 0.05	8.63 ± 0.02	8.66 ± 0.11
			6			8.50 ± 0.04	8.71 ± 0.06	8.34 ± 0.06	8.56 ± 0.03	8.55 ± 0.01	8.71 ± 0.06
			24			8.77 ± 0.07	8.53 ± 0.08	8.29 ± 0.07	8.42 ± 0.06	8.42 ± 0.03	8.70 ± 0.07
T2			0			6.18 ± 0.03	6.18 ± 0.03	5.81 ± 0.11	6.07 ± 0.05	6.30 ± 0.01	6.19 ± 0.05
			6			6.16 ± 0.04	6.49 ± 0.07	5.62 ± 0.05	5.98 ± 0.14	6.35 ± 0.14	6.21 ± 0.05
			24			6.29 ± 0.04	6.42 ± 0.04	5.81 ± 0.04	5.94 ± 0.06	6.36 ± 0.04	6.30 ± 0.04
T3			0			7.33 ± 0.03	7.19 ± 0.05	7.16 ± 0.09	7.37 ± 0.03	7.20 ± 0.01	7.62 ± 0.03
			6			7.24 ± 0.04	7.49 ± 0.14	7.15 ± 0.03	7.27 ± 0.06	7.15 ± 0.02	7.52 ± 0.03
			24			7.56 ± 0.06	7.34 ± 0.06	7.21 ± 0.06	7.21 ± 0.04	7.25 ± 0.03	7.72 ± 0.06
T4			0			6.16 ± 0.02	6.21 ± 0.05	5.78 ± 0.08	6.32 ± 0.05	6.13 ± 0.14	6.13 ± 0.12
			6			6.39 ± 0.03	6.56 ± 0.03	6.10 ± 0.06	6.27 ± 0.03	6.34 ± 0.03	6.38 ± 0.06
			24			6.65 ± 0.14	6.55 ± 0.07	6.21 ± 0.14	6.13 ± 0.06	6.36 ± 0.01	6.52 ± 0.14
T5			0			7.34 ± 0.04	7.19 ± 0.04	7.20 ± 0.02	7.37 ± 0.14	7.22 ± 0.02	7.59 ± 0.04
			6			7.26 ± 0.05	7.43 ± 0.12	7.21 ± 0.04	7.41 ± 0.05	7.36 ± 0.02	7.55 ± 0.04
			24			7.57 ± 0.05	7.30 ± 0.05	7.14 ± 0.05	7.04 ± 0.04	7.27 ± 0.04	7.64 ± 0.05
T6			0			8.57 ± 0.02	8.43 ± 0.06	8.43 ± 0.02	8.81 ± 0.06	8.29 ± 0.04	8.58 ± 0.13
			6			8.44 ± 0.11	8.68 ± 0.04	8.26 ± 0.04	8.60 ± 0.04	8.47 ± 0.01	8.60 ± 0.04
			24			8.71 ± 0.05	8.47 ± 0.02	8.22 ± 0.05	8.31 ± 0.09	8.32 ± 0.02	8.76 ± 0.05
T7			0			8.58 ± 0.02	8.66 ± 0.03	8.46 ± 0.04	8.81 ± 0.06	8.65 ± 0.01	8.63 ± 0.09
			6			8.56 ± 0.06	8.81 ± 0.06	8.42 ± 0.05	8.64 ± 0.03	8.67 ± 0.14	8.77 ± 0.05
			24			8.86 ± 0.04	8.64 ± 0.04	8.35 ± 0.04	8.37 ± 0.05	8.57 ± 0.03	8.84 ± 0.04
T8			0			6.15 ± 0.04	6.16 ± 0.05	5.74 ± 0.03	6.23 ± 0.04	6.13 ± 0.02	6.19 ± 0.03
			6			6.16 ± 0.07	6.47 ± 0.09	5.92 ± 0.03	6.17 ± 0.07	6.31 ± 0.02	6.33 ± 0.03
			24			6.44 ± 0.07	6.56 ± 0.03	6.11 ± 0.07	6.14 ± 0.03	6.30 ± 0.01	6.45 ± 0.07
T9			0			7.31 ± 0.05	7.21 ± 0.07	7.12 ± 0.04	7.30 ± 0.04	7.23 ± 0.02	7.61 ± 0.02
			6			7.27 ± 0.14	7.46 ± 0.02	7.16 ± 0.11	7.32 ± 0.07	7.29 ± 0.02	7.48 ± 0.11
			24			7.59 ± 0.03	7.37 ± 0.05	7.19 ± 0.03	7.08 ± 0.14	7.44 ± 0.05	7.63 ± 0.03
T10			0			6.10 ± 0.04	6.16 ± 0.05	5.84 ± 0.11	5.84 ± 0.07	5.80 ± 0.06	6.11 ± 0.03
			6			6.88 ± 0.03	6.47 ± 0.09	6.20 ± 0.03	5.89 ± 0.14	6.07 ± 0.03	6.68 ± 0.03
			24			6.84 ± 0.11	6.56 ± 0.03	6.26 ± 0.07	5.98 ± 0.04	6.16 ± 0.11	6.70 ± 0.07
T11			0			6.13 ± 0.03	7.21 ± 0.07	5.88 ± 0.05	5.87 ± 0.06	5.73 ± 0.04	6.08 ± 0.04
			6			6.49 ± 0.05	7.46 ± 0.02	6.13 ± 0.05	5.84 ± 0.03	5.92 ± 0.06	6.48 ± 0.05
			24			6.47 ± 0.08	7.37 ± 0.05	6.17 ± 0.04	5.91 ± 0.07	6.10 ± 0.14	6.50 ± 0.04
T12			0			6.11 ± 0.02	7.19 ± 0.05	5.86 ± 0.03	5.83 ± 0.05	5.79 ± 0.05	6.10 ± 0.02
			6			6.62 ± 0.06	7.49 ± 0.02	6.32 ± 0.06	5.99 ± 0.10	6.14 ± 0.03	6.77 ± 0.06
			24			6.73 ± 0.10	7.34 ± 0.06	6.42 ± 0.14	6.13 ± 0.04	6.19 ± 0.07	6.80 ± 0.14
T13			0			8.75 ± 0.04	8.43 ± 0.06	8.51 ± 0.12	8.58 ± 0.06	8.52 ± 0.04	8.68 ± 0.06
			6			8.87 ± 0.06	8.68 ± 0.04	8.44 ± 0.06	8.38 ± 0.03	8.46 ± 0.07	8.93 ± 0.06
			24			8.39 ± 0.09	8.47 ± 0.02	8.19 ± 0.07	8.46 ± 0.05	8.60 ± 0.05	8.68 ± 0.07
T14			0			7.54 ± 0.03	6.21 ± 0.05	7.16 ± 0.13	7.18 ± 0.04	7.02 ± 0.03	7.66 ± 0.12
			6			7.70 ± 0.11	6.56 ± 0.03	7.35 ± 0.11	7.13 ± 0.07	7.22 ± 0.06	7.80 ± 0.11
			24			7.69 ± 0.13	6.55 ± 0.07	7.27 ± 0.03	7.18 ± 0.11	7.34 ± 0.04	7.71 ± 0.03
T15			0			7.56 ± 0.05	8.66 ± 0.03	7.19 ± 0.06	7.18 ± 0.04	7.03 ± 0.04	7.58 ± 0.11
			6			7.60 ± 0.03	8.81 ± 0.06	7.34 ± 0.03	7.12 ± 0.01	7.21 ± 0.07	7.81 ± 0.03
			24			7.61 ± 0.06	8.64 ± 0.04	7.31 ± 0.06	7.21 ± 0.05	7.37 ± 0.05	7.70 ± 0.06
T16			0			8.77 ± 0.03	7.19 ± 0.04	8.54 ± 0.03	8.50 ± 0.05	8.49 ± 0.05	8.72 ± 0.05
			6			8.80 ± 0.04	7.43 ± 0.12	8.35 ± 0.04	8.26 ± 0.03	8.41 ± 0.01	8.74 ± 0.04
			24			8.33 ± 0.12	7.30 ± 0.05	8.20 ± 0.05	8.42 ± 0.06	8.70 ± 0.06	8.72 ± 0.05
T17			0			7.57 ± 0.04	8.59 ± 0.04	7.21 ± 0.03	7.10 ± 0.04	7.03 ± 0.05	7.63 ± 0.09
			6			7.69 ± 0.04	8.71 ± 0.06	7.37 ± 0.04	7.13 ± 0.05	7.19 ± 0.03	7.79 ± 0.04
			24			7.58 ± 0.07	8.53 ± 0.08	7.25 ± 0.05	7.21 ± 0.02	7.31 ± 0.07	7.70 ± 0.05
T18			0			8.79 ± 0.05	6.18 ± 0.03	8.56 ± 0.05	8.55 ± 0.06	8.53 ± 0.14	8.68 ± 0.06
			6			8.97 ± 0.05	6.49 ± 0.07	8.55 ± 0.05	8.36 ± 0.03	8.58 ± 0.04	8.96 ± 0.05
			24			8.47 ± 0.14	6.42 ± 0.04	8.30 ± 0.04	8.51 ± 0.05	8.63 ± 0.05	8.70 ± 0.04
T19			0			5.85 ± 0.03	5.98 ± 0.07	6.10 ± 0.11	5.82 ± 0.04	5.67 ± 0.04	5.82 ± 0.02
			6			6.12 ± 0.03	6.50 ± 0.14	6.63 ± 0.03	5.87 ± 0.07	6.11 ± 0.07	6.51 ± 0.03
			24			6.64 ± 0.07	6.35 ± 0.04	6.64 ± 0.07	6.04 ± 0.03	6.27 ± 0.03	6.66 ± 0.07
T20			0			7.86 ± 0.04	7.32 ± 0.05	7.60 ± 0.03	7.14 ± 0.03	7.16 ± 0.03	7.31 ± 0.12
			6			7.87 ± 0.04	7.64 ± 0.10	7.73 ± 0.04	7.03 ± 0.06	7.36 ± 0.06	7.67 ± 0.04
			24			8.08 ± 0.05	7.34 ± 0.04	7.61 ± 0.05	7.20 ± 0.04	7.37 ± 0.04	7.67 ± 0.05
T21			0			9.15 ± 0.05	8.73 ± 0.04	8.89 ± 0.13	5.90 ± 0.05	8.29 ± 0.05	8.62 ± 0.06
			6			9.18 ± 0.05	8.78 ± 0.07	9.06 ± 0.05	5.90 ± 0.03	8.63 ± 0.03	8.92 ± 0.05
			24			9.33 ± 0.04	8.60 ± 0.11	8.88 ± 0.04	6.00 ± 0.06	8.52 ± 0.06	8.96 ± 0.04
T22			0			5.88 ± 0.04	5.95 ± 0.06	6.15 ± 0.04	5.74 ± 0.05	5.67 ± 0.05	5.80 ± 0.03
			6			6.02 ± 0.05	6.40 ± 0.03	6.48 ± 0.05	5.75 ± 0.14	6.01 ± 0.14	6.39 ± 0.05
			24			6.43 ± 0.04	6.14 ± 0.05	6.41 ± 0.04	5.82 ± 0.06	6.10 ± 0.06	6.50 ± 0.04
T23			0			7.84 ± 0.03	7.27 ± 0.06	7.58 ± 0.05	7.12 ± 0.06	7.11 ± 0.06	7.27 ± 0.09
			6			7.76 ± 0.03	7.60 ± 0.03	7.64 ± 0.03	7.06 ± 0.04	7.34 ± 0.04	7.64 ± 0.03
			24			8.13 ± 0.06	7.42 ± 0.05	7.68 ± 0.06	7.03 ± 0.09	7.40 ± 0.09	7.74 ± 0.06
T24			0			9.13 ± 0.03	8.48 ± 0.05	8.95 ± 0.04	7.07 ± 0.14	8.44 ± 0.14	8.56 ± 0.05
			6			9.05 ± 0.04	8.75 ± 0.03	8.90 ± 0.04	7.06 ± 0.05	8.56 ± 0.05	8.86 ± 0.04
			24			9.34 ± 0.05	8.47 ± 0.06	8.88 ± 0.05	6.98 ± 0.04	8.64 ± 0.04	8.96 ± 0.05
T25			0			5.86 ± 0.05	6.07 ± 0.04	6.13 ± 0.02	8.41 ± 0.06	6.07 ± 0.06	5.81 ± 0.02
			6			6.09 ± 0.06	6.58 ± 0.01	6.68 ± 0.06	8.28 ± 0.03	6.21 ± 0.03	6.52 ± 0.06
			24			6.78 ± 0.14	6.38 ± 0.05	6.73 ± 0.14	8.49 ± 0.05	6.34 ± 0.05	6.79 ± 0.14
T26			0			9.11 ± 0.04	8.69 ± 0.04	8.93 ± 0.02	8.43 ± 0.04	8.45 ± 0.05	8.58 ± 0.07
			6			9.08 ± 0.06	8.42 ± 0.05	8.85 ± 0.06	8.27 ± 0.07	8.66 ± 0.03	8.86 ± 0.06
			24			9.33 ± 0.07	8.56 ± 0.02	8.84 ± 0.07	8.30 ± 0.04	8.55 ± 0.06	8.88 ± 0.07
T27			0			7.83 ± 0.05	7.29 ± 0.06	7.54 ± 0.12	8.44 ± 0.06	7.14 ± 0.04	7.21 ± 0.14
			6			7.53 ± 0.11	7.31 ± 0.03	7.75 ± 0.11	8.38 ± 0.03	7.39 ± 0.07	7.72 ± 0.11
			24			8.20 ± 0.03	7.32 ± 0.07	7.64 ± 0.03	8.24 ± 0.05	7.38 ± 0.14	7.71 ± 0.03
(**b**)											
**pH Control, -**											
**pH**		**Exp**		***t*** **, h**		**DCV**	**DRV**	**FAV**	**LOP**	**REM**	**RIT**
5.5		C(−1)		0		6.16 ± 0.04	6.29 ± 0.04	5.77 ± 0.05	6.23 ± 0.05	6.00 ± 0.02	6.17 ± 0.05
				6		6.05 ± 0.05	6.50 ± 0.05	5.43 ± 0.04	6.33 ± 0.03	6.40 ± 0.14	6.16 ± 0.04
				24		6.38 ± 0.05	6.67 ± 0.03	5.62 ± 0.05	6.18 ± 0.06	6.34 ± 0.03	6.32 ± 0.05
		C0		0		6.11 ± 0.05	6.21 ± 0.05	5.86 ± 0.05	5.88 ± 0.04	5.78 ± 0.04	6.10 ± 0.05
				6		6.41 ± 0.04	6.38 ± 0.04	5.99 ± 0.04	5.99 ± 0.06	6.14 ± 0.06	6.47 ± 0.04
				24		6.59 ± 0.12	6.62 ± 0.06	6.04 ± 0.05	6.11 ± 0.03	6.17 ± 0.03	6.50 ± 0.05
		C1		0		5.86 ± 0.05	5.96 ± 0.04	6.13 ± 0.05	5.76 ± 0.05	5.91 ± 0.05	5.81 ± 0.05
				6		6.19 ± 0.04	6.34 ± 0.06	6.41 ± 0.04	5.85 ± 0.03	6.04 ± 0.03	6.35 ± 0.04
				24		6.54 ± 0.05	6.21 ± 0.03	6.50 ± 0.05	5.87 ± 0.06	6.34 ± 0.06	6.41 ± 0.05
7.0		C(−1)		0		7.32 ± 0.06	7.24 ± 0.06	7.15 ± 0.03	7.35 ± 0.04	7.27 ± 0.01	7.61 ± 0.03
				6		7.33 ± 0.01	7.44 ± 0.01	7.21 ± 0.07	7.30 ± 0.07	7.45 ± 0.02	7.44 ± 0.07
				24		7.55 ± 0.03	7.58 ± 0.04	7.22 ± 0.03	7.18 ± 0.01	7.26 ± 0.05	7.56 ± 0.03
		C0		0		7.55 ± 0.03	7.27 ± 0.03	7.19 ± 0.03	7.23 ± 0.05	7.03 ± 0.05	7.62 ± 0.03
				6		7.74 ± 0.07	7.39 ± 0.07	7.33 ± 0.07	7.12 ± 0.01	7.22 ± 0.01	7.80 ± 0.07
				24		7.54 ± 0.09	7.58 ± 0.01	7.28 ± 0.03	7.19 ± 0.06	7.19 ± 0.06	7.62 ± 0.03
		C1		0		7.84 ± 0.03	7.27 ± 0.05	7.58 ± 0.03	7.04 ± 0.04	7.13 ± 0.04	7.26 ± 0.03
				6		7.78 ± 0.07	7.52 ± 0.01	7.72 ± 0.07	7.04 ± 0.07	7.29 ± 0.07	7.57 ± 0.07
				24		8.10 ± 0.03	7.37 ± 0.06	7.60 ± 0.03	6.94 ± 0.01	7.36 ± 0.01	7.70 ± 0.03
8.5		C(−1)		0		8.47 ± 0.03	8.67 ± 0.07	8.43 ± 0.06	8.72 ± 0.05	8.75 ± 0.02	8.62 ± 0.06
				6		8.72 ± 0.06	8.88 ± 0.03	8.35 ± 0.14	8.56 ± 0.04	8.73 ± 0.14	8.68 ± 0.14
				24		8.12 ± 0.06	8.89 ± 0.11	8.21 ± 0.06	8.55 ± 0.11	8.51 ± 0.04	8.61 ± 0.06
		C0		0		8.77 ± 0.06	8.64 ± 0.05	8.54 ± 0.06	8.66 ± 0.04	8.55 ± 0.04	8.70 ± 0.06
				6		8.89 ± 0.14	8.68 ± 0.14	8.42 ± 0.14	8.42 ± 0.07	8.49 ± 0.02	8.84 ± 0.14
				24		8.612 ± 0.15	8.83 ± 0.12	8.32 ± 0.06	8.44 ± 0.14	8.61 ± 0.10	8.60 ± 0.06
		C1		0		9.13 ± 0.06	8.75 ± 0.04	8.93 ± 0.06	8.36 ± 0.05	8.48 ± 0.05	8.59 ± 0.06
				6		9.03 ± 0.14	8.85 ± 0.07	8.70 ± 0.14	8.38 ± 0.04	8.66 ± 0.04	8.70 ± 0.14
				24		9.32 ± 0.06	8.66 ± 0.07	8.86 ± 0.06	8.23 ± 0.11	8.55 ± 0.11	8.94 ± 0.06

Values are presented as mean ± SD (*n* = 3). DCV: daclatasvir; DRV: darunavir; FAV: favipiravir; LOP: lopinavir; REM: remdesivir; RIT: ritonavir; Exp: experimental run; TX: test X = number; CX: control X = MLSS level.

**Table A8:** (**a**) Dynamic variations of measured DO at 0 h, 6 h, and 24 h for tested SASs. (**b**) Dynamic variations of DO control at 0 h, 6 h, and 24 h for tested SASs.

(**a**)											
**Measured DO, mg/L**											
**Exp**			***t*** **, h**			**DCV**	**DRV**	**FAV**	**LOP**	**REM**	**RIT**
T1			0			7.69 ± 0.38	7.68 ± 0.35	7.82 ± 0.29	6.90 ± 0.31	6.25 ± 0.29	6.39 ± 0.16
			6			9.73 ± 0.44	6.59 ± 0.30	8.60 ± 0.39	6.28 ± 0.28	5.19 ± 0.24	7.40 ± 0.34
			24			6.93 ± 0.31	8.31 ± 0.38	9.46 ± 0.43	7.61 ± 0.35	5.22 ± 0.24	5.66 ± 0.26
T2			0			5.21 ± 0.12	7.67 ± 0.35	5.18 ± 0.35	8.36 ± 0.38	8.22 ± 0.38	7.35 ± 0.29
			6			8.30 ± 0.38	7.23 ± 0.33	6.21 ± 0.28	7.82 ± 0.36	9.37 ± 0.43	8.79 ± 0.41
			24			5.92 ± 0.27	8.85 ± 0.40	8.61 ± 0.39	8.22 ± 0.37	8.38 ± 0.38	8.87 ± 0.42
T3			0			5.24 ± 0.14	7.77 ± 0.35	5.09 ± 0.39	6.33 ± 0.29	8.88 ± 0.41	6.33 ± 0.18
			6			6.76 ± 0.31	6.47 ± 0.29	4.09 ± 0.19	6.91 ± 0.31	8.38 ± 0.38	6.53 ± 0.30
			24			4.92 ± 0.22	8.40 ± 0.38	7.59 ± 0.35	8.14 ± 0.37	8.17 ± 0.37	6.70 ± 0.31
T4			0			7.51 ± 0.32	6.82 ± 0.31	5.36 ± 0.42	7.55 ± 0.34	6.03 ± 0.28	7.17 ± 0.32
			6			8.93 ± 0.40	5.02 ± 0.23	6.77 ± 0.31	7.58 ± 0.34	5.60 ± 0.26	7.83 ± 0.35
			24			6.23 ± 0.28	7.55 ± 0.34	8.80 ± 0.40	8.22 ± 0.37	4.23 ± 0.19	7.95 ± 0.36
T5			0			5.19 ± 0.16	8.61 ± 0.39	7.62 ± 0.13	7.97 ± 0.36	7.41 ± 0.34	7.24 ± 0.26
			6			6.75 ± 0.31	6.74 ± 0.31	7.50 ± 0.34	8.13 ± 0.37	7.81 ± 0.36	7.40 ± 0.34
			24			2.40 ± 0.38	8.29 ± 0.38	9.01 ± 0.41	8.57 ± 0.39	7.48 ± 0.34	5.66 ± 0.26
T6			0			7.55 ± 0.41	7.48 ± 0.34	7.59 ± 0.18	6.60 ± 0.30	6.23 ± 0.29	5.79 ± 0.43
			6			9.57 ± 0.43	5.26 ± 0.24	7.20 ± 0.33	6.98 ± 0.31	5.07 ± 0.23	4.11 ± 0.19
			24			6.00 ± 0.27	7.70 ± 0.35	9.02 ± 0.41	6.59 ± 0.30	4.63 ± 0.21	7.05 ± 0.32
T7			0			7.60 ± 0.25	8.04 ± 0.37	7.65 ± 0.15	7.63 ± 0.35	7.07 ± 0.32	5.54 ± 0.38
			6			9.26 ± 0.42	7.10 ± 0.32	7.68 ± 0.35	7.36 ± 0.34	7.46 ± 0.34	6.53 ± 0.30
			24			6.48 ± 0.29	8.75 ± 0.40	9.20 ± 0.42	8.34 ± 0.38	6.35 ± 0.29	6.70 ± 0.31
T8			0			7.88 ± 0.44	7.72 ± 0.35	7.98 ± 0.38	7.89 ± 0.36	6.43 ± 0.30	5.40 ± 0.41
			6			10.13 ± 0.46	7.84 ± 0.36	9.91 ± 0.45	7.66 ± 0.35	7.54 ± 0.35	6.35 ± 0.29
			24			8.51 ± 0.39	8.38 ± 0.38	8.81 ± 0.40	8.71 ± 0.40	7.64 ± 0.35	7.72 ± 0.35
T9			0			7.74 ± 0.28	8.12 ± 0.37	7.71 ± 0.31	7.11 ± 0.32	6.47 ± 0.30	6.28 ± 0.14
			6			9.88 ± 0.45	7.03 ± 0.32	8.82 ± 0.40	7.02 ± 0.32	5.89 ± 0.27	7.23 ± 0.33
			24			8.22 ± 0.37	8.26 ± 0.38	9.48 ± 0.43	8.61 ± 0.39	6.05 ± 0.28	7.20 ± 0.33
T10			0			8.04 ± 0.16	6.87 ± 0.31	8.39 ± 0.13	8.14 ± 0.37	6.65 ± 0.30	6.26 ± 0.35
			6			6.60 ± 0.30	3.52 ± 0.16	7.62 ± 0.35	7.74 ± 0.36	4.93 ± 0.22	6.61 ± 0.31
			24			8.24 ± 0.22	4.90 ± 0.22	6.05 ± 0.27	7.15 ± 0.33	5.62 ± 0.26	6.13 ± 0.28
T11			0			7.98 ± 0.14	7.65 ± 0.35	7.38 ± 0.40	8.53 ± 0.39	7.79 ± 0.36	6.15 ± 0.31
			6			6.40 ± 0.29	4.26 ± 0.19	6.28 ± 0.28	7.80 ± 0.36	6.49 ± 0.30	6.17 ± 0.29
			24			8.69 ± 0.15	4.17 ± 0.19	6.34 ± 0.29	7.87 ± 0.36	6.48 ± 0.30	5.75 ± 0.26
T12			0			8.01 ± 0.18	6.57 ± 0.30	7.49 ± 0.36	6.58 ± 0.30	6.79 ± 0.31	5.64 ± 0.42
			6			6.04 ± 0.27	4.76 ± 0.21	6.17 ± 0.28	6.70 ± 0.31	4.42 ± 0.20	5.36 ± 0.24
			24			7.87 ± 0.19	6.29 ± 0.28	4.70 ± 0.21	6.65 ± 0.30	4.48 ± 0.20	4.65 ± 0.20
T13			0			7.74 ± 0.32	6.80 ± 0.31	8.44 ± 0.15	7.37 ± 0.34	7.81 ± 0.36	5.91 ± 0.16
			6			6.50 ± 0.29	3.71 ± 0.17	7.78 ± 0.36	6.61 ± 0.30	4.30 ± 0.20	6.12 ± 0.27
			24			10.26 ± 0.27	4.27 ± 0.19	6.63 ± 0.30	7.43 ± 0.34	4.89 ± 0.22	7.00 ± 0.32
T14			0			7.51 ± 0.28	6.71 ± 0.30	7.66 ± 0.43	7.35 ± 0.34	7.30 ± 0.33	5.84 ± 0.14
			6			6.58 ± 0.30	3.25 ± 0.15	6.53 ± 0.30	6.40 ± 0.29	4.50 ± 0.21	6.02 ± 0.28
			24			9.08 ± 0.25	4.34 ± 0.20	5.70 ± 0.26	7.21 ± 0.33	5.41 ± 0.25	5.80 ± 0.27
T15			0			6.72 ± 0.41	6.47 ± 0.29	7.21 ± 0.28	6.88 ± 0.31	6.52 ± 0.30	5.87 ± 0.18
			6			3.66 ± 0.17	2.88 ± 0.44	5.62 ± 0.25	5.47 ± 0.25	4.93 ± 0.22	5.19 ± 0.22
			24			8.57 ± 0.31	3.97 ± 0.18	3.20 ± 0.15	6.99 ± 0.32	5.07 ± 0.23	4.79 ± 0.22
T16			0			7.63 ± 0.30	6.07 ± 0.27	8.32 ± 0.18	7.36 ± 0.34	7.19 ± 0.33	6.21 ± 0.27
			6			6.58 ± 0.30	3.46 ± 0.16	7.21 ± 0.33	5.88 ± 0.27	5.61 ± 0.26	6.08 ± 0.29
			24			10.86 ± 0.34	3.42 ± 0.16	7.23 ± 0.33	7.22 ± 0.33	4.69 ± 0.21	6.43 ± 0.30
T17			0			7.08 ± 0.44	7.45 ± 0.34	7.03 ± 0.31	7.77 ± 0.35	7.27 ± 0.33	5.35 ± 0.45
			6			6.88 ± 0.31	5.05 ± 0.23	4.17 ± 0.19	7.60 ± 0.35	6.26 ± 0.28	4.25 ± 0.19
			24			8.46 ± 0.38	4.95 ± 0.22	5.70 ± 0.26	7.25 ± 0.33	6.60 ± 0.30	5.82 ± 0.26
T18			0			6.88 ± 0.39	7.19 ± 0.33	7.12 ± 0.24	8.02 ± 0.37	8.22 ± 0.38	5.47 ± 0.38
			6			4.73 ± 0.21	3.69 ± 0.17	5.17 ± 0.23	6.67 ± 0.31	6.09 ± 0.28	4.10 ± 0.18
			24			10.42 ± 0.39	5.71 ± 0.26	2.11 ± 0.36	7.64 ± 0.35	6.98 ± 0.32	4.18 ± 0.19
T19			0			7.37 ± 0.18	8.44 ± 0.38	7.44 ± 0.44	7.78 ± 0.36	8.22 ± 0.38	6.76 ± 0.14
			6			6.33 ± 0.29	3.23 ± 0.15	7.01 ± 0.32	7.50 ± 0.34	7.05 ± 0.32	6.35 ± 0.32
			24			6.03 ± 0.27	6.21 ± 0.28	7.13 ± 0.33	6.69 ± 0.31	5.20 ± 0.24	6.21 ± 0.28
T20			0			8.51 ± 0.36	8.97 ± 0.41	7.91 ± 0.24	7.12 ± 0.33	8.24 ± 0.38	7.28 ± 0.38
			6			7.32 ± 0.33	4.79 ± 0.22	8.21 ± 0.38	6.92 ± 0.32	5.79 ± 0.26	6.58 ± 0.33
			24			6.76 ± 0.31	5.68 ± 0.26	6.24 ± 0.28	5.87 ± 0.27	6.28 ± 0.28	6.17 ± 0.35
T21			0			6.69 ± 0.42	8.17 ± 0.37	7.26 ± 0.37	7.36 ± 0.34	8.10 ± 0.37	6.82 ± 0.16
			6			5.40 ± 0.24	5.14 ± 0.23	5.76 ± 0.26	7.30 ± 0.34	5.77 ± 0.26	6.12 ± 0.24
			24			4.12 ± 0.18	5.72 ± 0.26	3.13 ± 0.15	7.13 ± 0.33	6.52 ± 0.30	2.64 ± 0.45
T22			0			8.22 ± 0.39	8.78 ± 0.40	7.15 ± 0.41	7.25 ± 0.33	8.50 ± 0.39	7.52 ± 0.41
			6			6.89 ± 0.31	4.99 ± 0.23	7.03 ± 0.32	7.01 ± 0.32	6.88 ± 0.31	6.41 ± 0.29
			24			4.86 ± 0.21	6.30 ± 0.29	4.26 ± 0.19	5.64 ± 0.26	5.86 ± 0.27	5.47 ± 0.24
T23			0			6.38 ± 0.45	7.64 ± 0.35	5.70 ± 0.15	6.82 ± 0.31	7.88 ± 0.36	5.08 ± 0.25
			6			5.23 ± 0.23	1.08 ± 0.42	5.79 ± 0.26	6.69 ± 0.31	6.25 ± 0.28	3.40 ± 0.18
			24			6.36 ± 0.29	4.32 ± 0.20	3.81 ± 0.17	7.12 ± 0.33	4.61 ± 0.21	3.35 ± 0.17
T24			0			6.49 ± 0.38	7.14 ± 0.32	5.63 ± 0.17	7.22 ± 0.33	7.64 ± 0.35	6.78 ± 0.18
			6			5.99 ± 0.27	1.20 ± 0.46	5.43 ± 0.25	6.16 ± 0.28	2.66 ± 0.45	6.17 ± 0.21
			24			6.21 ± 0.28	4.63 ± 0.21	5.09 ± 0.23	7.54 ± 0.35	4.72 ± 0.21	4.03 ± 0.21
T25			0			8.34 ± 0.31	7.89 ± 0.36	5.66 ± 0.12	6.52 ± 0.30	7.53 ± 0.34	7.41 ± 0.35
			6			6.57 ± 0.30	1.59 ± 0.43	7.11 ± 0.33	6.16 ± 0.28	5.34 ± 0.24	6.53 ± 0.30
			24			7.06 ± 0.32	4.13 ± 0.19	6.64 ± 0.30	6.74 ± 0.31	4.69 ± 0.21	6.47 ± 0.32
T26			0			7.41 ± 0.16	7.75 ± 0.35	7.98 ± 0.28	6.38 ± 0.29	6.91 ± 0.31	5.01 ± 0.31
			6			6.57 ± 0.30	2.02 ± 0.38	4.93 ± 0.22	6.78 ± 0.31	2.96 ± 0.44	4.79 ± 0.52
			24			6.58 ± 0.30	4.58 ± 0.21	5.09 ± 0.23	6.10 ± 0.28	5.96 ± 0.27	3.03 ± 0.15
T27			0			7.34 ± 0.14	8.07 ± 0.37	7.87 ± 0.31	7.28 ± 0.33	7.82 ± 0.36	5.14 ± 0.28
			6			6.01 ± 0.27	1.30 ± 0.44	7.08 ± 0.32	6.22 ± 0.28	3.45 ± 0.16	5.62 ± 0.27
			24			3.76 ± 0.16	5.22 ± 0.24	4.20 ± 0.19	7.87 ± 0.36	5.61 ± 0.26	2.30 ± 0.38
(**b**)											
**DO Control, mg/L**											
***T***		**Exp**		***t*** **, h**		**DCV**	**DRV**	**FAV**	**LOP**	**REM**	**RIT**
10 °C		C(−1)		0		7.61 ± 0.35	8.39 ± 0.38	7.83 ± 0.35	7.41 ± 0.34	7.80 ± 0.36	7.25 ± 0.33
				6		9.97 ± 0.45	7.08 ± 0.32	9.10 ± 0.42	6.77 ± 0.31	7.45 ± 0.34	8.28 ± 0.38
				24		8.23 ± 0.37	8.23 ± 0.37	8.51 ± 0.39	8.03 ± 0.37	7.70 ± 0.35	8.20 ± 0.38
		C0		0		8.01 ± 0.36	7.66 ± 0.35	8.39 ± 0.38	7.79 ± 0.36	7.62 ± 0.35	6.20 ± 0.28
				6		7.35 ± 0.33	4.88 ± 0.22	7.63 ± 0.35	7.66 ± 0.35	8.49 ± 0.02	7.32 ± 0.34
				24		8.83 ± 0.32	6.81 ± 0.31	6.54 ± 0.30	8.12 ± 0.37	8.61 ± 0.10	6.21 ± 0.29
		C1		0		8.35 ± 0.38	8.63 ± 0.39	7.92 ± 0.36	7.31 ± 0.34	8.29 ± 0.38	7.39 ± 0.34
				6		7.45 ± 0.34	4.65 ± 0.21	7.42 ± 0.34	7.65 ± 0.35	5.18 ± 0.24	6.80 ± 0.31
				24		7.28 ± 0.33	6.28 ± 0.28	7.59 ± 0.35	7.36 ± 0.34	6.14 ± 0.28	5.74 ± 0.26
30 °C		C(−1)		0		7.70 ± 0.35	7.75 ± 0.35	7.62 ± 0.34	7.37 ± 0.34	7.20 ± 0.33	6.33 ± 0.29
				6		8.99 ± 0.41	6.75 ± 0.31	7.50 ± 0.34	7.18 ± 0.33	7.12 ± 0.33	7.80 ± 0.36
				24		4.70 ± 0.21	8.03 ± 0.37	9.13 ± 0.42	8.46 ± 0.39	6.85 ± 0.31	7.74 ± 0.35
		C0		0		7.61 ± 0.34	7.89 ± 0.36	7.50 ± 0.34	7.73 ± 0.35	7.68 ± 0.35	5.87 ± 0.26
				6		4.80 ± 0.22	5.78 ± 0.26	6.53 ± 0.30	6.19 ± 0.28	5.72 ± 0.26	4.78 ± 0.42
				24		9.37 ± 0.28	5.16 ± 0.23	5.49 ± 0.25	6.52 ± 0.30	4.80 ± 0.22	5.76 ± 0.25
		C1		0		7.37 ± 0.34	8.20 ± 0.38	7.28 ± 0.33	7.15 ± 0.33	7.41 ± 0.34	6.79 ± 0.28
				6		5.42 ± 0.25	1.16 ± 0.45	6.15 ± 0.28	7.30 ± 0.34	2.36 ± 0.43	5.64 ± 0.25
				24		6.08 ± 0.28	3.64 ± 0.16	4.75 ± 0.21	6.95 ± 0.32	5.17 ± 0.23	4.14 ± 0.19
45 °C		C(−1)		0		5.21 ± 0.23	7.07 ± 0.32	5.20 ± 0.23	7.02 ± 0.32	6.61 ± 0.30	5.57 ± 0.25
				6		8.92 ± 0.40	6.83 ± 0.31	6.55 ± 0.29	7.29 ± 0.33	6.69 ± 0.31	5.30 ± 0.44
				24		3.22 ± 0.43	7.55 ± 0.34	8.31 ± 0.38	7.93 ± 0.36	6.47 ± 0.30	7.20 ± 0.33
		C0		0		6.89 ± 0.31	7.08 ± 0.32	7.11 ± 0.32	7.31 ± 0.34	6.44 ± 0.30	5.48 ± 0.23
				6		3.04 ± 0.42	5.04 ± 0.23	3.31 ± 0.42	6.80 ± 0.31	5.82 ± 0.27	2.80 ± 0.48
				24		10.91 ± 0.35	6.08 ± 0.27	4.82 ± 0.22	6.20 ± 0.28	4.73 ± 0.22	4.67 ± 0.21
		C1		0		6.51 ± 0.29	8.47 ± 0.39	5.66 ± 0.26	6.29 ± 0.29	7.81 ± 0.36	5.07 ± 0.22
				6		6.22 ± 0.28	4.15 ± 0.19	2.41 ± 0.43	6.75 ± 0.31	6.57 ± 0.30	2.14 ± 0.41
				24		3.18 ± 0.43	4.28 ± 0.19	4.50 ± 0.20	8.30 ± 0.38	5.25 ± 0.24	4.18 ± 0.22

Values are presented as mean ± SD (*n* = 3). DCV: daclatasvir; DRV: darunavir; FAV: favipiravir; LOP: lopinavir; REM: remdesivir; RIT: ritonavir; Exp: experimental run; TX: test X = number; CX: control X = MLSS level.

**Table A9:** (**a**) Dynamic variations of measured EC at 0 h, 6 h, and 24 h for tested SASs. (**b**) Dynamic variations of EC control at 0 h, 6 h, and 24 h for tested SASs.

(**a**)											
**Measured EC, mS/cm**											
**Exp**			***t*** **, h**			**DCV**	**DRV**	**FAV**	**LOP**	**REM**	**RIT**
T1			0			14.22 ± 0.16	17.67 ± 0.33	17.52 ± 0.18	17.23 ± 0.31	17.17 ± 0.31	16.90 ± 0.14
			6			10.50 ± 0.17	17.53 ± 0.28	14.19 ± 0.25	17.22 ± 0.29	16.92 ± 0.29	9.97 ± 0.14
			24			10.45 ± 0.17	17.60 ± 0.31	12.99 ± 0.22	17.30 ± 0.30	16.88 ± 0.31	9.87 ± 0.15
T2			0			7.62 ± 0.08	10.34 ± 0.16	12.86 ± 0.08	10.51 ± 0.18	10.74 ± 0.16	10.24 ± 0.11
			6			17.76 ± 0.31	10.28 ± 0.14	17.91 ± 0.31	10.46 ± 0.16	10.78 ± 0.16	17.38 ± 0.30
			24			17.65 ± 0.31	10.41 ± 0.21	17.83 ± 0.31	9.84 ± 0.15	10.87 ± 0.17	17.52 ± 0.30
T3			0			14.04 ± 0.18	12.04 ± 0.22	12.81 ± 0.06	14.01 ± 0.25	11.00 ± 0.18	12.81 ± 0.14
			6			17.16 ± 0.30	11.92 ± 0.19	17.26 ± 0.30	13.85 ± 0.22	10.88 ± 0.12	16.79 ± 0.29
			24			16.99 ± 0.30	12.00 ± 0.24	17.24 ± 0.30	14.01 ± 0.26	10.88 ± 0.17	16.54 ± 0.28
T4			0			7.58 ± 0.06	9.75 ± 0.14	10.19 ± 0.12	9.69 ± 0.14	9.79 ± 0.15	10.21 ± 0.13
			6			13.14 ± 0.23	9.73 ± 0.11	12.43 ± 0.21	9.78 ± 0.16	9.62 ± 0.14	12.40 ± 0.21
			24			13.06 ± 0.23	9.88 ± 0.19	12.40 ± 0.21	9.90 ± 0.15	9.70 ± 0.14	12.32 ± 0.20
T5			0			8.64 ± 0.07	12.79 ± 0.23	10.28 ± 0.11	14.41 ± 0.25	12.56 ± 0.22	12.69 ± 0.16
			6			14.81 ± 0.26	12.73 ± 0.21	9.80 ± 0.15	15.00 ± 0.27	12.51 ± 0.27	9.67 ± 0.15
			24			14.55 ± 0.26	12.79 ± 0.26	17.24 ± 0.30	15.05 ± 0.28	12.41 ± 0.21	9.61 ± 0.14
T6			0			8.58 ± 0.06	17.34 ± 0.32	17.23 ± 0.08	16.75 ± 0.31	16.59 ± 0.30	17.07 ± 0.18
			6			13.48 ± 0.24	16.84 ± 0.29	13.05 ± 0.23	16.71 ± 0.29	16.20 ± 0.28	12.82 ± 0.27
			24			13.40 ± 0.24	16.86 ± 0.42	13.07 ± 0.23	17.40 ± 0.32	16.12 ± 0.28	12.76 ± 0.21
T7			0			14.11 ± 0.13	18.29 ± 0.34	17.31 ± 0.19	17.97 ± 0.33	17.64 ± 0.32	16.85 ± 0.19
			6			18.27 ± 0.33	18.18 ± 0.30	18.56 ± 0.33	17.83 ± 0.37	17.54 ± 0.37	17.99 ± 0.31
			24			17.07 ± 0.31	18.26 ± 0.32	18.39 ± 0.33	17.99 ± 0.32	17.47 ± 0.30	17.83 ± 0.31
T8			0			7.54 ± 0.09	10.02 ± 0.18	10.38 ± 0.14	10.24 ± 0.17	10.02 ± 0.15	10.15 ± 0.08
			6			10.76 ± 0.18	10.00 ± 0.15	17.86 ± 0.32	10.11 ± 0.14	9.95 ± 0.14	10.21 ± 0.16
			24			10.74 ± 0.18	10.10 ± 0.17	17.91 ± 0.31	10.16 ± 0.16	9.98 ± 0.14	10.15 ± 0.16
T9			0			8.61 ± 0.05	12.12 ± 0.22	12.79 ± 0.07	14.50 ± 0.26	12.22 ± 0.21	12.74 ± 0.11
			6			13.90 ± 0.25	12.46 ± 0.25	13.45 ± 0.24	14.52 ± 0.25	12.11 ± 0.20	13.22 ± 0.22
			24			13.83 ± 0.25	12.61 ± 0.24	13.43 ± 0.24	14.54 ± 0.27	12.06 ± 0.20	9.90 ± 0.15
T10			0			10.80 ± 0.12	9.95 ± 0.15	10.31 ± 0.11	9.90 ± 0.15	9.81 ± 0.15	10.18 ± 0.12
			6			10.68 ± 0.18	9.96 ± 0.13	9.25 ± 0.15	10.03 ± 0.13	9.83 ± 0.14	10.17 ± 0.16
			24			6.07 ± 0.25	10.20 ± 0.21	6.20 ± 0.11	10.49 ± 0.21	10.18 ± 0.18	10.29 ± 0.16
T11			0			10.69 ± 0.10	10.27 ± 0.16	10.23 ± 0.08	10.31 ± 0.16	10.10 ± 0.16	10.09 ± 0.08
			6			9.94 ± 0.15	10.27 ± 0.15	10.10 ± 0.16	10.31 ± 0.15	10.18 ± 0.16	9.92 ± 0.15
			24			6.28 ± 0.19	11.52 ± 0.38	10.81 ± 0.17	10.35 ± 0.16	10.83 ± 0.19	10.12 ± 0.16
T12			0			10.73 ± 0.08	9.66 ± 0.14	10.26 ± 0.12	9.58 ± 0.14	9.46 ± 0.15	10.12 ± 0.14
			6			5.62 ± 0.11	9.66 ± 0.12	9.81 ± 0.15	9.58 ± 0.12	9.59 ± 0.14	9.60 ± 0.15
			24			5.83 ± 0.12	9.97 ± 0.19	10.08 ± 0.16	10.43 ± 0.19	10.00 ± 0.17	9.83 ± 0.16
T13			0			16.89 ± 0.18	17.73 ± 0.32	17.50 ± 0.16	17.25 ± 0.32	17.85 ± 0.33	17.04 ± 0.19
			6			13.82 ± 0.24	17.29 ± 0.26	11.71 ± 0.20	17.24 ± 0.26	17.42 ± 0.29	13.22 ± 0.22
			24			5.06 ± 0.26	17.44 ± 0.30	12.47 ± 0.21	18.23 ± 0.30	17.90 ± 0.32	13.74 ± 0.23
T14			0			13.31 ± 0.07	12.69 ± 0.21	13.11 ± 0.09	12.71 ± 0.21	12.22 ± 0.21	12.73 ± 0.14
			6			13.20 ± 0.23	12.73 ± 0.24	13.18 ± 0.23	12.74 ± 0.24	12.15 ± 0.20	12.85 ± 0.21
			24			5.41 ± 0.18	13.27 ± 0.36	19.80 ± 0.35	13.00 ± 0.26	12.93 ± 0.25	12.99 ± 0.22
T15			0			13.35 ± 0.11	12.24 ± 0.20	13.01 ± 0.13	12.14 ± 0.20	11.52 ± 0.20	12.61 ± 0.16
			6			12.27 ± 0.21	12.18 ± 0.18	12.76 ± 0.21	12.28 ± 0.18	11.65 ± 0.19	12.35 ± 0.21
			24			4.93 ± 0.29	12.50 ± 0.23	13.15 ± 0.22	12.68 ± 0.23	11.93 ± 0.22	12.46 ± 0.21
T16			0			17.11 ± 0.16	16.92 ± 0.29	17.29 ± 0.19	16.79 ± 0.29	17.49 ± 0.31	16.82 ± 0.18
			6			18.18 ± 0.32	16.70 ± 0.25	17.81 ± 0.32	16.41 ± 0.25	16.90 ± 0.28	17.95 ± 0.31
			24			4.72 ± 0.17	17.30 ± 0.33	18.55 ± 0.33	16.96 ± 0.33	17.77 ± 0.32	18.18 ± 0.31
T17			0			13.41 ± 0.09	13.09 ± 0.24	13.05 ± 0.07	13.10 ± 0.24	12.51 ± 0.22	12.66 ± 0.11
			6			17.79 ± 0.31	13.06 ± 0.21	17.32 ± 0.31	13.16 ± 0.22	12.57 ± 0.27	17.36 ± 0.30
			24			5.57 ± 0.21	13.66 ± 0.27	18.69 ± 0.33	13.93 ± 0.27	13.00 ± 0.24	18.02 ± 0.33
T18			0			16.97 ± 0.14	17.95 ± 0.33	17.35 ± 0.12	17.73 ± 0.33	18.12 ± 0.33	16.88 ± 0.15
			6			15.91 ± 0.27	17.88 ± 0.28	14.91 ± 0.25	17.73 ± 0.28	18.13 ± 0.32	16.90 ± 0.29
			24			5.18 ± 0.14	18.64 ± 0.44	15.48 ± 0.26	18.74 ± 0.44	18.49 ± 0.36	17.53 ± 0.28
T19			0			9.64 ± 0.12	10.20 ± 0.16	10.25 ± 0.11	10.12 ± 0.18	10.31 ± 0.16	10.26 ± 0.13
			6			13.06 ± 0.22	10.01 ± 0.14	13.24 ± 0.22	10.06 ± 0.15	10.20 ± 0.14	13.53 ± 0.23
			24			13.87 ± 0.24	10.26 ± 0.18	13.76 ± 0.23	10.78 ± 0.17	12.23 ± 0.23	14.28 ± 0.24
T20			0			12.92 ± 0.14	12.81 ± 0.22	12.77 ± 0.14	17.54 ± 0.32	13.42 ± 0.24	13.06 ± 0.14
			6			16.02 ± 0.27	12.70 ± 0.18	10.21 ± 0.16	13.25 ± 0.24	14.26 ± 0.26	10.27 ± 0.16
			24			18.49 ± 0.32	12.83 ± 0.25	10.49 ± 0.17	15.07 ± 0.28	16.60 ± 0.32	10.60 ± 0.17
T21			0			16.94 ± 0.19	17.48 ± 0.32	16.96 ± 0.18	17.76 ± 0.32	18.18 ± 0.34	17.44 ± 0.19
			6			16.82 ± 0.29	17.16 ± 0.28	9.58 ± 0.15	17.79 ± 0.31	18.33 ± 0.32	9.68 ± 0.15
			24			15.61 ± 0.27	17.54 ± 0.30	10.00 ± 0.15	18.42 ± 0.34	22.10 ± 0.46	10.19 ± 0.16
T22			0			9.53 ± 0.09	10.40 ± 0.15	10.14 ± 0.13	10.33 ± 0.16	10.51 ± 0.18	10.32 ± 0.11
			6			15.32 ± 0.26	10.27 ± 0.13	9.97 ± 0.15	10.44 ± 0.15	10.52 ± 0.16	10.00 ± 0.15
			24			18.08 ± 0.31	10.48 ± 0.19	10.33 ± 0.16	11.24 ± 0.21	11.82 ± 0.22	10.74 ± 0.18
T23			0			12.81 ± 0.16	12.14 ± 0.21	12.66 ± 0.09	12.36 ± 0.22	12.75 ± 0.22	12.94 ± 0.16
			6			16.42 ± 0.28	11.90 ± 0.19	16.85 ± 0.29	12.63 ± 0.20	12.50 ± 0.20	17.48 ± 0.30
			24			18.76 ± 0.32	12.29 ± 0.23	17.45 ± 0.29	12.93 ± 0.24	13.55 ± 0.25	19.09 ± 0.33
T24			0			17.00 ± 0.13	16.53 ± 0.29	16.74 ± 0.19	17.23 ± 0.31	16.97 ± 0.31	17.22 ± 0.18
			6			13.02 ± 0.22	16.20 ± 0.25	12.83 ± 0.21	16.82 ± 0.29	17.27 ± 0.29	13.09 ± 0.22
			24			14.12 ± 0.25	16.68 ± 0.34	14.65 ± 0.25	17.44 ± 0.30	17.85 ± 0.33	14.76 ± 0.25
T25			0			9.57 ± 0.11	9.88 ± 0.14	10.21 ± 0.08	9.65 ± 0.14	9.93 ± 0.14	10.21 ± 0.08
			6			17.97 ± 0.31	9.71 ± 0.12	17.91 ± 0.31	9.96 ± 0.15	10.20 ± 0.16	18.27 ± 0.33
			24			18.95 ± 0.33	9.91 ± 0.17	18.17 ± 0.31	10.95 ± 0.19	10.92 ± 0.19	18.92 ± 0.33
T26			0			17.15 ± 0.18	16.99 ± 0.31	16.80 ± 0.15	17.55 ± 0.32	17.66 ± 0.32	17.30 ± 0.15
			6			15.78 ± 0.27	16.56 ± 0.27	17.95 ± 0.31	17.63 ± 0.31	17.40 ± 0.30	18.32 ± 0.33
			24			16.62 ± 0.29	16.95 ± 0.30	17.90 ± 0.30	18.04 ± 0.33	20.30 ± 0.38	19.78 ± 0.35
T27			0			12.88 ± 0.11	12.53 ± 0.22	12.69 ± 0.12	12.93 ± 0.23	12.94 ± 0.23	12.98 ± 0.11
			6			13.02 ± 0.22	12.30 ± 0.19	12.51 ± 0.21	12.75 ± 0.27	12.84 ± 0.27	12.64 ± 0.27
(**b**)											
**EC Control, mg/L**											
***T***		**Exp**		***t*** **, h**		**DCV**	**DRV**	**FAV**	**LOP**	**REM**	**RIT**
10 °C		C(−1)		0		14.12 ± 0.25	18.35 ± 0.35	17.40 ± 0.30	18.00 ± 0.34	18.16 ± 0.34	16.93 ± 0.29
				6		13.55 ± 0.24	18.15 ± 0.31	17.16 ± 0.30	17.43 ± 0.32	18.05 ± 0.32	16.80 ± 0.28
				24		13.50 ± 0.24	18.20 ± 0.33	9.85 ± 0.16	18.05 ± 0.33	18.07 ± 0.32	16.70 ± 0.28
		C0		0		16.99 ± 0.30	18.00 ± 0.34	17.38 ± 0.30	17.81 ± 0.34	18.05 ± 0.34	16.90 ± 0.29
				6		16.76 ± 0.29	17.94 ± 0.31	17.56 ± 0.30	17.99 ± 0.37	18.49 ± 0.02	16.66 ± 0.28
				24		4.76 ± 0.22	18.18 ± 0.35	11.35 ± 0.19	18.71 ± 0.35	18.61 ± 0.10	16.94 ± 0.29
		C1		0		17.02 ± 0.30	17.52 ± 0.33	16.82 ± 0.29	17.66 ± 0.33	18.26 ± 0.34	17.31 ± 0.30
				6		16.94 ± 0.29	17.23 ± 0.29	14.12 ± 0.24	18.20 ± 0.31	17.97 ± 0.31	17.18 ± 0.30
				24		19.13 ± 0.34	17.54 ± 0.31	18.36 ± 0.32	18.69 ± 0.35	21.40 ± 0.45	17.15 ± 0.29
30 °C		C(−1)		0		8.61 ± 0.15	12.39 ± 0.22	12.82 ± 0.22	14.48 ± 0.26	12.15 ± 0.22	12.75 ± 0.21
				6		8.67 ± 0.15	12.28 ± 0.20	12.89 ± 0.22	14.41 ± 0.23	12.03 ± 0.20	12.78 ± 0.21
				24		8.64 ± 0.15	12.31 ± 0.25	14.21 ± 0.25	14.47 ± 0.25	12.02 ± 0.20	12.82 ± 0.20
		C0		0		13.36 ± 0.23	12.78 ± 0.22	13.06 ± 0.22	12.60 ± 0.22	12.09 ± 0.22	12.67 ± 0.21
				6		13.30 ± 0.23	12.61 ± 0.19	13.05 ± 0.22	12.65 ± 0.19	11.98 ± 0.19	12.73 ± 0.27
				24		5.88 ± 0.15	12.85 ± 0.25	16.83 ± 0.29	13.15 ± 0.25	12.17 ± 0.23	14.02 ± 0.24
		C1		0		12.87 ± 0.22	11.92 ± 0.19	12.71 ± 0.21	12.60 ± 0.22	13.26 ± 0.23	13.00 ± 0.22
				6		13.10 ± 0.22	11.85 ± 0.17	13.30 ± 0.22	12.94 ± 0.20	13.12 ± 0.21	13.07 ± 0.22
				24		14.84 ± 0.26	12.28 ± 0.24	16.50 ± 0.28	13.70 ± 0.25	15.04 ± 0.28	15.95 ± 0.27
45 °C		C(−1)		0		7.58 ± 0.13	9.62 ± 0.15	10.23 ± 0.17	9.82 ± 0.16	9.61 ± 0.16	10.20 ± 0.16
				6		7.74 ± 0.14	9.59 ± 0.12	17.85 ± 0.32	9.72 ± 0.13	9.44 ± 0.13	10.22 ± 0.17
				24		7.70 ± 0.14	9.49 ± 0.18	17.99 ± 0.32	9.77 ± 0.15	9.60 ± 0.15	10.21 ± 0.16
		C0		0		10.74 ± 0.18	9.76 ± 0.16	10.27 ± 0.16	9.76 ± 0.16	9.66 ± 0.16	10.13 ± 0.16
				6		10.75 ± 0.18	9.69 ± 0.13	10.37 ± 0.17	9.57 ± 0.13	9.51 ± 0.13	10.15 ± 0.16
				24		6.64 ± 0.28	9.79 ± 0.17	11.25 ± 0.19	9.92 ± 0.17	9.65 ± 0.15	10.39 ± 0.17
		C1		0		9.58 ± 0.16	10.11 ± 0.16	10.20 ± 0.16	9.51 ± 0.16	10.12 ± 0.16	10.27 ± 0.16
				6		9.72 ± 0.16	9.98 ± 0.14	10.24 ± 0.16	9.83 ± 0.14	10.11 ± 0.14	10.53 ± 0.17
				24		10.49 ± 0.18	10.50 ± 0.21	10.66 ± 0.17	10.50 ± 0.21	10.77 ± 0.21	11.32 ± 0.19

Values are presented as mean ± SD (*n* = 3). DCV: daclatasvir; DRV: darunavir; FAV: favipiravir; LOP: lopinavir; REM: remdesivir; RIT: ritonavir; Exp: experimental run; TX: test X = number; CX: control X = MLSS level.

**Table A10:** Analysis of Variance (ANOVA) for RSM models of five responses (Removal, %): (**a**) DRV, (**b**) FAV, (**c**) LOP, and (**d**) RIT.

Source	*SS*	d*f*	*MS*	*F*-Value	*p*-Value	
(**a**) Reduced 2FI Model	4399.75	5	879.95	4.66	0.0051	significant
A-pH	505.25	1	505.25	2.68	0.1166	
B-MLSS	1766.2	1	1766.2	9.36	0.0059	
C-T	64.38	1	64.38	0.3413	0.5653	
AC	984.7	1	984.7	5.22	0.0328	
BC	1120.72	1	1120.72	5.94	0.0238	
Residual	3961.83	21	188.66			
Lack of Fit	3960.87	19	208.47	437.86	0.0023	significant
Pure Error	0.9522	2	0.4761			
Cor Total	8361.57	26				
(**b**) Reduced Quadratic Model	2147.31	8	268.41	10.31	<0.0001	significant
A-pH	1416.08	1	1416.08	54.4	<0.0001	
B-MLSS	123.01	1	123.01	4.73	0.0433	
C-T	127.53	1	127.53	4.9	0.04	
AB	139.54	1	139.54	5.36	0.0326	
AC	139.41	1	139.41	5.36	0.0327	
BC	141.03	1	141.03	5.42	0.0318	
A^2^	127.17	1	127.17	4.89	0.0403	
C^2^	17.22	1	17.22	0.6614	0.4267	
Residual	468.54	18	26.03			
Lack of Fit	467.44	16	29.22	53.35	0.0185	significant
Pure Error	1.1	2	0.5476			
Cor Total	2615.84	26				
(**c**) Quadratic Model	181.12	9	20.12	13.23	<0.0001	significant
A-pH	9.55	1	9.55	6.28	0.0215	
B-MLSS	1.68	1	1.68	1.1	0.3068	
C-T	105.85	1	105.85	69.58	<0.0001	
AB	2.95	1	2.95	1.94	0.1801	
AC	10.41	1	10.41	6.84	0.017	
BC	3.22	1	3.22	2.12	0.1621	
A^2^	2.99	1	2.99	1.97	0.1768	
B^2^	1.12	1	1.12	0.7342	0.4022	
C^2^	54.13	1	54.13	35.58	<0.0001	
Residual	28.9	19	1.52			
Lack of Fit	28.85	17	1.7	66.3	0.015	significant
Pure Error	0.0512	2	0.0256			
Cor Total	210.02	28				
(**d**) Quadratic Model	791.51	9	87.95	8.46	<0.0001	significant
A-pH	4.29	1	4.29	0.4126	0.5283	
B-MLSS	141.95	1	141.95	13.66	0.0015	
C-T	34.06	1	34.06	3.28	0.0861	
AB	1.04	1	1.04	0.0998	0.7555	
AC	12.59	1	12.59	1.21	0.2848	
BC	70.19	1	70.19	6.75	0.0176	
A^2^	10.02	1	10.02	0.9643	0.3385	
B^2^	27.96	1	27.96	2.69	0.1174	
C^2^	475.76	1	475.76	45.78	<0.0001	
Residual	197.44	19	10.39			
Lack of Fit	197.43	17	11.61	3484.1	0.0003	significant
Pure Error	0.0067	2	0.0033			
Cor Total	988.95	28				

Design-Expert 12 software package was used for data modeling and statistical.
